# Supplementary material for: Ion mobility-tandem mass spectrometry of mucin-type O-glycans
Source: Nat Commun. 2024 Mar 23;15:2611. doi: 10.1038/s41467-024-46825-4 (PMC10960840; doi:10.1038/s41467-024-46825-4)
Supplement: Supplementary file 1 — Reporting summary [file 41467_2024_46825_MOESM1_ESM.pdf]

nature portfolio

Corresponding author(s): Pr Kevin Pagel

Last updated by author(s): Feb 23, 2024

Reporting Summary

Nature Portfolio wishes to improve the reproducibility of the work that we publish. This form provides structure for consistency and transparency in reporting. For further information on Nature Portfolio policies, see our Editorial Policies and the Editorial Policy Checklist.

Statistics

For all statistical analyses, confirm that the following items are present in the figure legend, table legend, main text, or Methods section.

n/a	Confirmed	
☐	☒	The exact sample size (n) for each experimental group/condition, given as a discrete number and unit of measurement
☐	☒	A statement on whether measurements were taken from distinct samples or whether the same sample was measured repeatedly
☒	☐	The statistical test(s) used AND whether they are one- or two-sided<br>*Only common tests should be described solely by name; describe more complex techniques in the Methods section.*
☒	☐	A description of all covariates tested
☒	☐	A description of any assumptions or corrections, such as tests of normality and adjustment for multiple comparisons
☐	☒	A full description of the statistical parameters including central tendency (e.g. means) or other basic estimates (e.g. regression coefficient) AND variation (e.g. standard deviation) or associated estimates of uncertainty (e.g. confidence intervals)
☐	☒	For null hypothesis testing, the test statistic (e.g. F, t, r) with confidence intervals, effect sizes, degrees of freedom and P value noted<br>*Give P values as exact values whenever suitable.*
☒	☐	For Bayesian analysis, information on the choice of priors and Markov chain Monte Carlo settings
☒	☐	For hierarchical and complex designs, identification of the appropriate level for tests and full reporting of outcomes
☒	☐	Estimates of effect sizes (e.g. Cohen's d, Pearson's r), indicating how they were calculated

Our web collection on statistics for biologists contains articles on many of the points above.

Software and code

Policy information about availability of computer code

Data collection	The LC-MS/MS and TWIMS data was acquired using MassLynx (version 2.0.7, Waters). The TIM-MS and TIM-MS/MS data was acquired with Compass otofControl (version 6.2, Bruker). The proteomics data was acquired using Thermo EASY-nLC 1200: 3.2.0 SP2 (version 4.1.4.1), Orbitrap Exploris 480 (tune software version 3.1.279.9) and Xcalibur (version 4.6.67.17).
Data analysis	The LC-MS/MS and TWIMS data was processed using MassLynx (version 2.0.7, Waters). LC chromatograms were deconvoluted and integrated with MZmine 2. TWIMS arrival time distributions were fitted to a Gaussian distribution with Origin 95E. The TIM-MS and TIM-MS/MS data was processed using DataAnalysis (version 4.0, Bruker). All O-glycans structures were manually assigned using GlycoWorkBench 2.1. For protein identification, MaxQuant software (version 2.0.3.0; Max Planck Institute of Biochemistry) and a decoy human UniProt database (2022-01) were used. Downstream analysis was done in R (version 4.0.4).

For manuscripts utilizing custom algorithms or software that are central to the research but not yet described in published literature, software must be made available to editors and reviewers. We strongly encourage code deposition in a community repository (e.g. GitHub). See the Nature Portfolio guidelines for submitting code & software for further information.

Data

Policy information about availability of data

All manuscripts must include a data availability statement. This statement should provide the following information, where applicable:

- Accession codes, unique identifiers, or web links for publicly available datasets
- A description of any restrictions on data availability
- For clinical datasets or third party data, please ensure that the statement adheres to our policy

The raw mass spectrometry data of patient samples are protected and are not available due to data privacy laws.
The processed glycomics data generated in this study, including O-glycan structures, CCSs and MS/MS spectra, have been deposited in the UniCarb-DR database under accession codes:
533 [https://unicarb-dr.glycosmos.org/references/533] (PGM); 534 [https://unicarb-dr.glycosmos.org/references/534] (healthy sputum); 535 [https://unicarb-dr.glycosmos.org/references/535] (CF sputum).
The processed glycomics data generated in this study, including the annotated MS/MS spectra of O-glycan structures identified, are provided in the Supplementary Data 1.
The processed glycomics data generated in this study, including the identified O-glycan structures with CCS, putative structure, area ratios from PGCLC-TWIMS and TIMS data, are provided in the Supplementary Data 2.
The processed proteomics data generated in this study, including protein intensities reported by the MaxQuant identification software, are provided in the Supplementary Data 3.
Source data are provided with this paper. The source data underlying Figure 6 are provided as a Source Data file.

Research involving human participants, their data, or biological material

Policy information about studies with human participants or human data. See also policy information about sex, gender (identity/presentation), and sexual orientation and race, ethnicity and racism.

Reporting on sex and gender	Sex and gender were not considered in the study design. The clinical donors of healthy and cystic fibrosis sputum were both of female sex and gender, and consent has been obtained for sharing of individual-level data.
Reporting on race, ethnicity, or other socially relevant groupings	Race, ethnicity, or other social categorization were not considered.
Population characteristics	The cystic fibrosis sputum donor was of age 33 at the time of sample collection, with chronic pseudomonas and presence of Staph. Auerus reported for this patient. The healthy control was of age 28 at the time of sample collection and had no history of any chronic lung disease.
Recruitment	The cystic fibrosis patient was recruited with an exclusion criteria of history of smoking, acute respiratory infection or pulmonary exacerbation, history of organ transplantation, or prior exposure to CFTR modulator treatment. The Cystic Fibrosis Center at Charité provides clinical care to more than 300 patients with cystic fibrosis and all patients are offered to donate sputum to a sputum biobank for research following provision of informed consent under the ethics vote. The sample from the single patient used for the experiments reported in this manuscript was randomly selected using the exclusion criteria mentioned to exclude bias by known confounding factors. The sample from the healthy individual was matched for sex and age and otherwise randomly selected from a biobank of healthy sputum. A written consent to participate in the study was provided by all donors.
Ethics oversight	Ethics committee of Charité - Universitätsmedizin Berlin (EA2/016/18)

Note that full information on the approval of the study protocol must also be provided in the manuscript.

Field-specific reporting

Please select the one below that is the best fit for your research. If you are not sure, read the appropriate sections before making your selection.

☒ Life sciences ☐ Behavioural & social sciences ☐ Ecological, evolutionary & environmental sciences

For a reference copy of the document with all sections, see nature.com/documents/nr-reporting-summary-flat.pdf

Life sciences study design

All studies must disclose on these points even when the disclosure is negative.

Sample size	The ion mobility-based glycomics method was validated using commercially available porcine gastric mucins (PGM, Sigma). No sample size calculation was performed. This sample was selected for its highly heterogeneous but well-characterized O-glycosylation. It is considered sufficient for this study as it provides about 50 O-glycan structures covering diverse glycosylation features (sialylation, fucosylation, sulfation) and a wide range of glycan sizes and structures, which is particularly interesting for this study to show the isomers separation by ion mobility. Two clinical samples of healthy and cystic fibrosis sputum were selected for a proof-of-principle of the analytical workflow. Cystic fibrosis sputum was selected as it induces drastic modifications in the patients O-glycosylation, to verify that the method is able to discriminate

	between healthy and diseased states.
Data exclusions	No data were excluded from the analysis.
Replication	Concerning the data reported for porcine gastric mucin O-glycans used for method development; LC retention times were acquired in duplicates, TWIMS data were acquired in duplicates, and TIMS data were acquired in triplicates. Attempts at replication were successful, with standard deviations below 0.5% for the 3 techniques, indicated in the supporting information table. The TIMS data on the human sputum samples was successfully reproduced once (standard deviation are not indicated).
Randomization	Randomization is not relevant in the method development part as only one sample was measured (PGM). The data obtained from the 2 human sputum samples were acquired in random order.
Blinding	Blinding is not relevant in this study due to the size of sample set.

Reporting for specific materials, systems and methods

We require information from authors about some types of materials, experimental systems and methods used in many studies. Here, indicate whether each material, system or method listed is relevant to your study. If you are not sure if a list item applies to your research, read the appropriate section before selecting a response.

Materials & experimental systems

n/a	Involved in the study
☒	☐ Antibodies
☒	☐ Eukaryotic cell lines
☒	☐ Palaeontology and archaeology
☒	☐ Animals and other organisms
☒	☐ Clinical data
☒	☐ Dual use research of concern
☒	☐ Plants

Methods

n/a	Involved in the study
☒	☐ ChIP-seq
☒	☐ Flow cytometry
☒	☐ MRI-based neuroimaging

Plants

Seed stocks	*Report on the source of all seed stocks or other plant material used. If applicable, state the seed stock centre and catalogue number. If plant specimens were collected from the field, describe the collection location, date and sampling procedures.*
Novel plant genotypes	*Describe the methods by which all novel plant genotypes were produced. This includes those generated by transgenic approaches, gene editing, chemical/radiation-based mutagenesis and hybridization. For transgenic lines, describe the transformation method, the number of independent lines analyzed and the generation upon which experiments were performed. For gene-edited lines, describe the editor used, the endogenous sequence targeted for editing, the targeting guide RNA sequence (if applicable) and how the editor was applied.*
Authentication	*Describe any authentication procedures for each seed stock used or novel genotype generated. Describe any experiments used to assess the effect of a mutation and, where applicable, how potential secondary effects (e.g. second site T-DNA insertions, mosiacism, off-target gene editing) were examined.*
