# Supplementary material for: Ion mobility-tandem mass spectrometry of mucin-type O-glycans
Source: Nat Commun. 2024 Mar 23;15:2611. doi: 10.1038/s41467-024-46825-4 (PMC10960840; doi:10.1038/s41467-024-46825-4)
Supplement: Supplementary file 2 — Description of Additional Supplementary Files [file 41467_2024_46825_MOESM2_ESM.pdf]

Description of additional supplementary Information files

Supplementary Data 1 (PDF). Annotated MS/MS spectra of *O*-glycan structures identified in *porcine* gastric mucins, healthy sputum and cystic fibrosis sputum. PGC retention times, $^{TWIMS}CCS_{N2}$ and $^{TIMS}CCS_{N2}$ are also indicated.

Supplementary Data 2 (Excel). Processed glycomics data generated in this study, including the identified *O*-glycan structures with $^{TWIMS}CCS_{N2}$ and $^{TIMS}CCS_{N2}$, putative structure, area ratios from $_{PGC}$LC-TWIMS and TIMS data.

Supplementary Data 3 (Excel). Protein intensities output reported by the MaxQuant identification software for the proteomics data on the sputum samples.
