# Supplementary material for: Ion mobility-tandem mass spectrometry of mucin-type O-glycans
Source: Nat Commun. 2024 Mar 23;15:2611. doi: 10.1038/s41467-024-46825-4 (PMC10960840; doi:10.1038/s41467-024-46825-4)

Supplementary Data 1:

Ion Mobility-Tandem Mass Spectrometry of Mucin-type *O*-Glycans

Leïla Bechtella,[1,2] Chunsheng Jin,[3] Kerstin Fentker,[1,4] Güney R. Ertürk,[1] Marc Safferthal,[1,2] Łukasz Polewski,[1,2] Michael Götze,[1,2] Simon Y. Graeber,[5,6,7] Gaël M. Vos,[1,2] Weston B. Struwe,[8,9] Marcus A. Mall, [5,6,7] Philipp Mertins,[4,10] Niclas G. Karlsson,[3,11] Kevin Pagel[1,2,*]

[1]Institute of Chemistry and Biochemistry, Freie Universität Berlin, Altensteinstraße 23A, 14195 Berlin, Germany. [2]Fritz Haber Institute of the Max Planck Society, Faradayweg 4-6, 14195 Berlin, Germany. [3]Department of Medical Biochemistry and Cell Biology, Institute of Biomedicine, Sahlgrenska Academy, University of Gothenburg, Sweden. [4]Max Delbrück Center for Molecular Medicine, Robert-Rössle-Str. 10, 13125 Berlin, Germany. [5]Department of Pediatric Respiratory Medicine, Immunology and Critical Care Medicine and Cystic Fibrosis Center, Charité – Universitätsmedizin Berlin, corporate member of Freie Universität Berlin and Humboldt-Universität zu Berlin, Berlin, Germany. [6]German Center for Lung Research (DZL), associated partner site, Berlin, Germany. [7]Berlin Institute of Health at Charité – Universitätsmedizin Berlin, Berlin, Germany. [8]Kavli Institute for Nanoscience Discovery, University of Oxford, Oxford OX1 3QU, United Kingdom. [9]Department of Biochemistry, University of Oxford, Oxford OX1 3QU, United Kingdom. [10]Berlin Institute of Health, 10178 Berlin, Germany. [11]Department of Life Sciences and Health, Faculty of Health Sciences, Oslo Metropolitan University, Oslo, Norway.
*email: kevin.pagel@fu-berlin.de

Table of contents

I. Annotated MS/MS spectra of *O*-glycans in *porcine* gastric mucins S2
II. Annotated MS/MS spectra of *O*-glycans in healthy sputum S29
III. Annotated MS/MS spectra of *O*-glycans in cystic fibrosis sputum S44

I. Annotated MS/MS spectra of *O*-glycans in *porcine* gastric mucins

Glycan code: 530-1

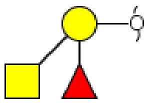

m/z: 530.2
RT: 15.6 min
^{TWIMS}CCS: 207 Å
^{TIMS}CCS: 208 Å

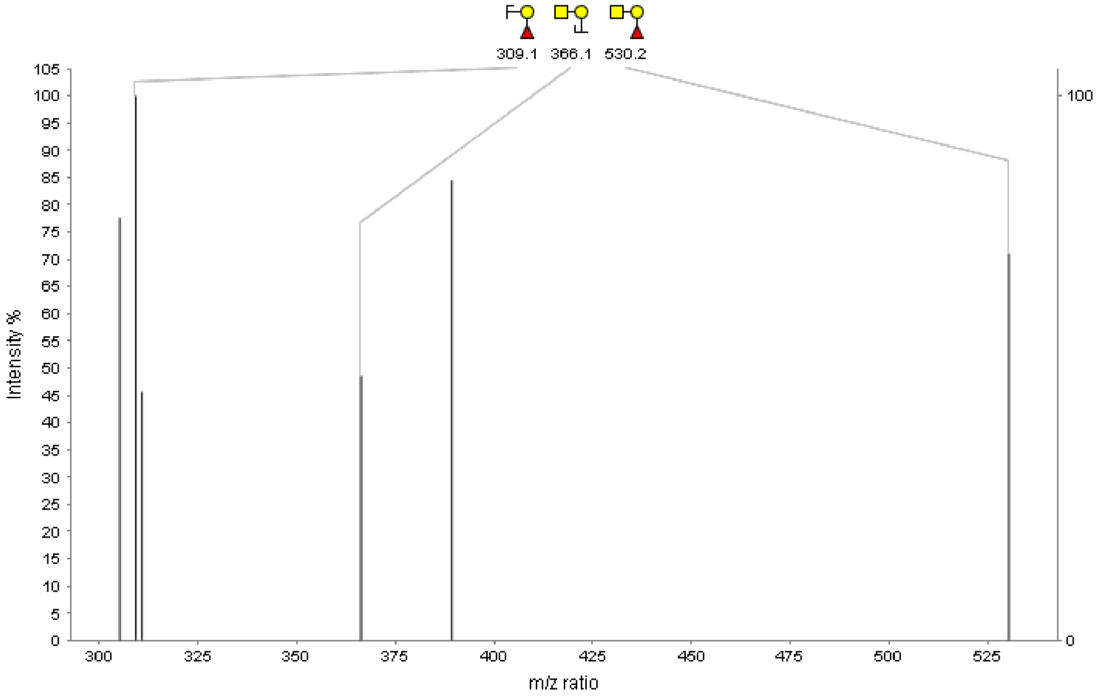

Glycan code: 530-2

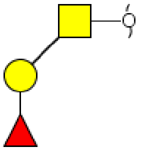

m/z: 530.2
RT: 25.5 min
^{TWIMS}CCS: 209 Å
^{TIMS}CCS: 210 Å

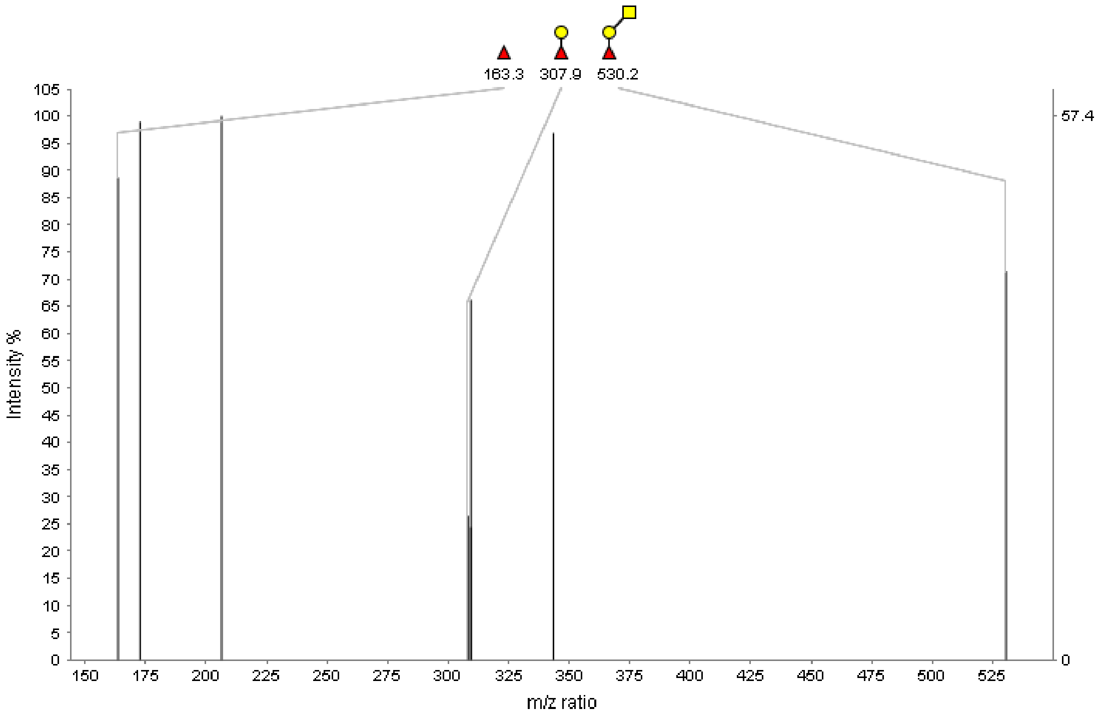

Glycan code: 587-1

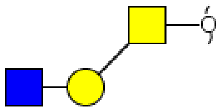

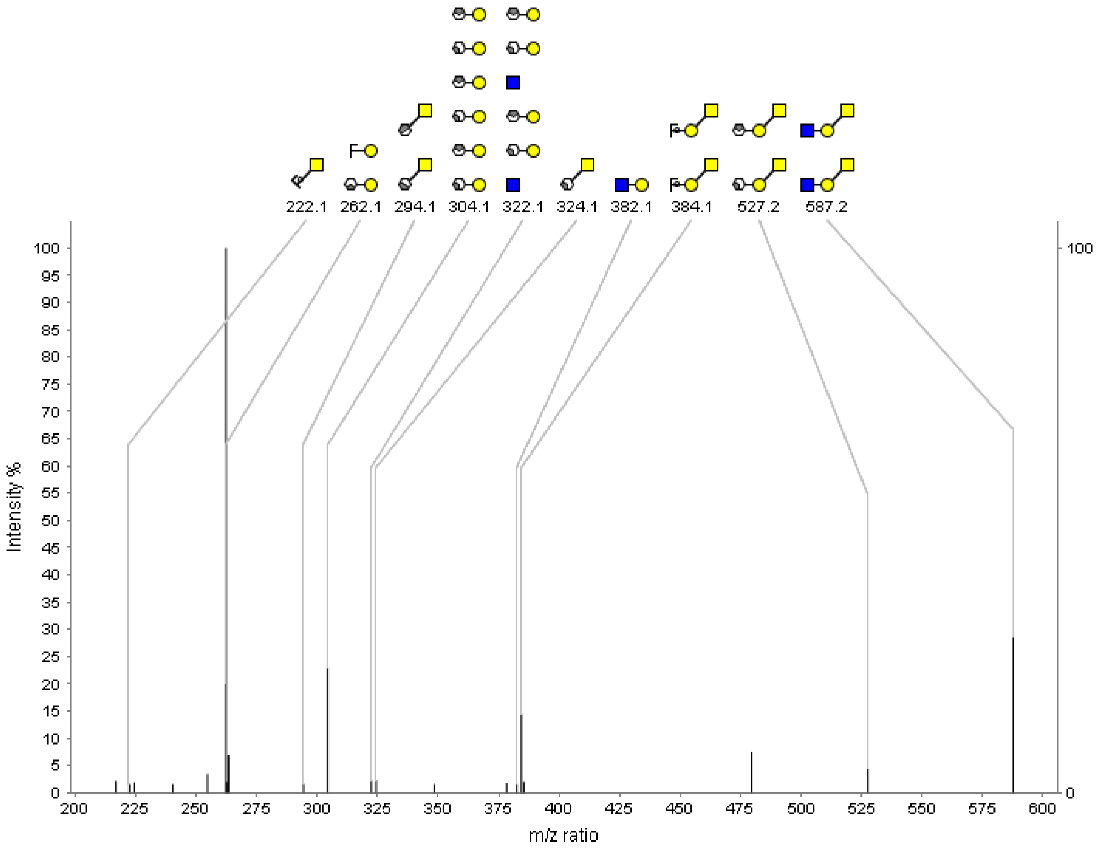

m/z: 587.2
RT: 14.3 min
^{TWIMS}CCS: 215 Å
^{TIMS}CCS: 216 Å

Glycan code: 587-2

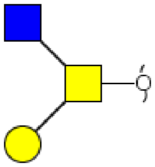

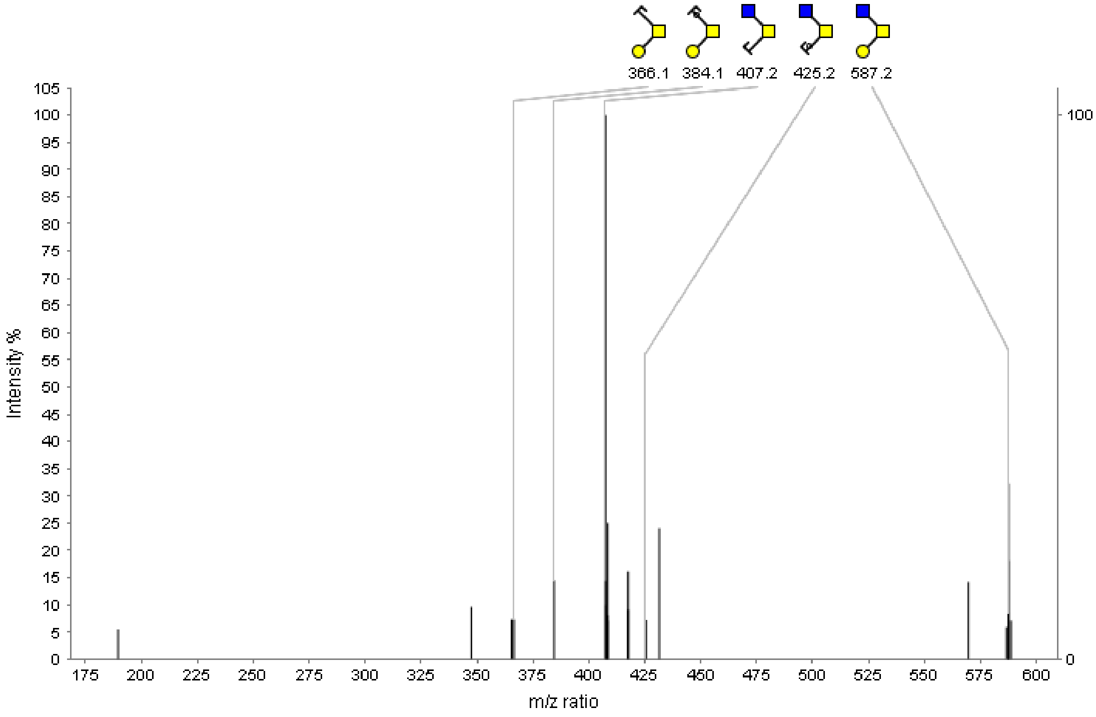

m/z: 587.2
RT: 13.8 min
^{TWIMS}CCS: 233 Å
^{TIMS}CCS: 233 Å

Glycan code: 667-1

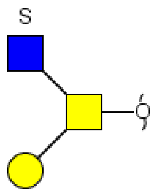

m/z: 667.2
RT: 14.7 min
^{TWIMS}CCS: 226 Å
^{TIMS}CCS: 227 Å

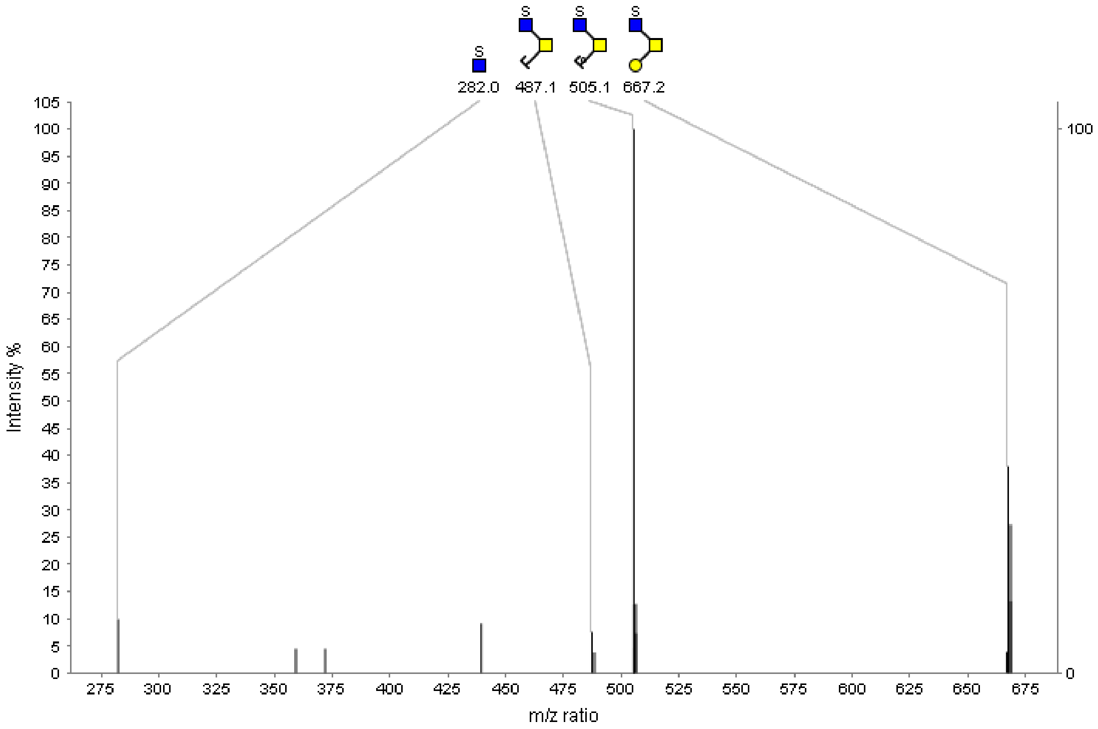

Glycan code: 667-2

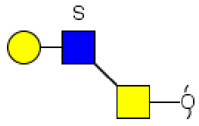

m/z: 667.2
RT: 15.5 min
^{TWIMS}CCS: 233 Å
^{TIMS}CCS: 235 Å

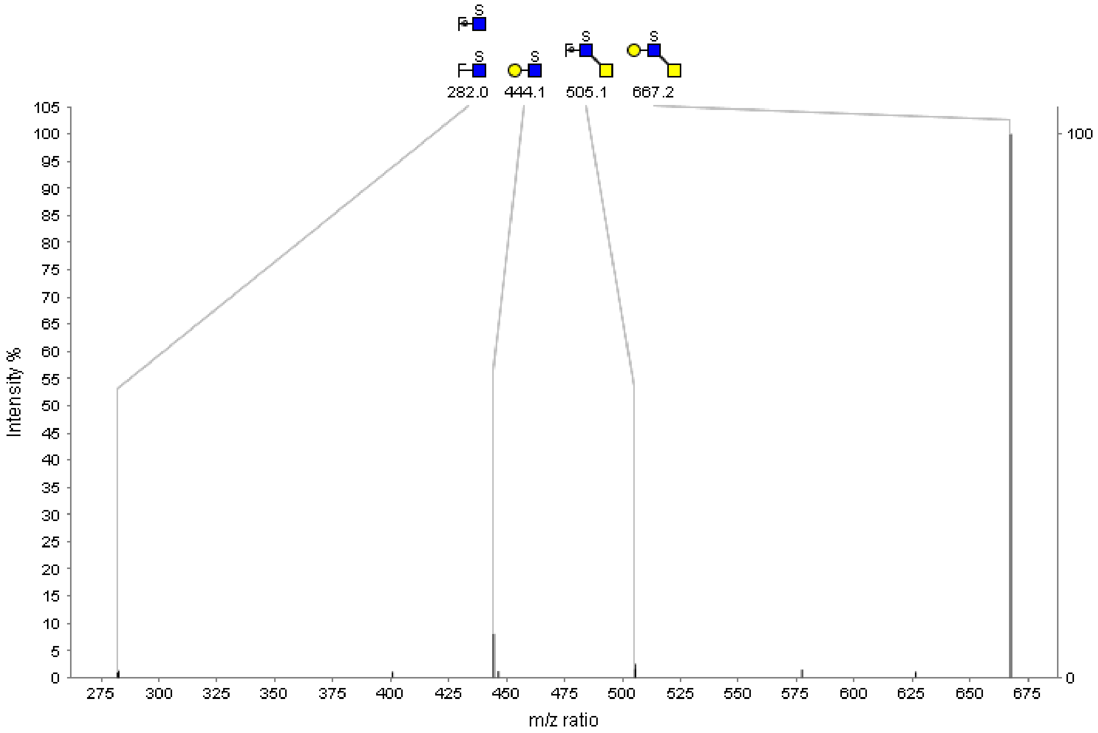

Glycan code: 675-1

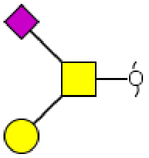

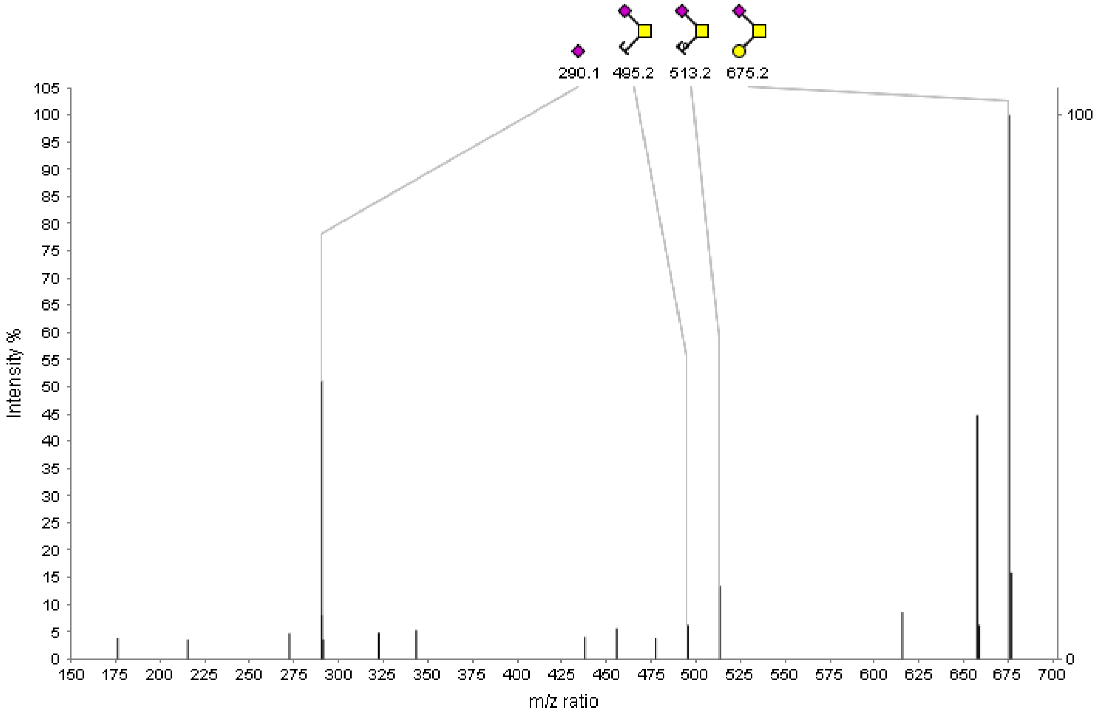

m/z: 675.2
RT: 13.3 min
^{TWIMS}CCS: 245 Å
^{TIMS}CCS: 246 Å

Glycan code: 675-2

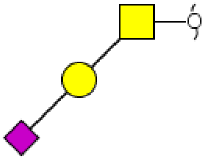

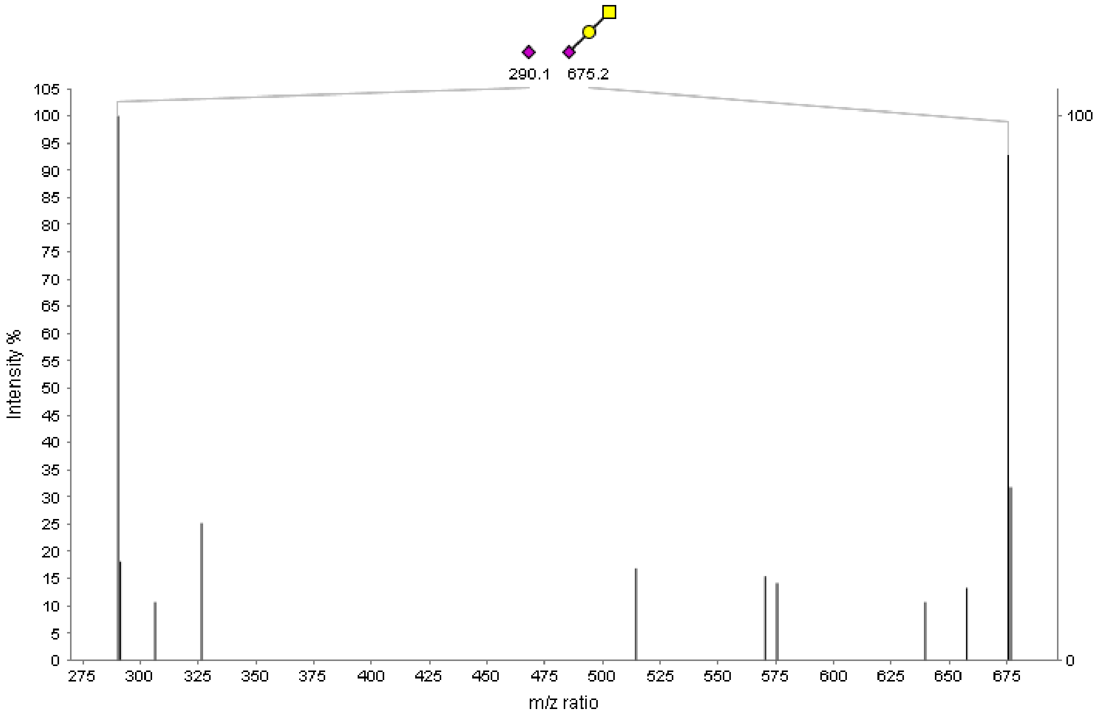

m/z: 675.2
RT: 15.1 min
^{TWIMS}CCS: 249 Å
^{TIMS}CCS: 249 Å

Glycan code: 733-1

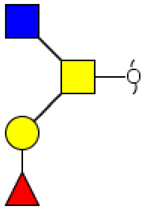

m/z: 733.3
RT: 25.9 min
^{TWIMS}CCS: 245 Å
^{TIMS}CCS: 247 Å

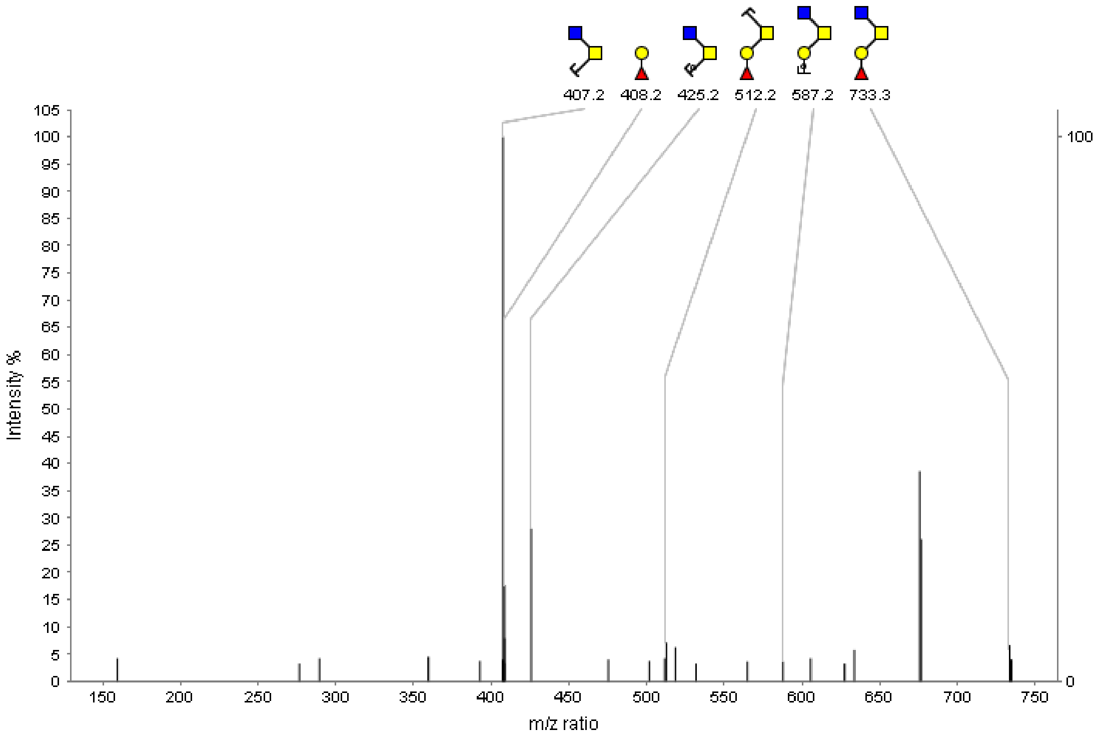

Glycan code: 733-1

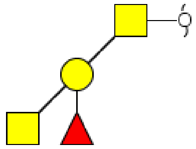

m/z: 733.3
RT: 17.9 min
^{TWIMS}CCS: 255 Å
^{TIMS}CCS: 256 Å

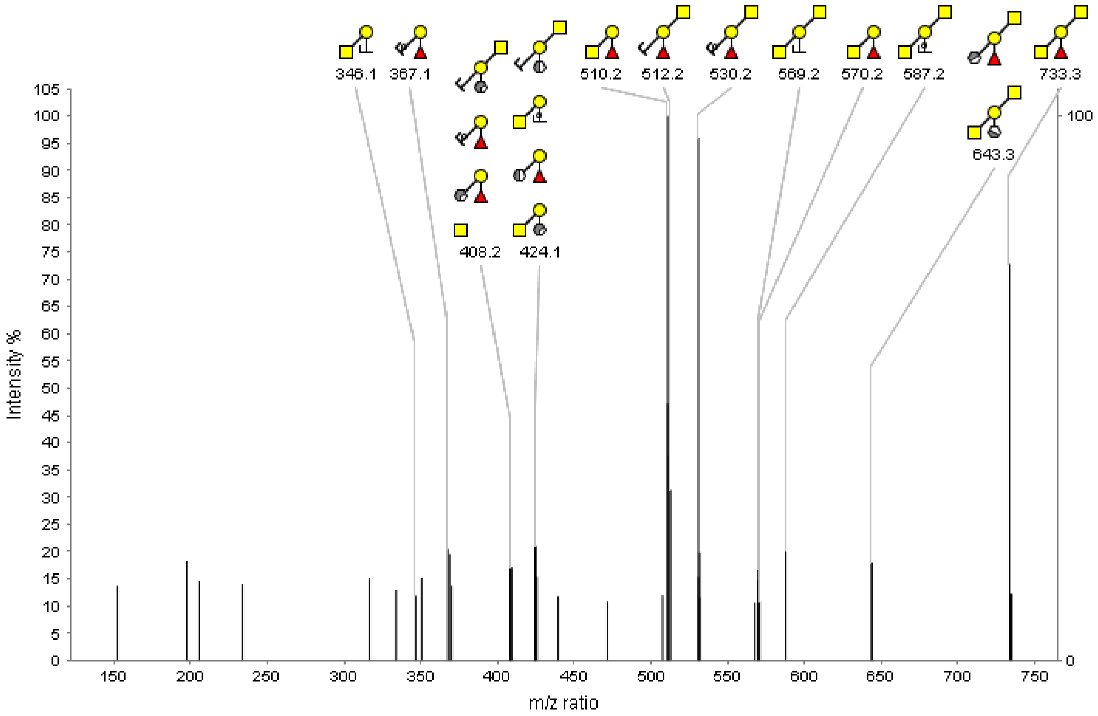

Glycan code: 749-1

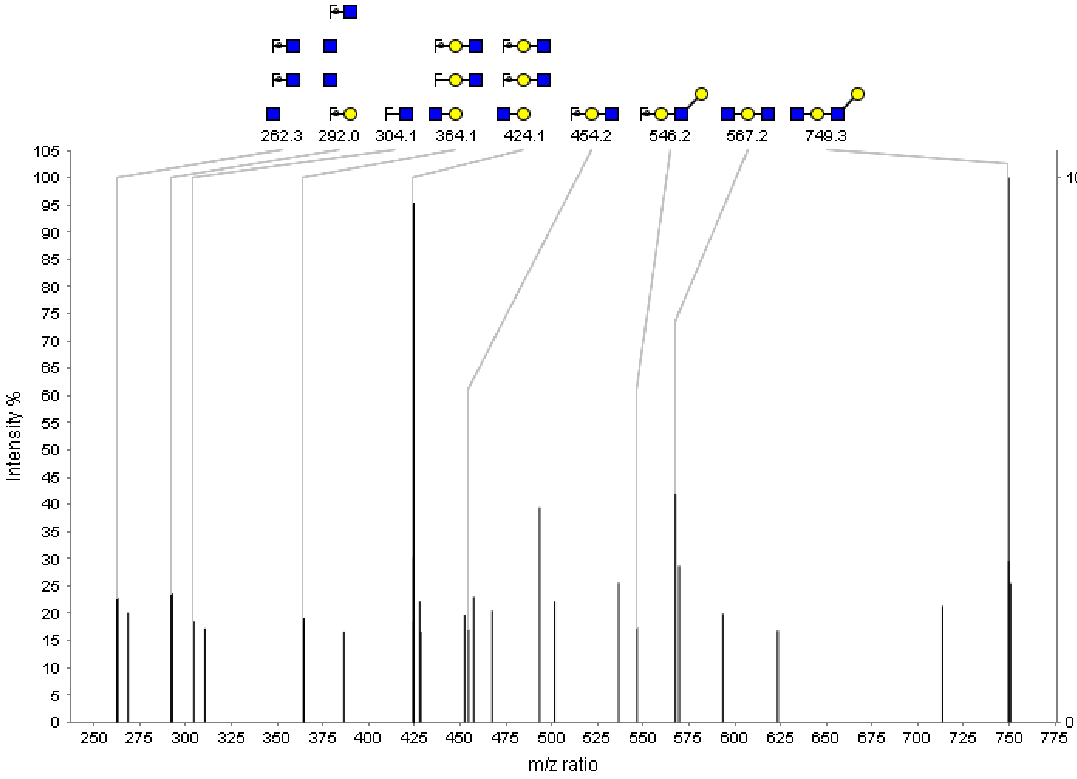

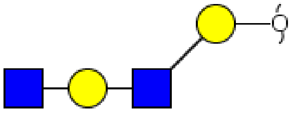

m/z: 749.3
RT: 15.9 min
TWIMSCCS: 244 Å
TIMSCCS: 245 Å

Glycan code: 749-2

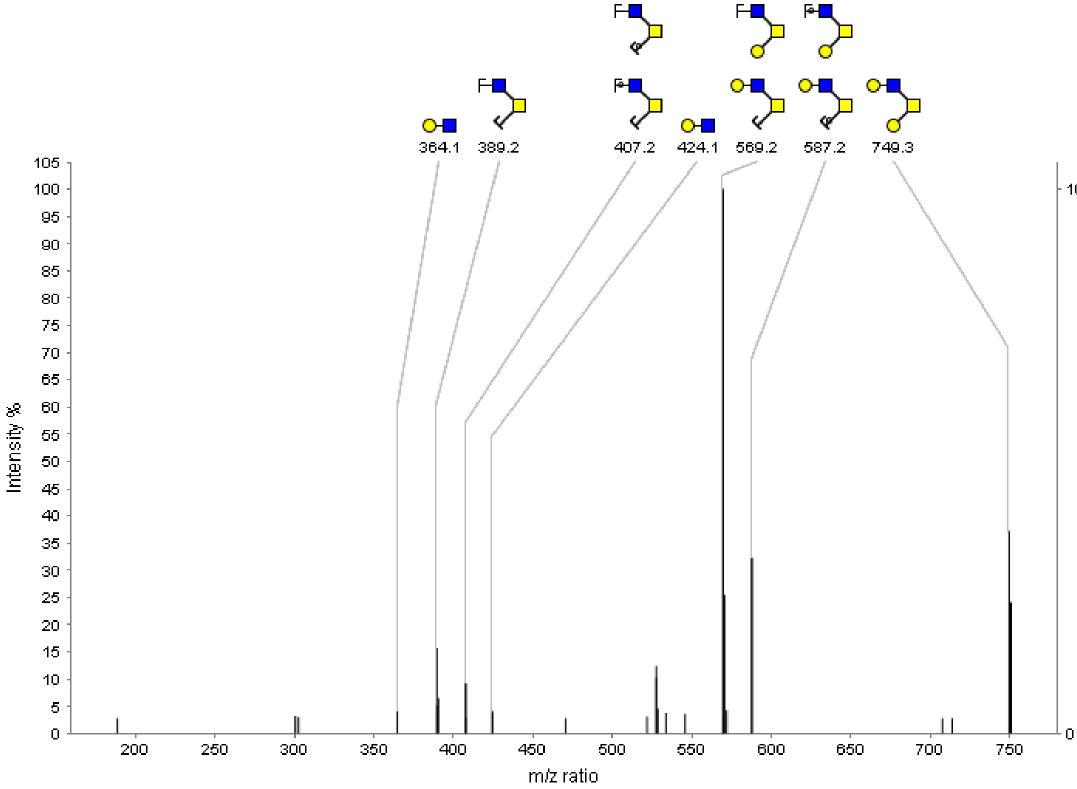

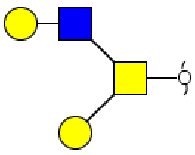

m/z: 749.3
RT: 16.4 min
TWIMSCCS: 246 Å
TIMSCCS: 247 Å

Glycan code: 749-3

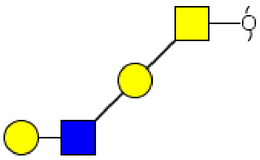

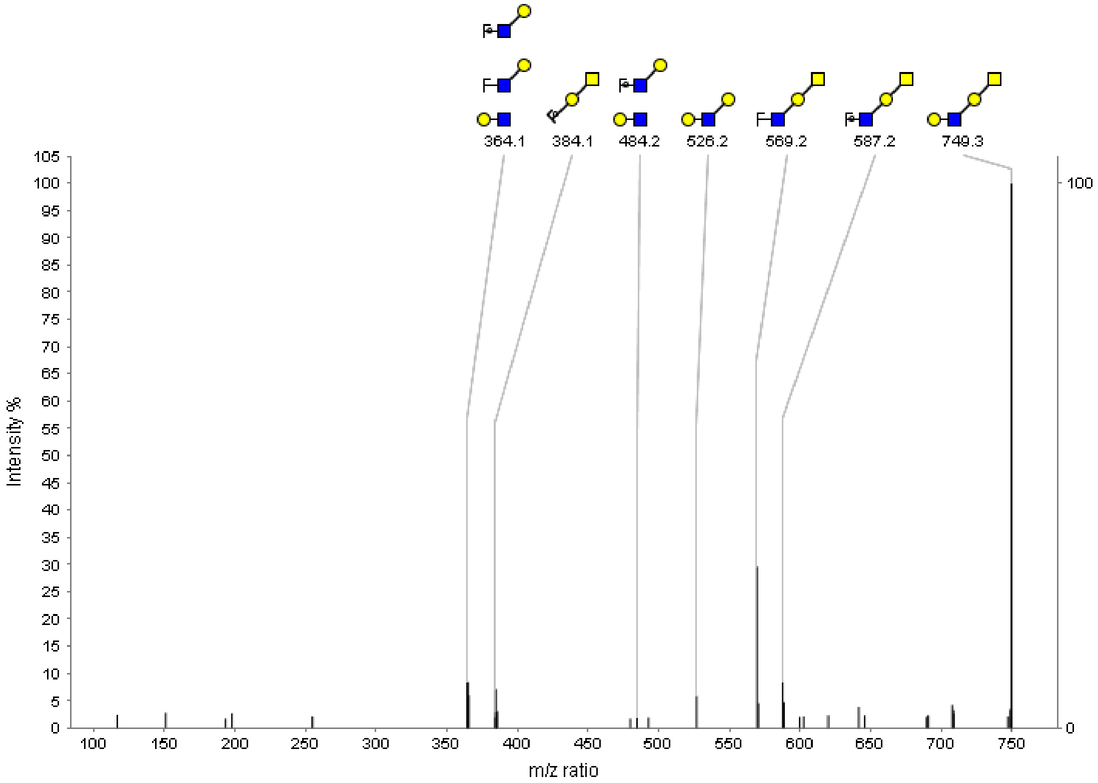

m/z: 749.3
RT: 17.0 min
^{TWIMS}CCS: 279 Å
^{TIMS}CCS: 280 Å

Glycan code: 790-1

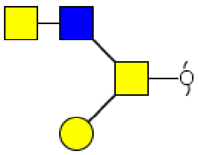

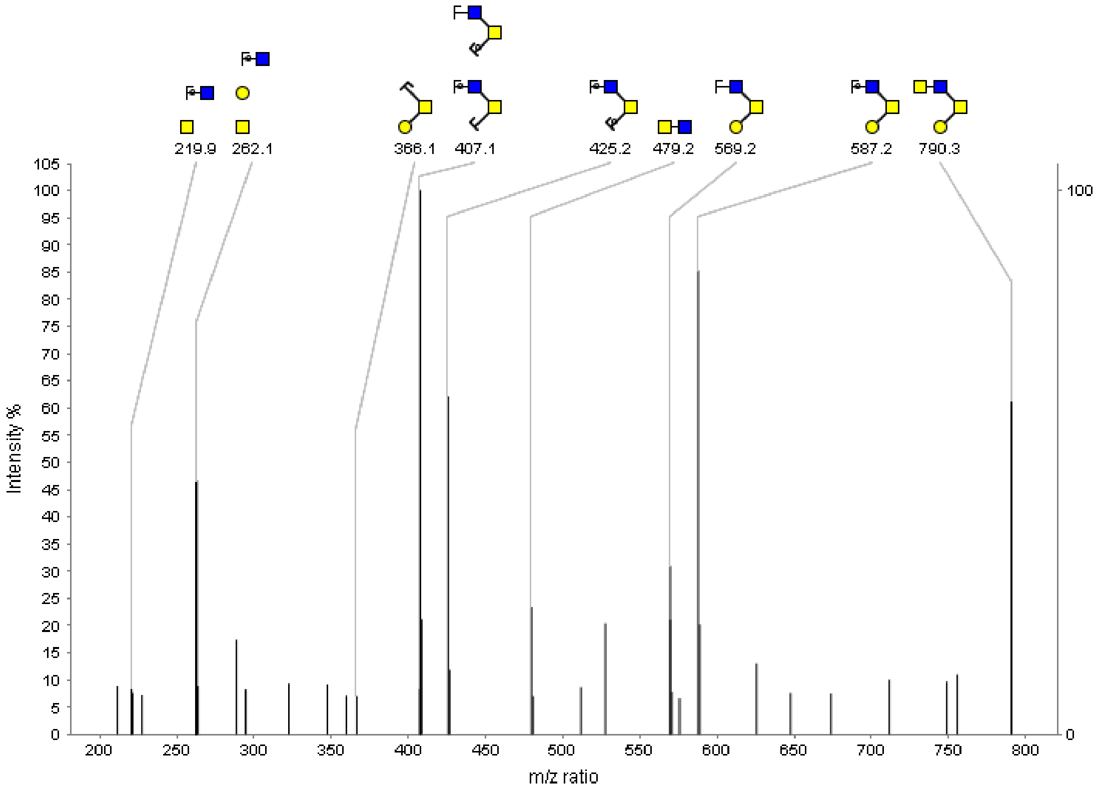

m/z: 790.3
RT: 16.2 min
^{TWIMS}CCS: 253 Å
^{TIMS}CCS: 253 Å

Glycan code: 790-2

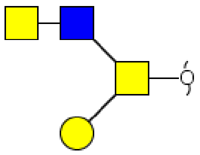

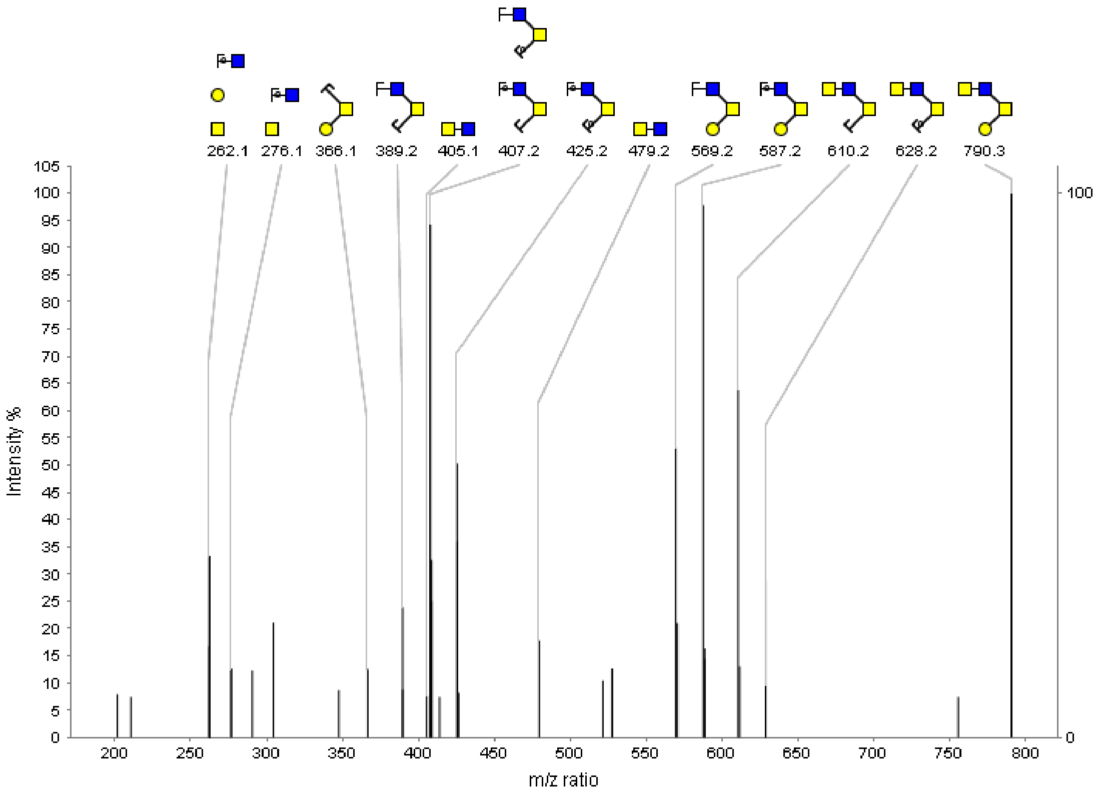

m/z: 790.3
RT: 16.2 min
^{TWIMS}CCS: 265 Å
^{TIMS}CCS: 266 Å

Glycan code: 790-3

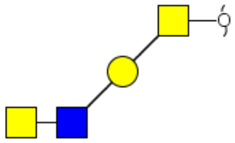

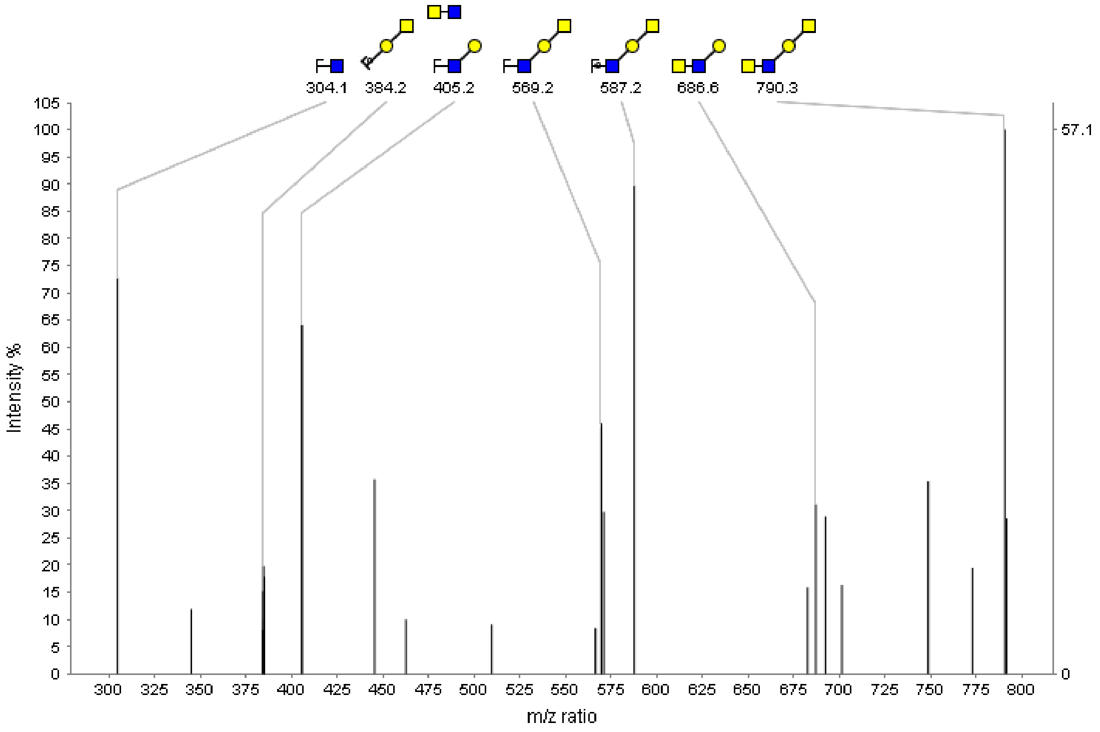

m/z: 790.3
RT: 16.2 min
^{TWIMS}CCS: 265 Å
^{TIMS}CCS: 266 Å

Glycan code: 813-1

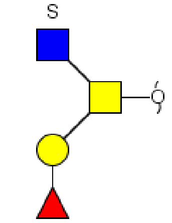

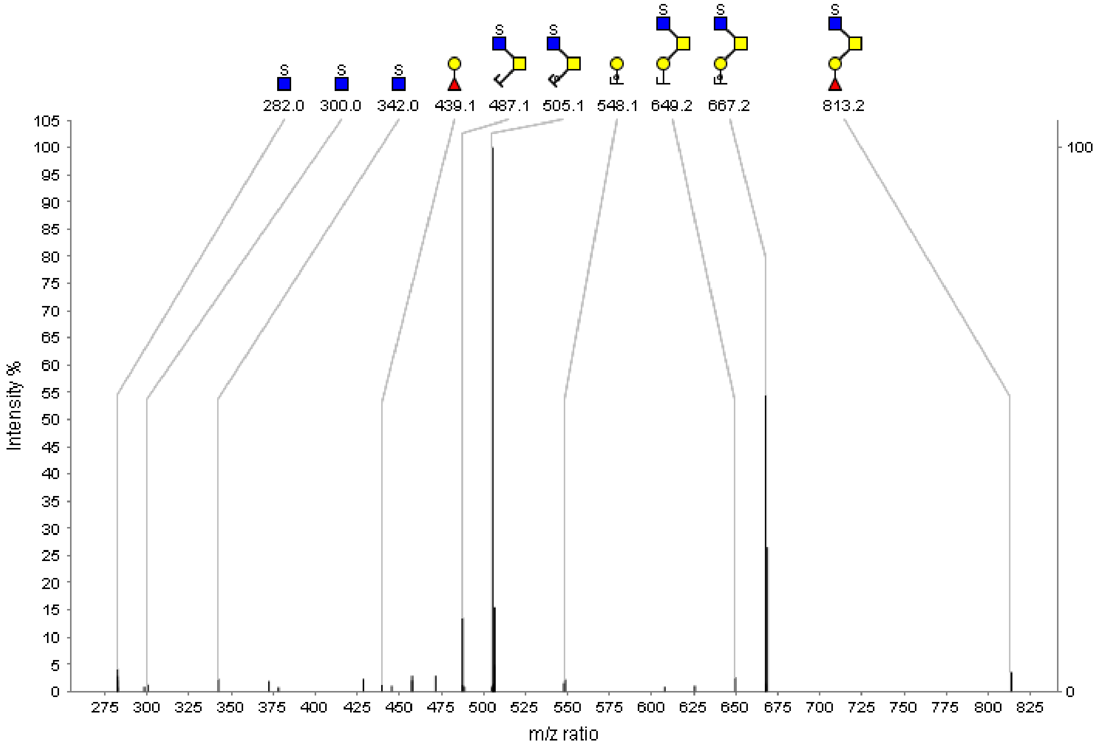

m/z: 813.2
RT: 26.0 min
^{TWIMS}CCS: 254 Å
^{TIMS}CCS: 255 Å

Glycan code: 878-1

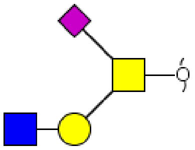

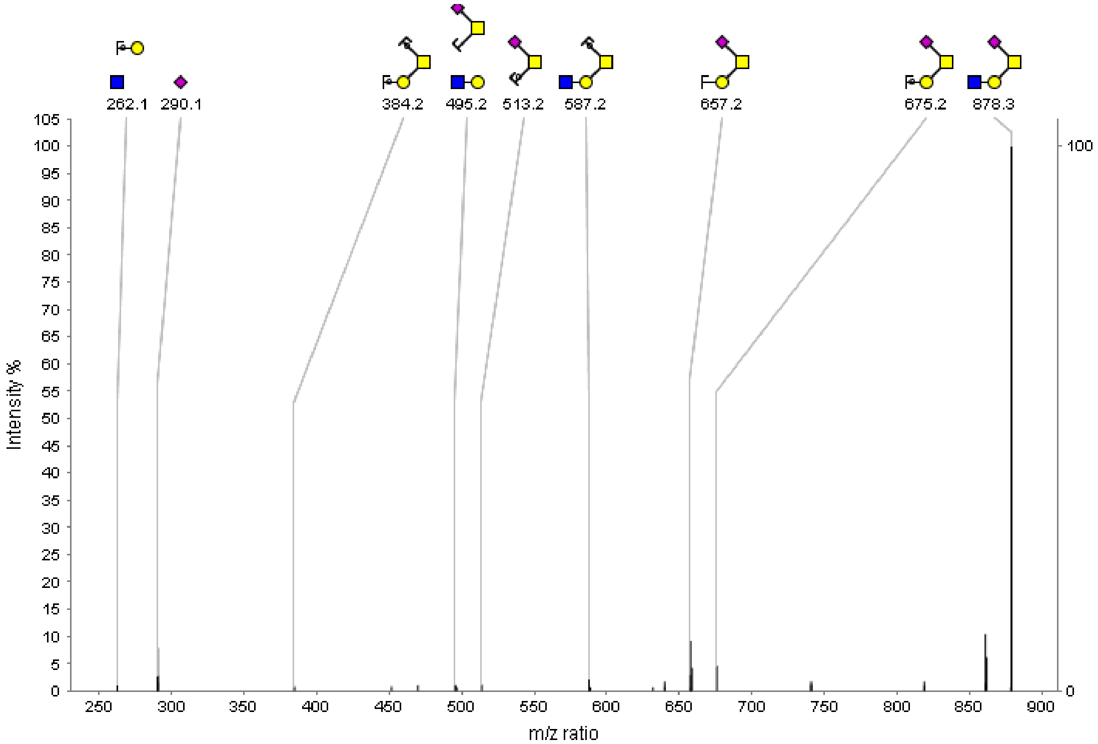

m/z: 878.3
RT: 15.7 min
^{TWIMS}CCS: 274 Å
^{TIMS}CCS: 274 Å

Glycan code: 895-1

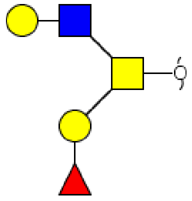

m/z: 895.3
RT: 26.0 min
^{TWIMS}CCS: 273 Å
^{TIMS}CCS: 273 Å

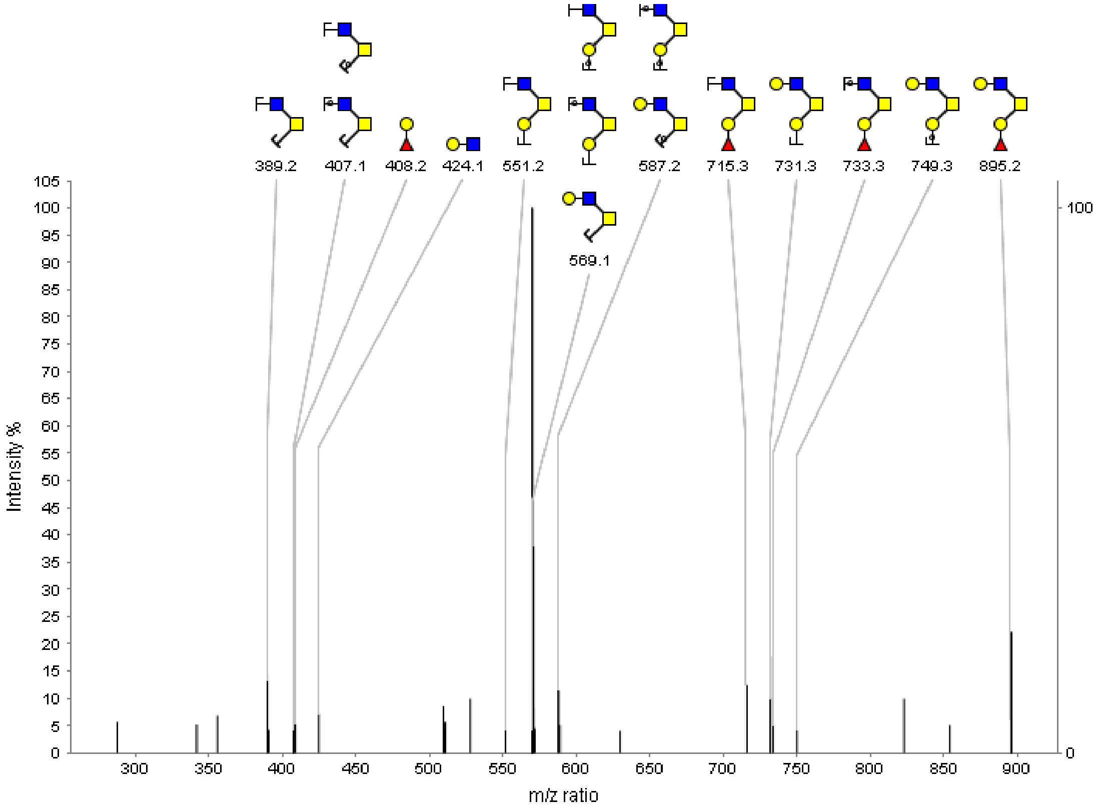

Glycan code: 895-2

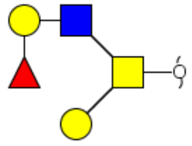

m/z: 895.3
RT: 20.7 min
^{TWIMS}CCS: 276 Å
^{TIMS}CCS: 275 Å

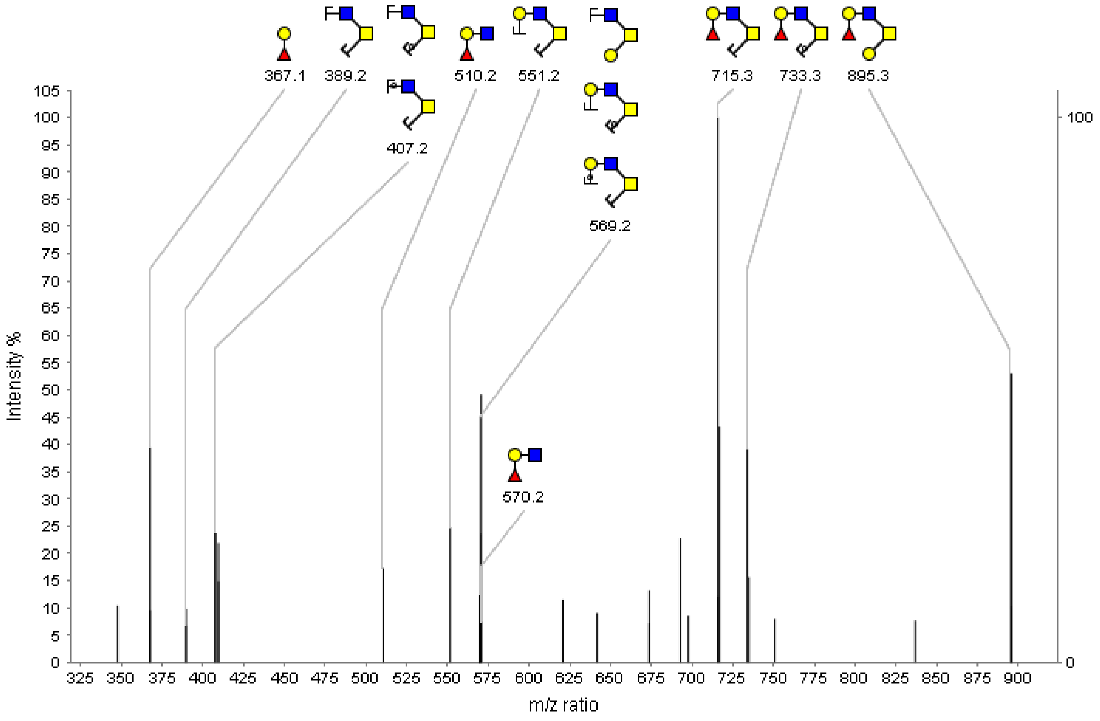

Glycan code: 895-3

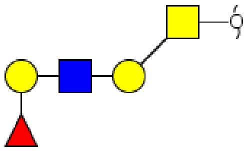

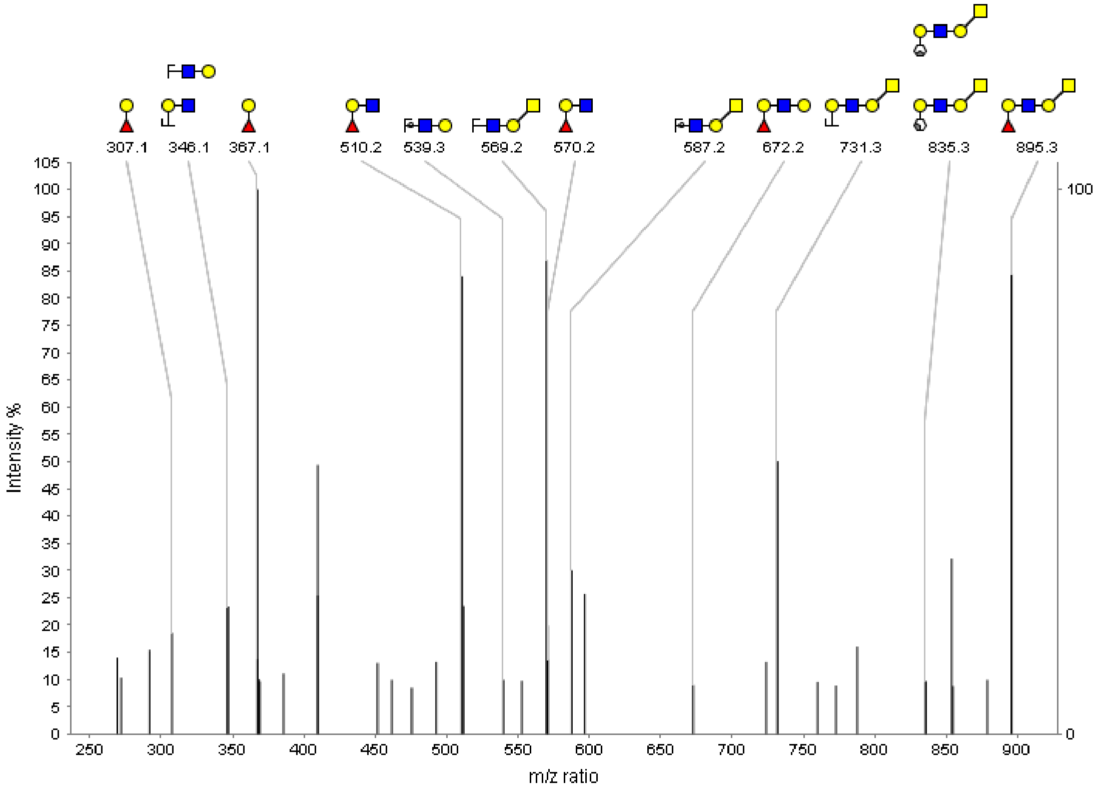

m/z: 895.3
RT: 19.7 min
^{TWIMS}CCS: 290 Å
^{TIMS}CCS: 290 Å

Glycan code: 895-4

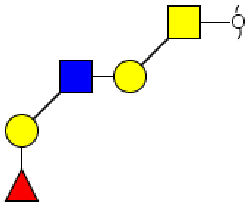

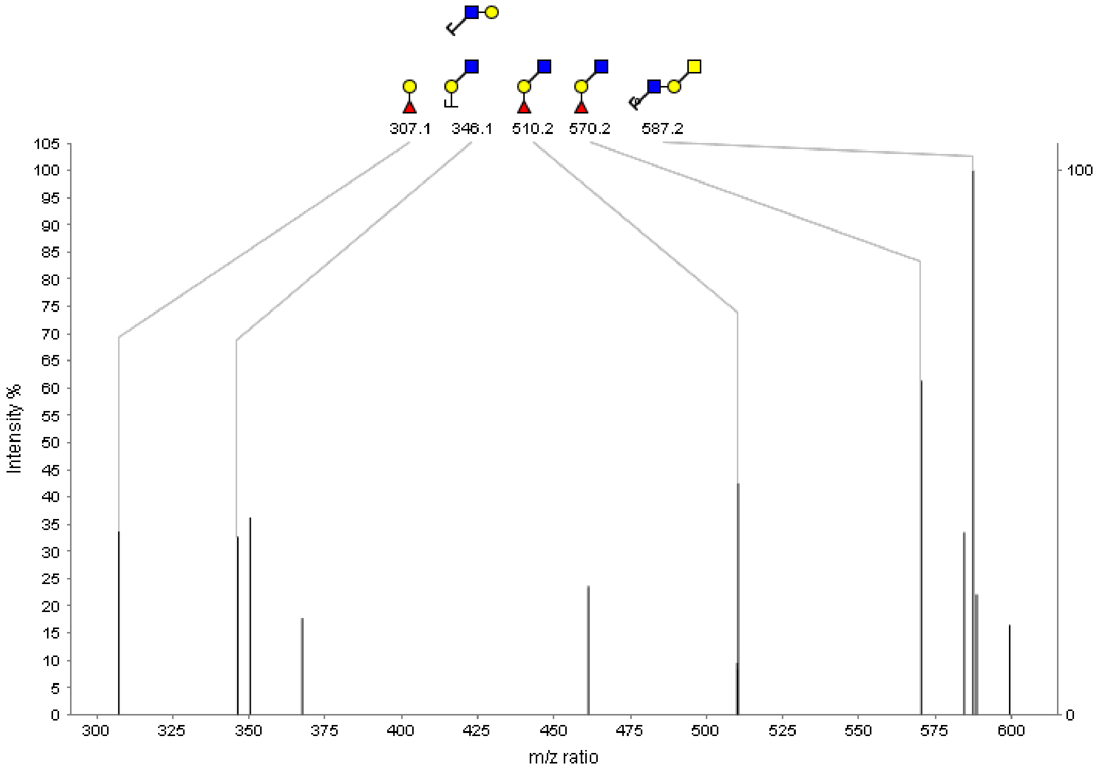

m/z: 895.3
RT: 18.0 min
^{TWIMS}CCS: 298 Å
^{TIMS}CCS: 297 Å

Glycan code: 936-1

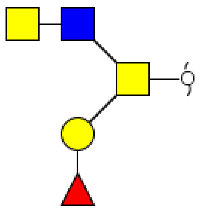

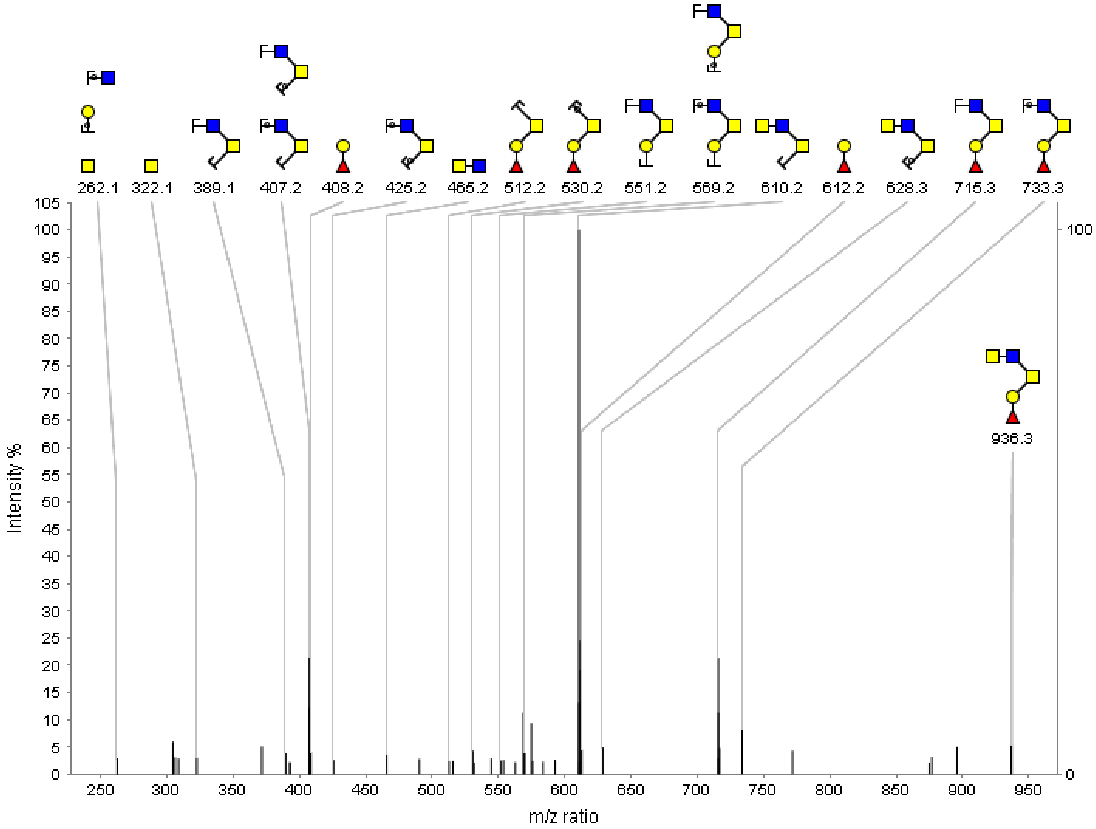

m/z: 936.4
RT: 26.0 min
^{TWIMS}CCS: 285 Å
^{TIMS}CCS: 286 Å

Glycan code: 936-2

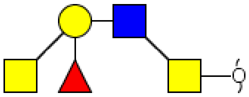

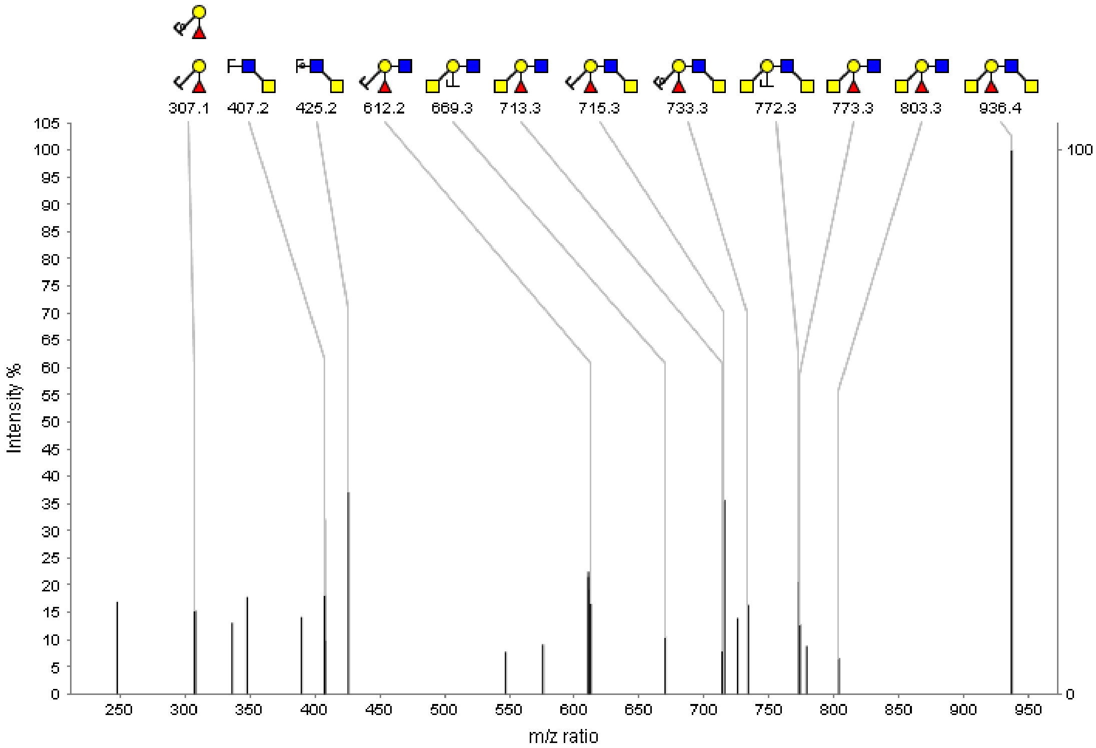

m/z: 936.4
RT: 18.6 min
^{TWIMS}CCS: 287 Å
^{TIMS}CCS: 289 Å

Glycan code: 952-1

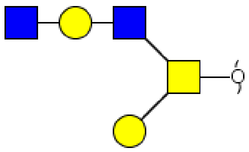

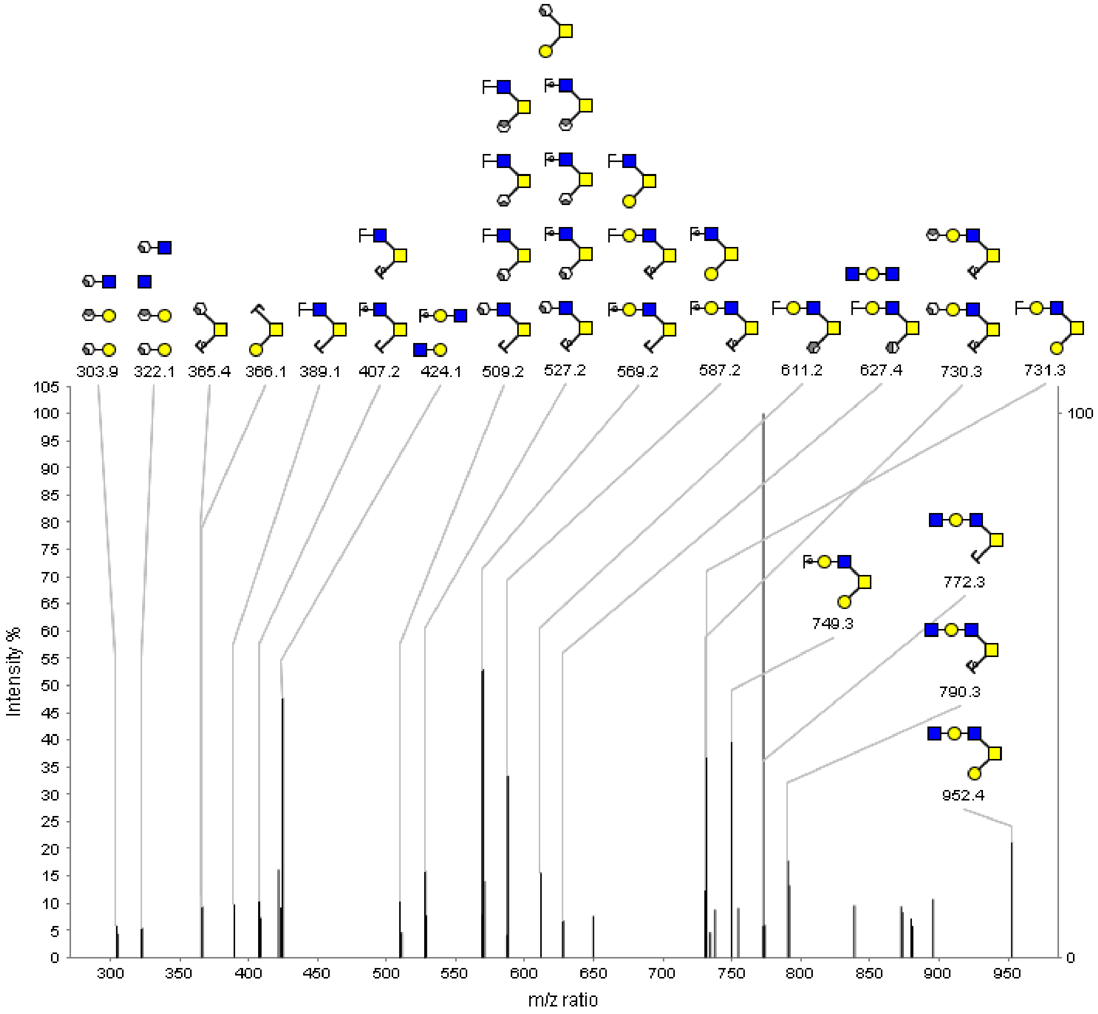

m/z: 952.4
RT: 17.8 min
^{TWIMS}CCS: 276 Å
^{TIMS}CCS: 280 Å

Glycan code: 952-2

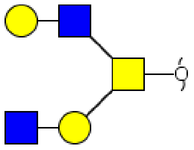

m/z: 952.4
RT: 17.8 min
^{TWIMS}CCS: 282 Å
^{TIMS}CCS: 285 Å

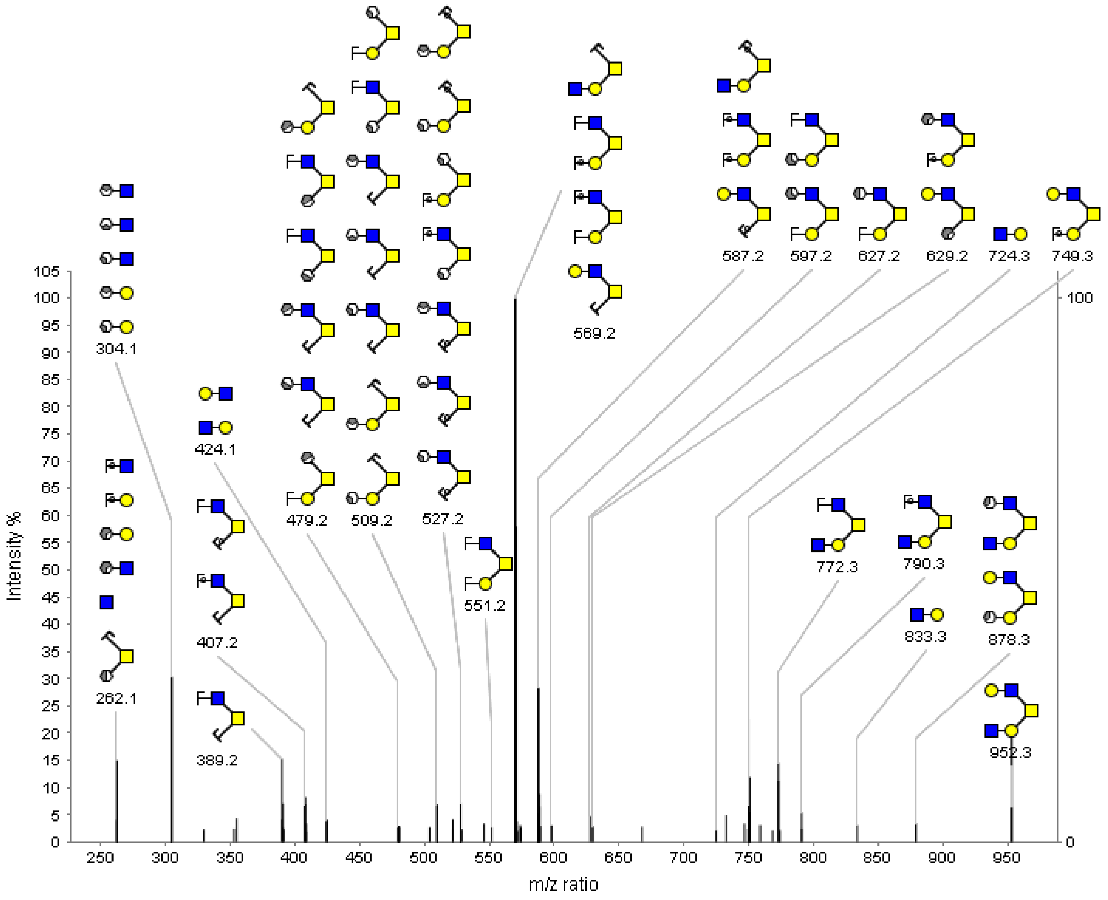

Glycan code: 952-3

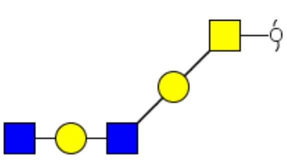

m/z: 952.4
RT: 17.4 min
^{TWIMS}CCS: 295 Å
^{TIMS}CCS: 295 Å

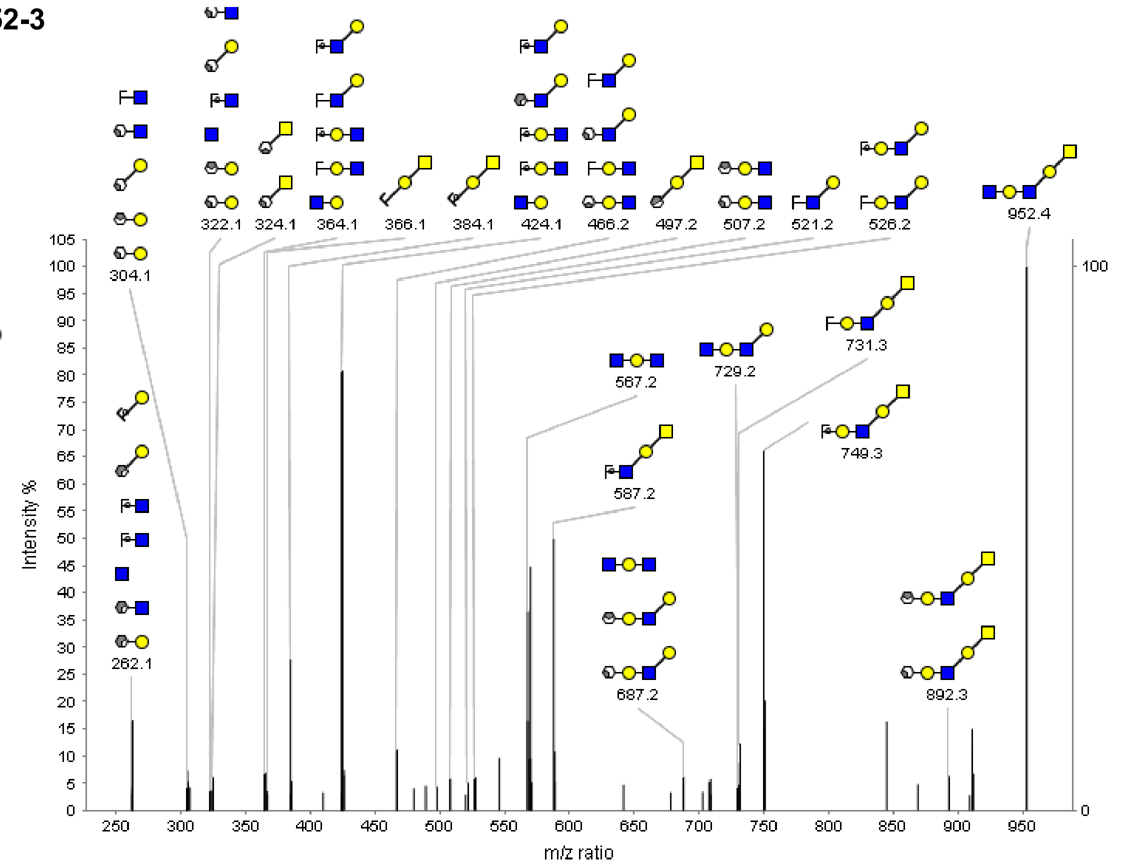

Glycan code: 975-1

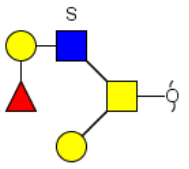

m/z: 975.3
^{TWIMS}CCS: n.d.
^{TIMS}CCS: 287 Å

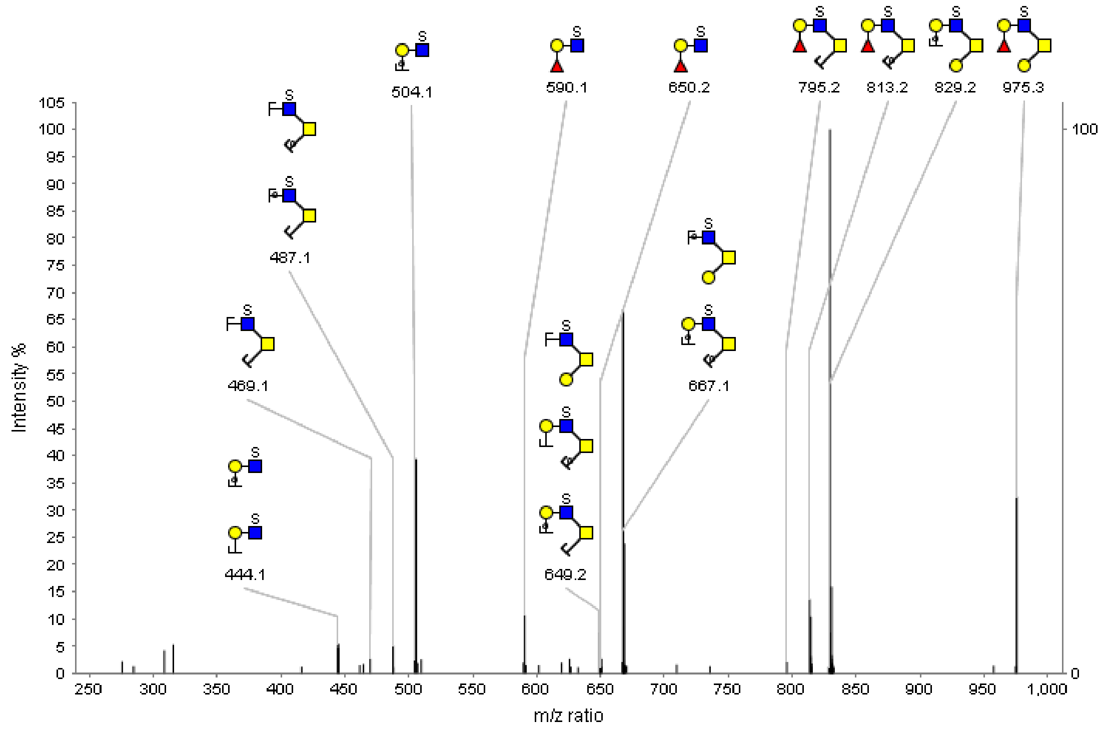

Glycan code: 975-2

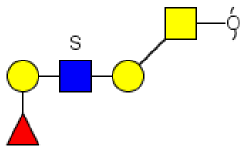

m/z: 952.4
RT: 17.3 min
^{TWIMS}CCS: 303 Å
^{TIMS}CCS: 302 Å

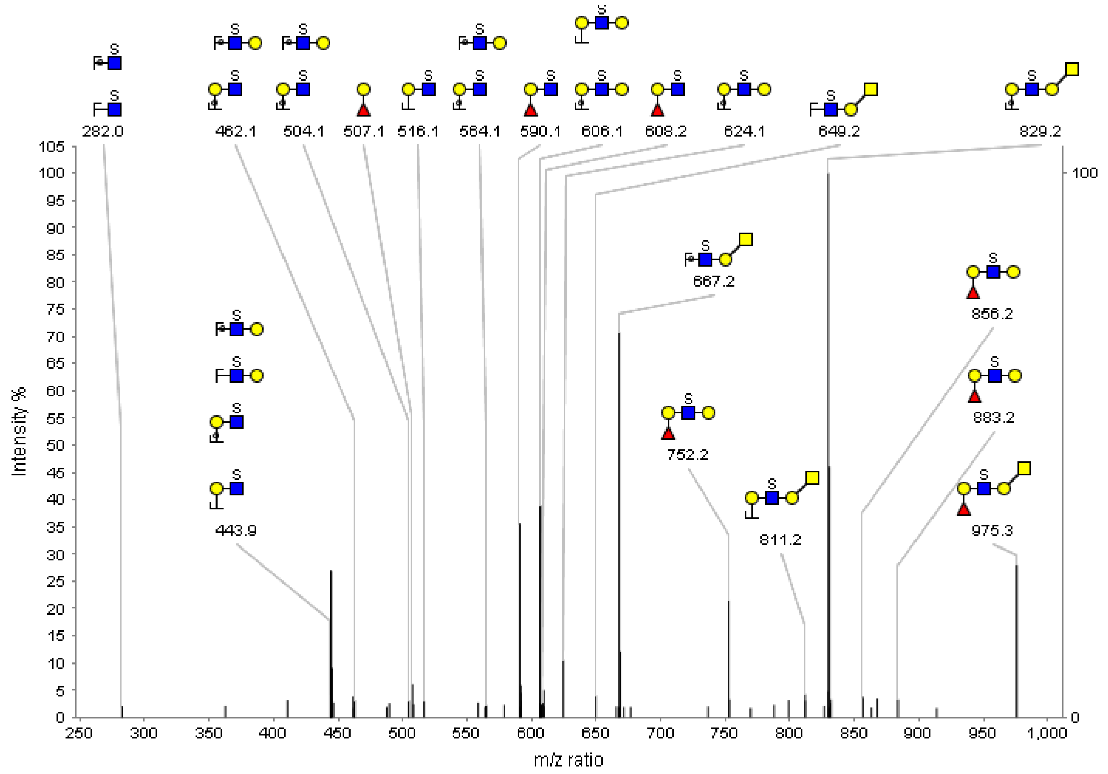

Glycan code: 993-1

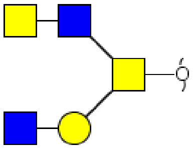

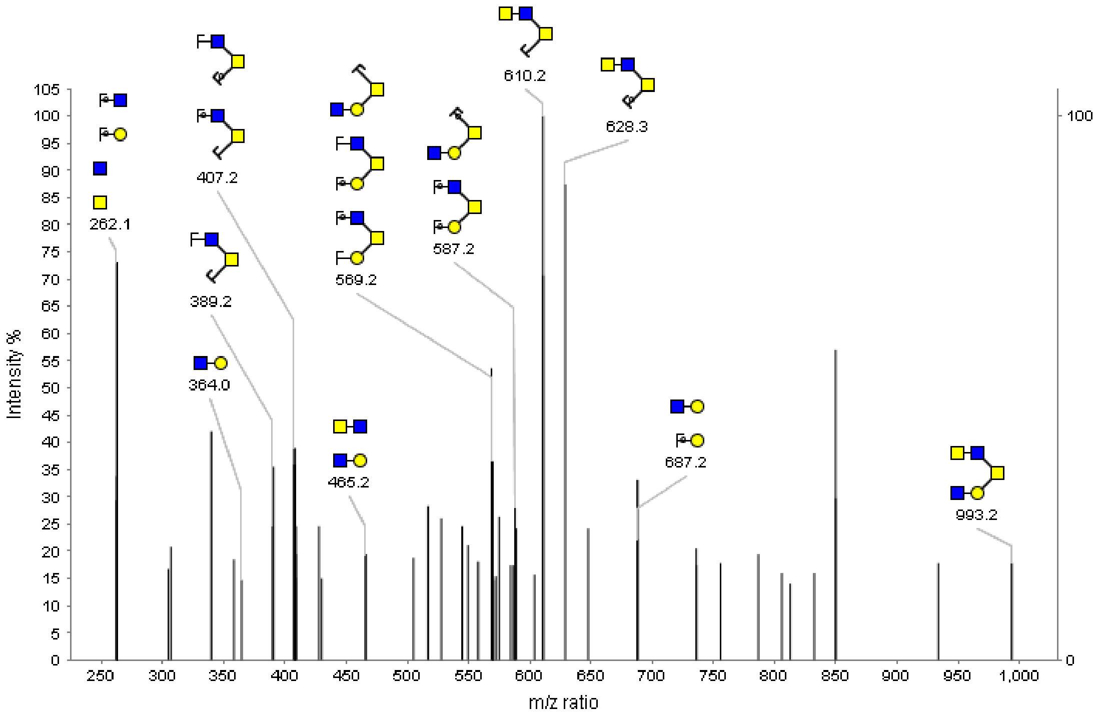

m/z: 993.4
RT: 17.2 min
^{TWIMS}CCS: 295 Å
^{TIMS}CCS: 295 Å

Glycan code: 1040-1

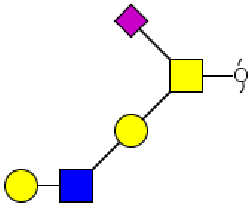

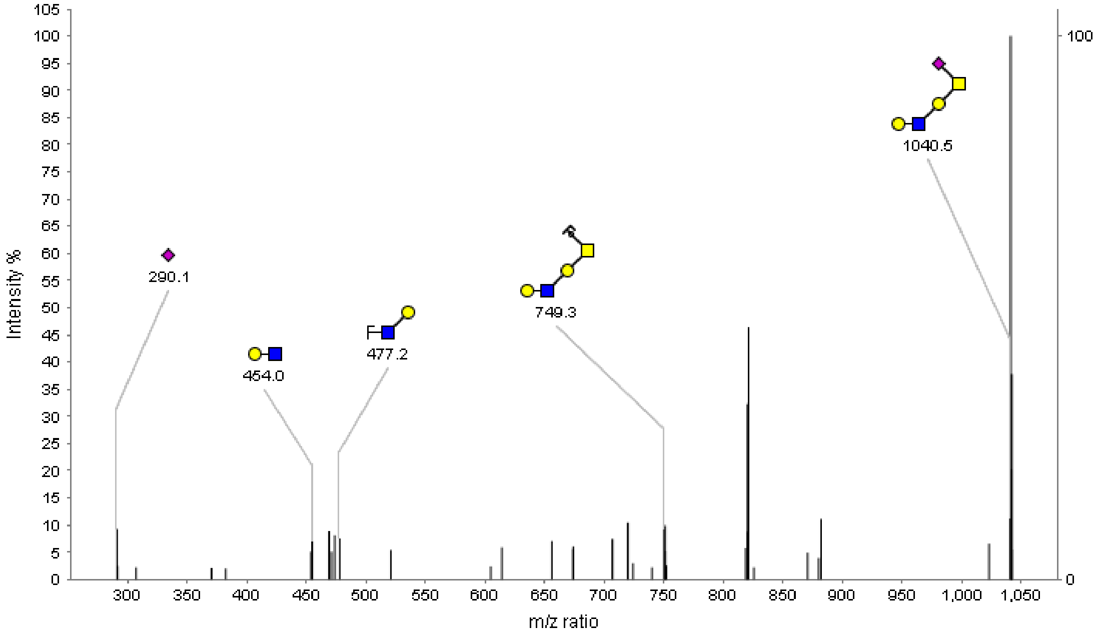

m/z: 1040.4
RT: 17.7 min
^{TWIMS}CCS: 292 Å
^{TIMS}CCS: 291 Å

Glycan code: 1041-1

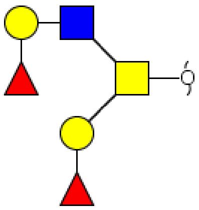

m/z: 1041.4
RT: 27.3 min
TWIMSCCS: 299 Å
TIMSCCS: 298 Å

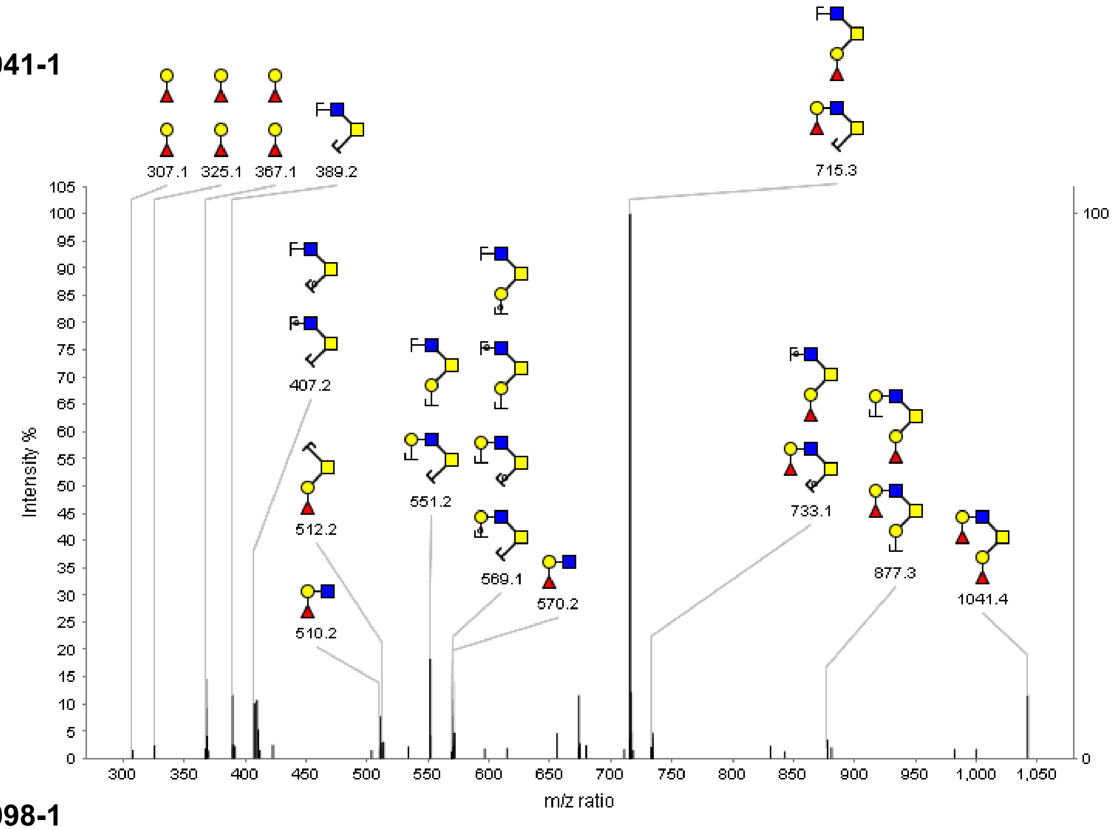

Glycan code: 1098-1

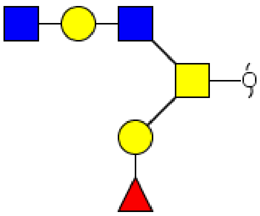

m/z: 1098.4
RT: 26.3 min
TWIMSCCS: 309 Å
TIMSCCS: 307 Å

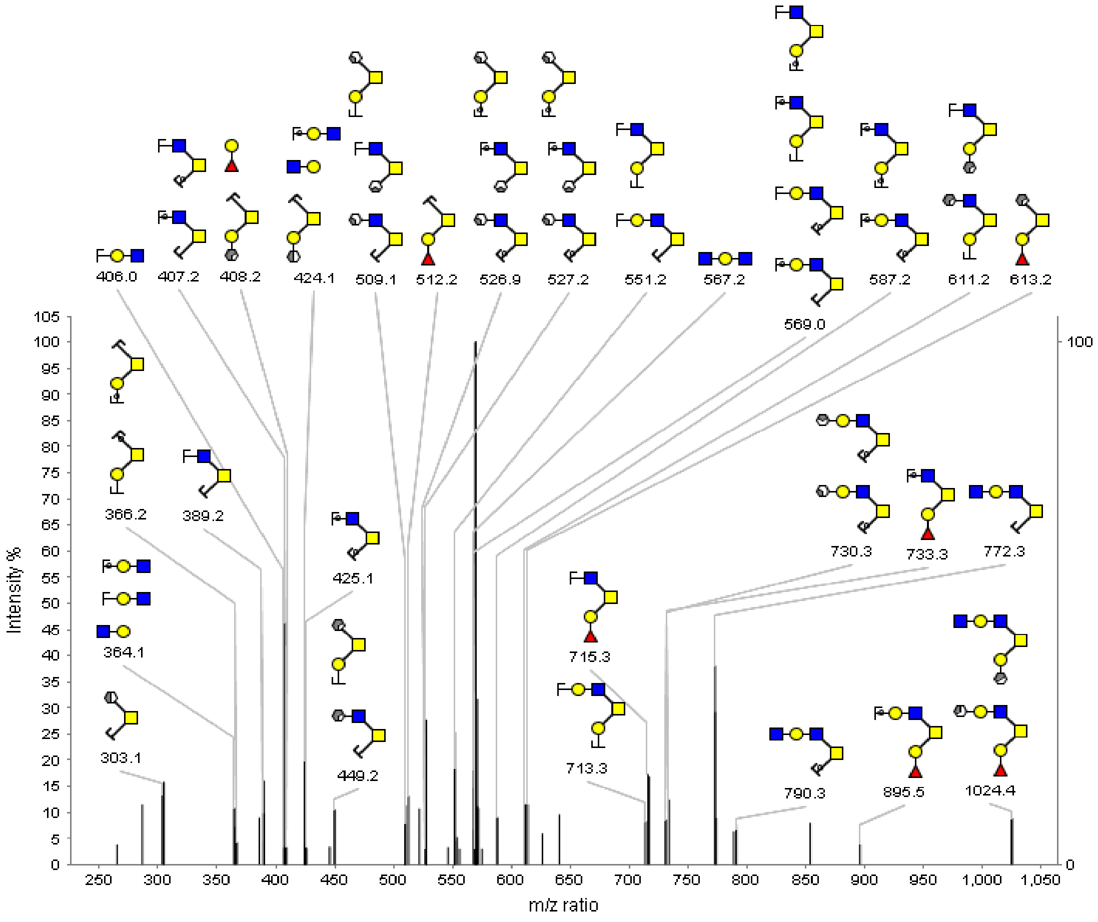

Glycan code: 1098-2

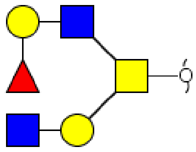

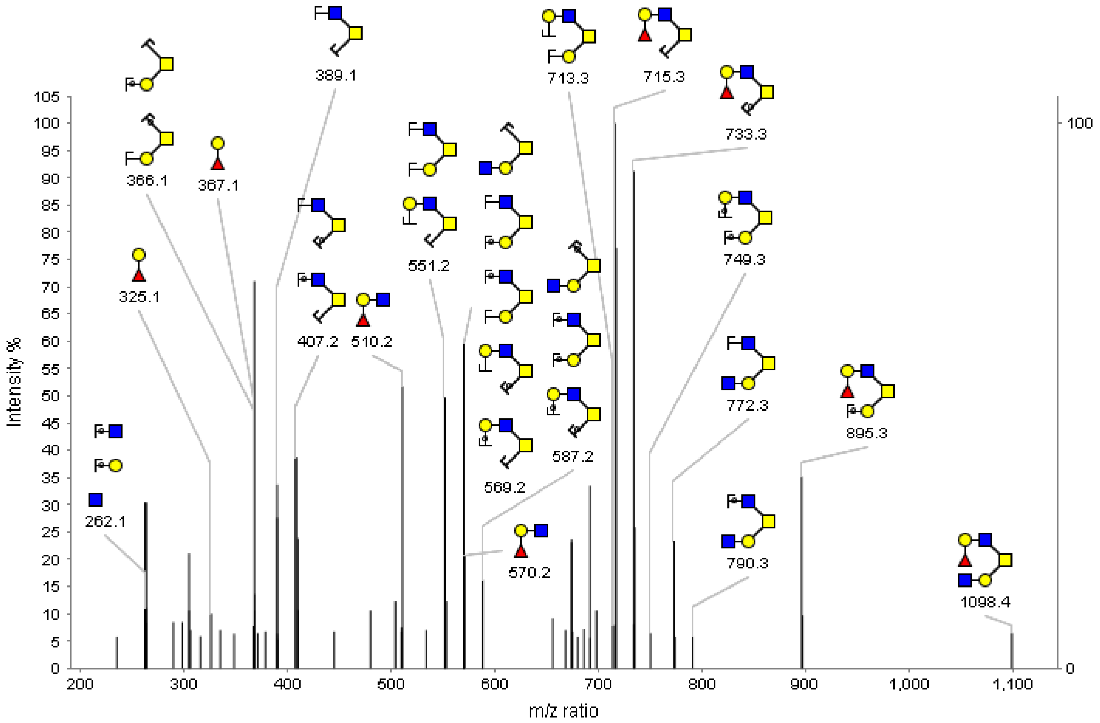

m/z: 1098.4
RT: 21.8 min
TWIMSCCS: 311 Å
TIMSCCS: 310 Å

Glycan code: 1098-3

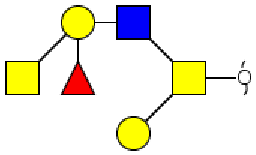

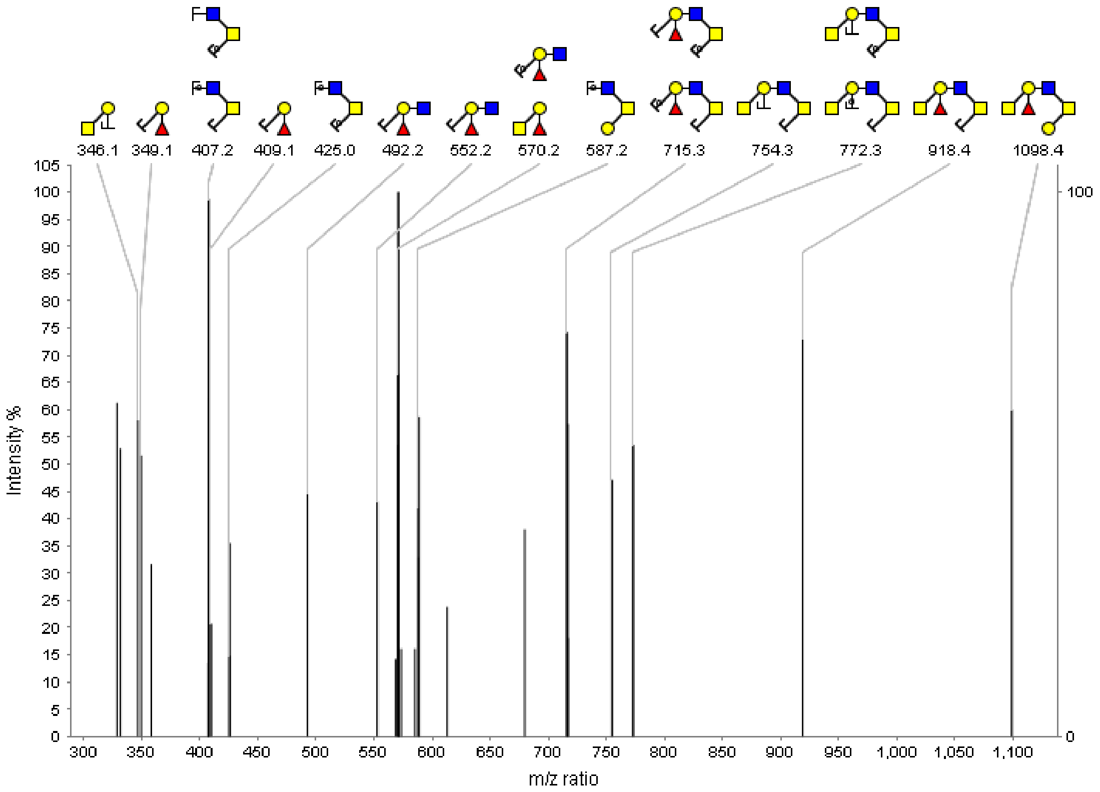

m/z: 1098.4
RT: 16.0 min
TWIMSCCS: 317 Å
TIMSCCS: 315 Å

Glycan code: 1098-4

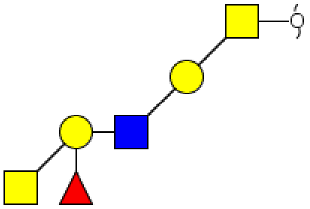

m/z: 1098.4
RT: 15.7 min
^{TWIMS}CCS: 326 Å
^{TIMS}CCS: 326 Å

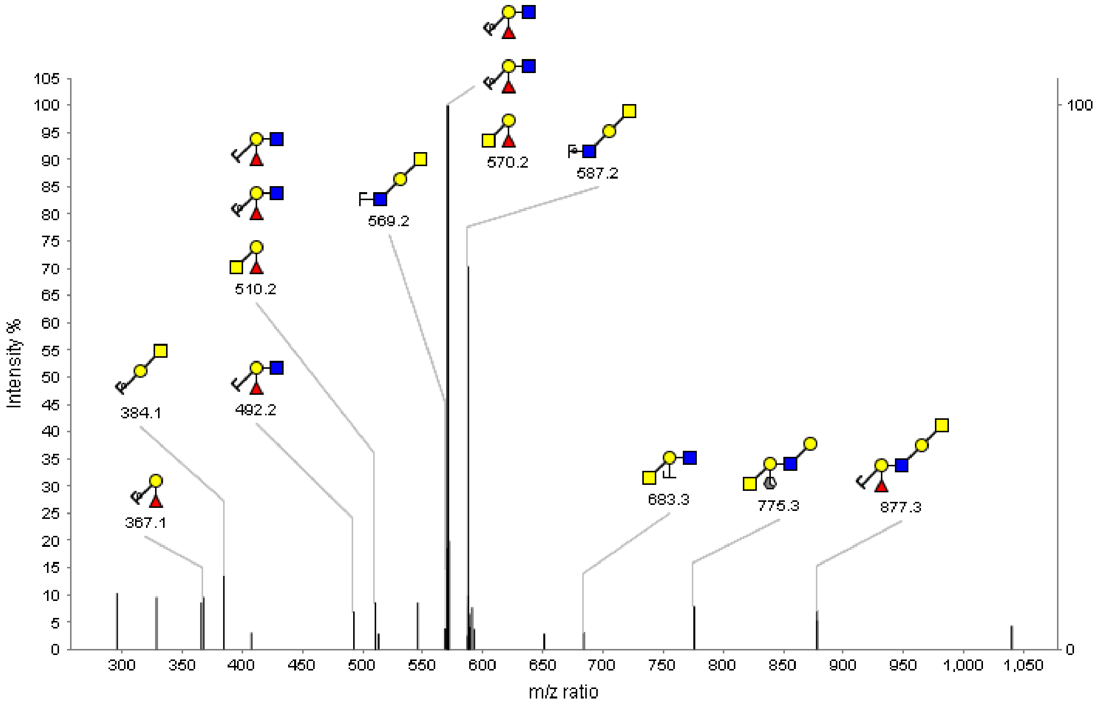

Glycan code: 1155-1

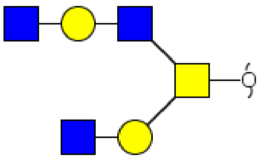

m/z: 1155.4
RT: 18.5 min
^{TWIMS}CCS: 316 Å
^{TIMS}CCS: 317 Å

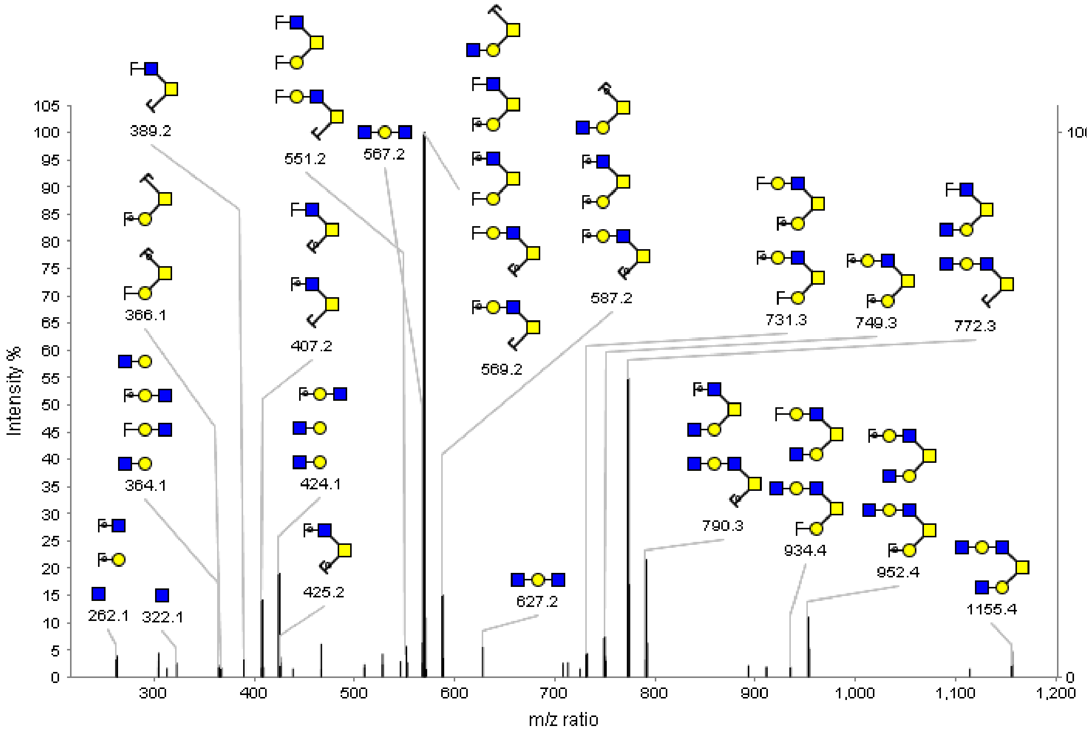

Glycan code: 1178-1

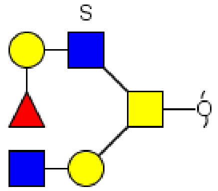

m/z: 1178.4
TWIMSCCS: n.d.
TIMSCCS: 317 Å

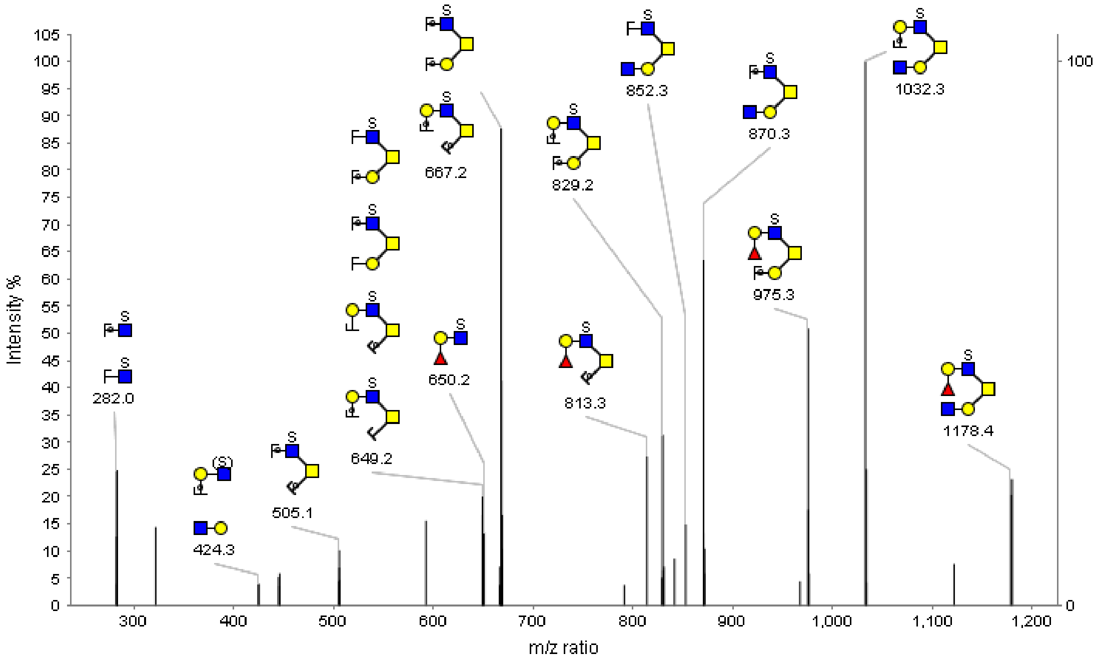

Glycan code: 1178-2

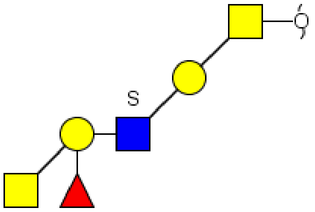

m/z: 1178.4
TWIMSCCS: n.d.
TIMSCCS: 339 Å

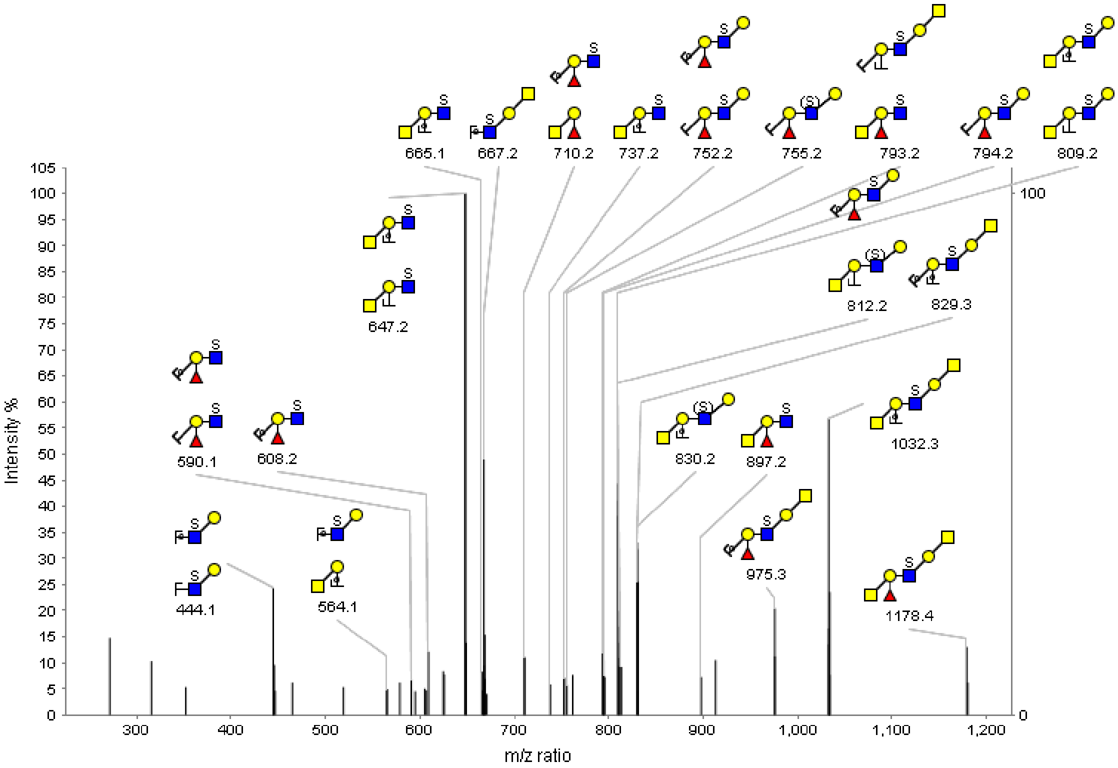

Glycan code: 621-1

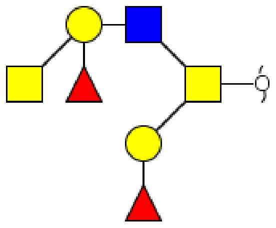

m/z: 621.7
^{TWIMS}CCS: n.d.
^{TIMS}CCS: 349 Å

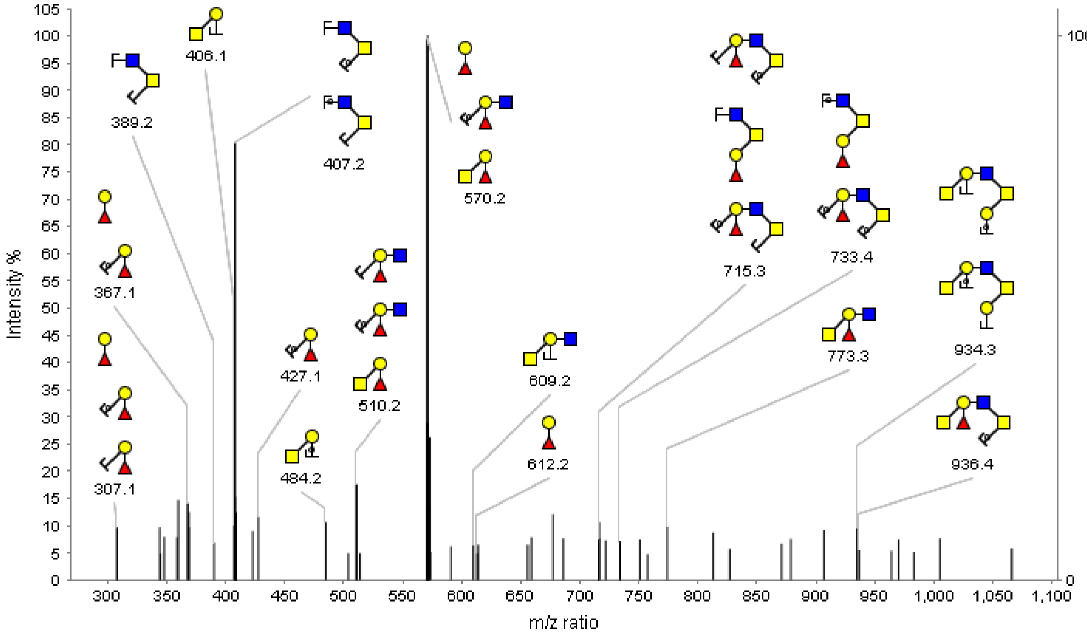

Glycan code: 650-1

m/z range: 150-650

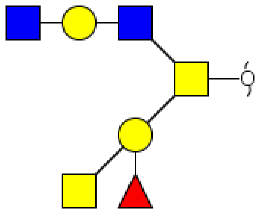

m/z: 650.2
RT: 18.0 min
^{TWIMS}CCS: 364 Å
^{TIMS}CCS: 356 Å

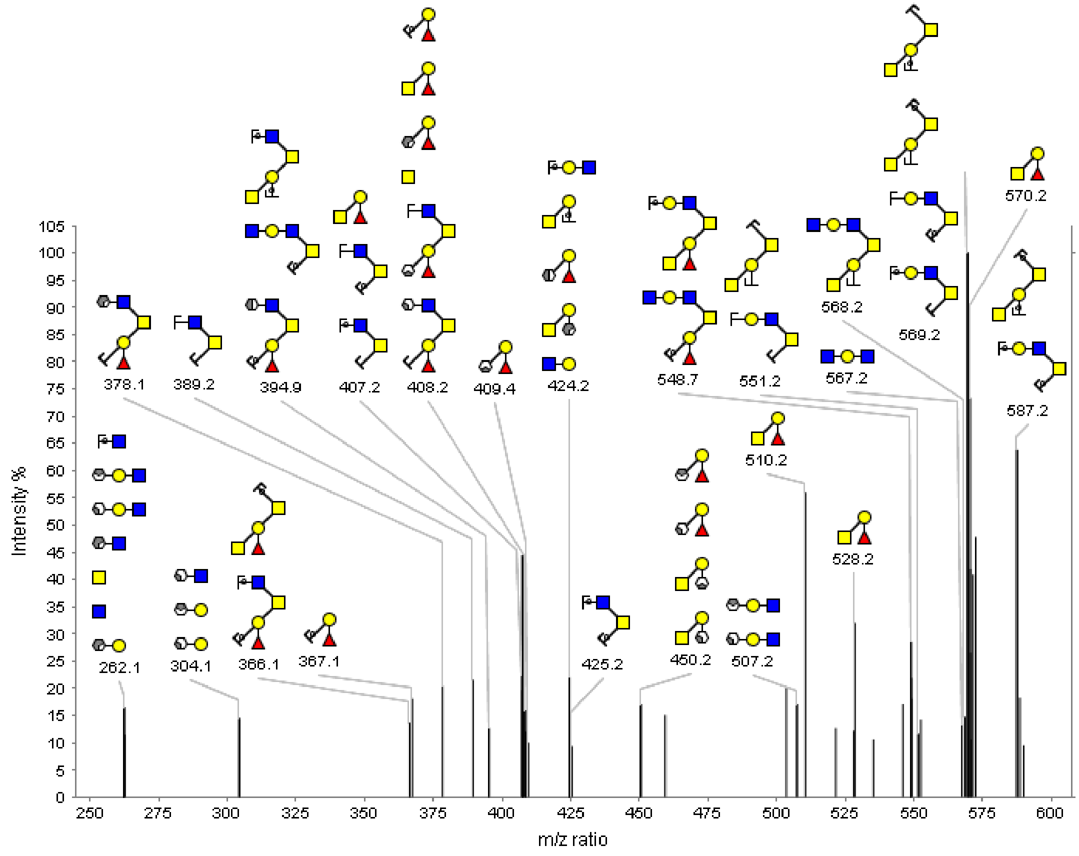

m/z range: 650-1150

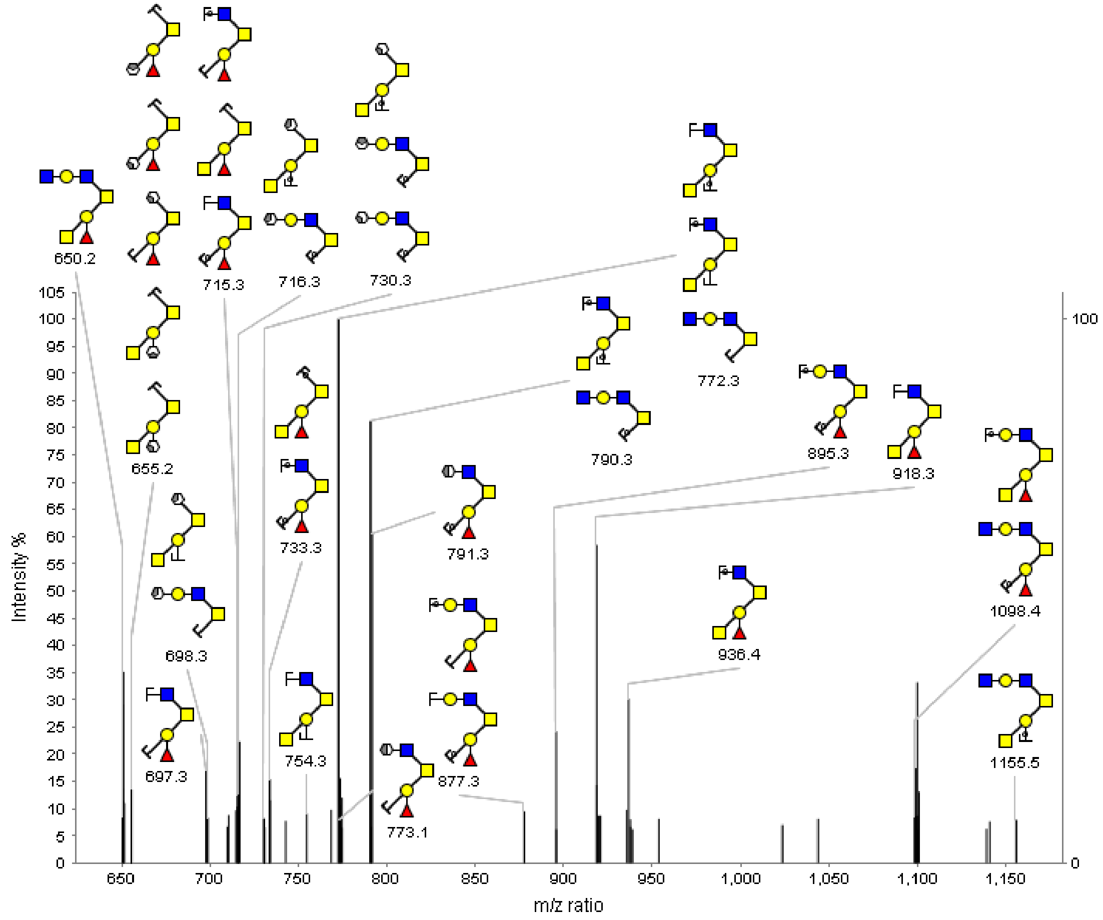

Glycan code: 650-1

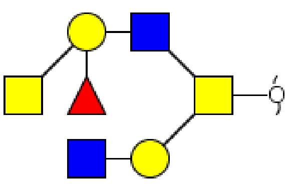

m/z: 650.2
RT: 20.6 min
^{TWIMS}CCS: 387 Å
^{TIMS}CCS: 381 Å

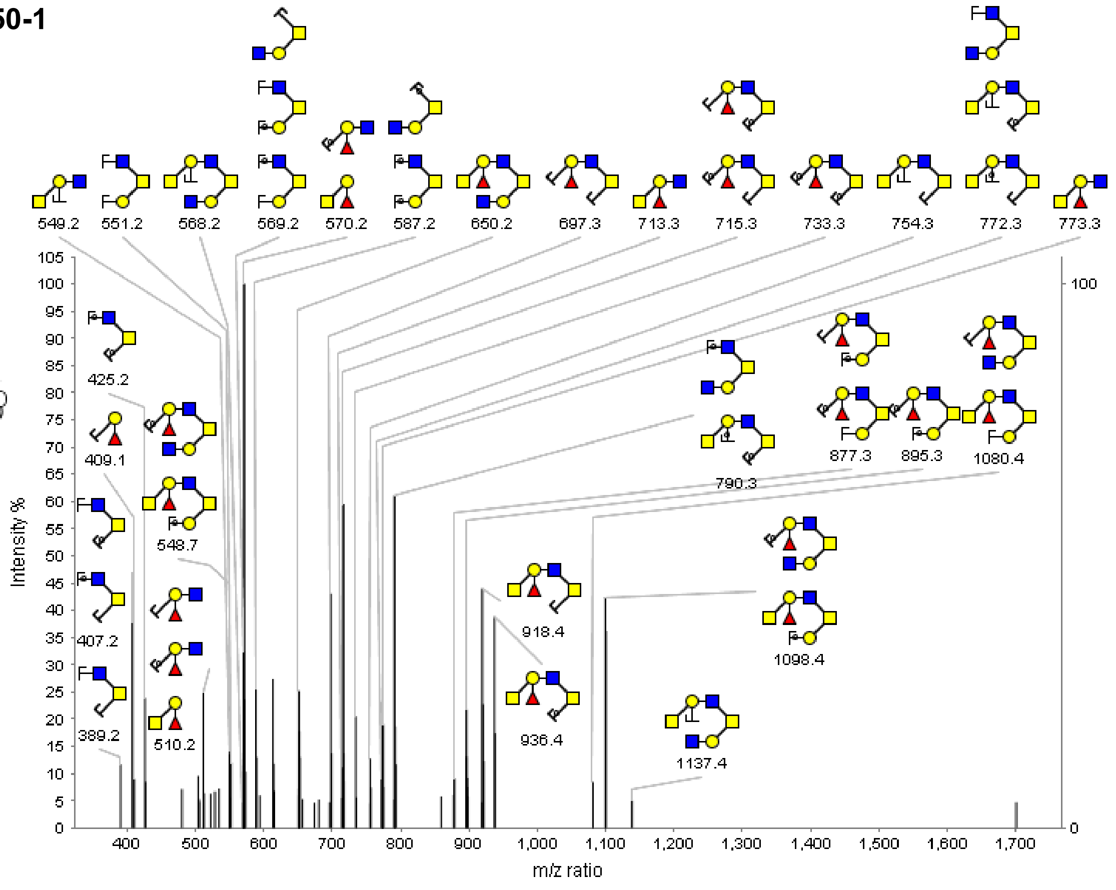

Glycan code: 702-1

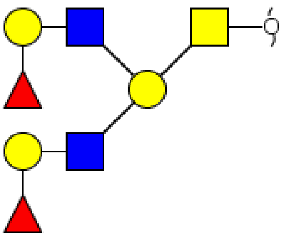

m/z: 702.8
RT: 23.4 min
^{TWIMS}CCS: 374 Å
^{TIMS}CCS: 361 Å

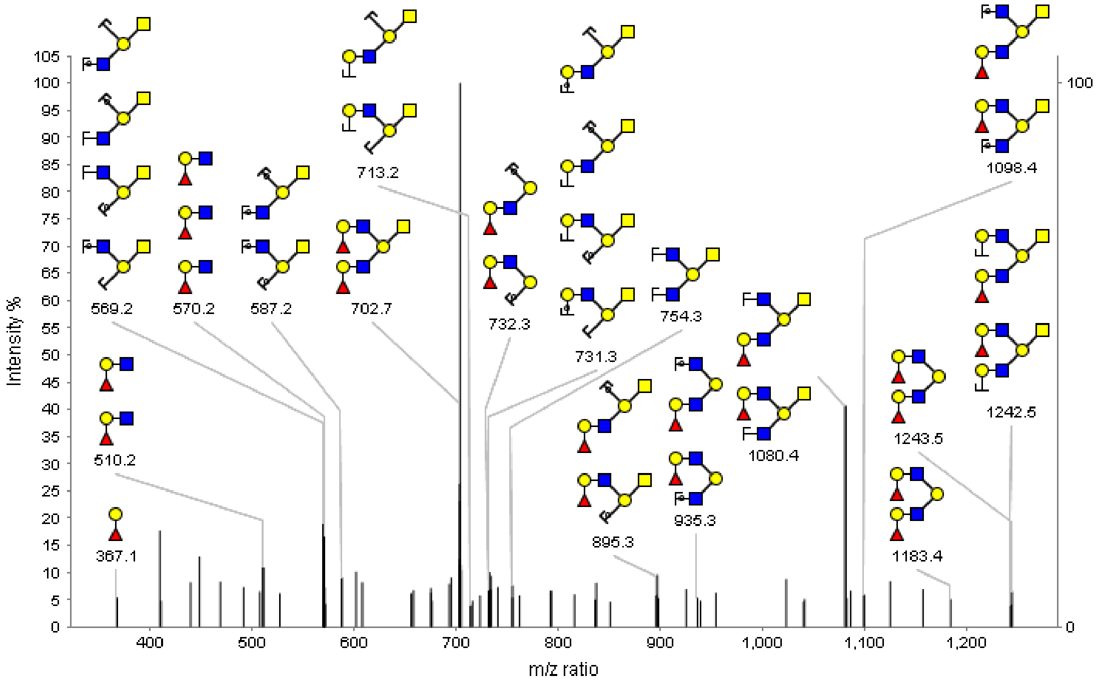

Glycan code: 702-2

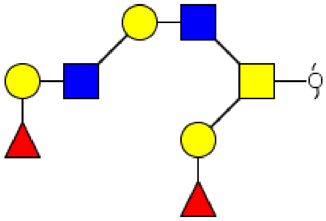

m/z: 702.8
RT: 25.8 min
^{TWIMS}CCS: 376 Å
^{TIMS}CCS: 369 Å

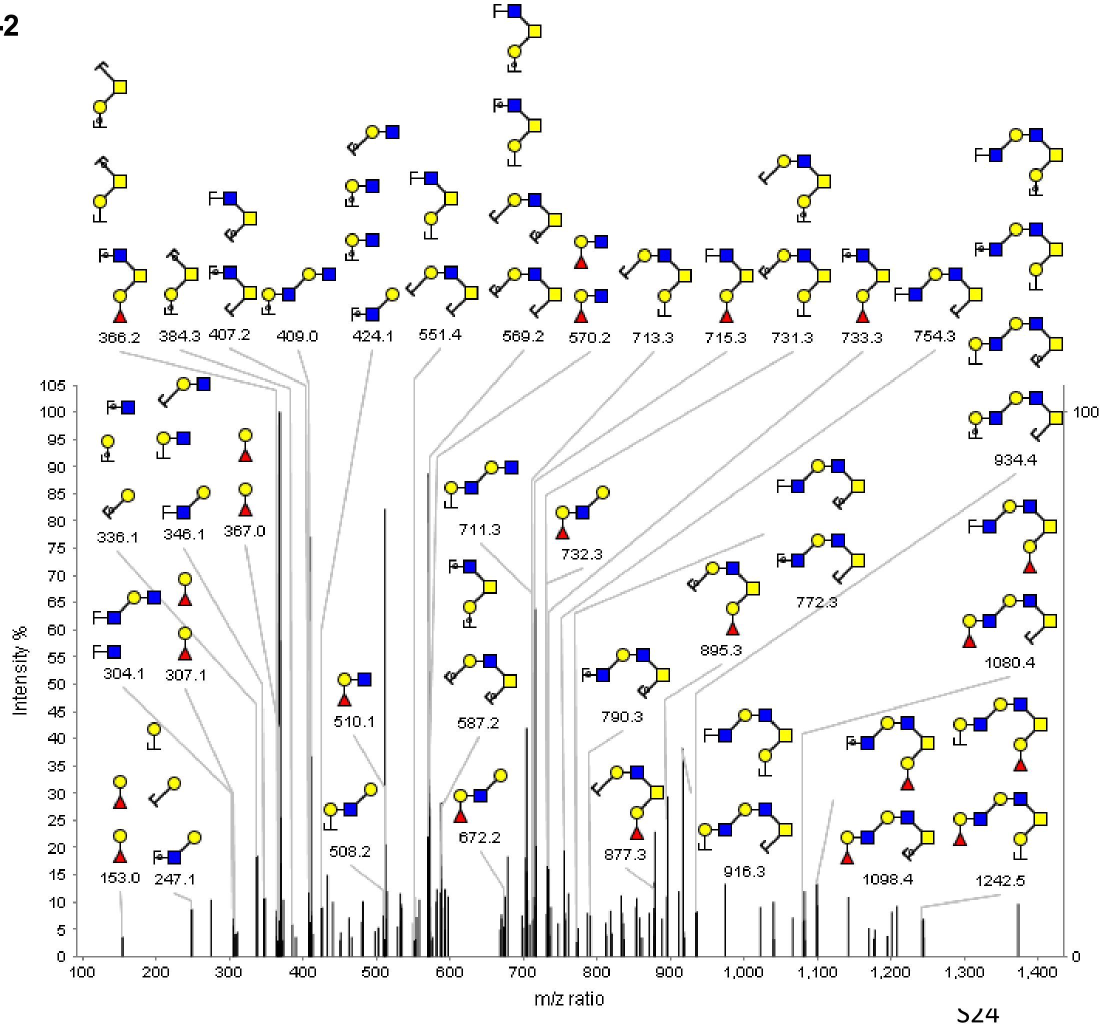

Glycan code: 702-3

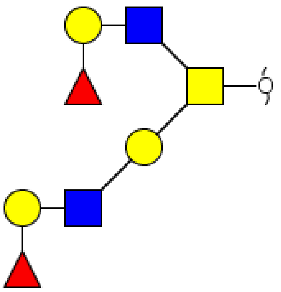

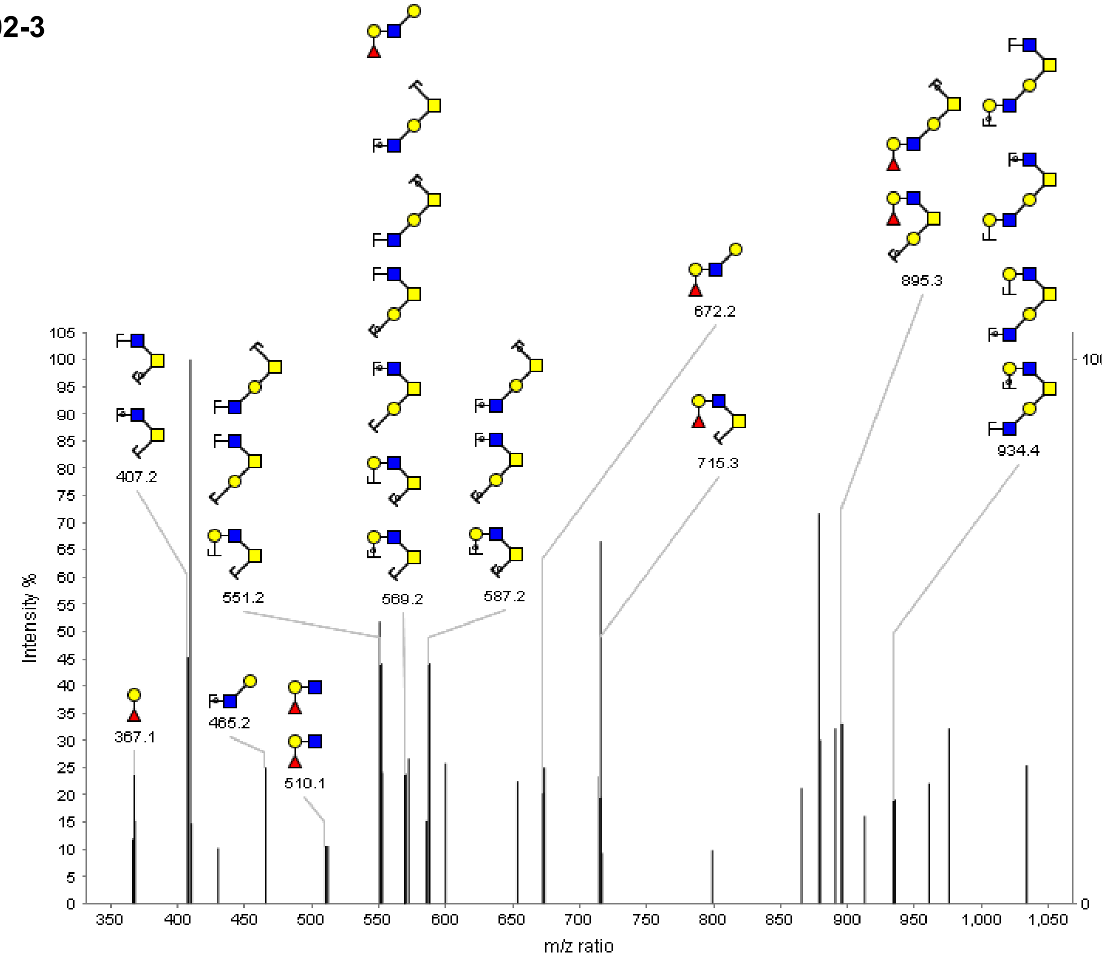

m/z: 702.8
TWIMSCCS: n.d.
TIMSCCS: 379 Å

Glycan code: 723-1

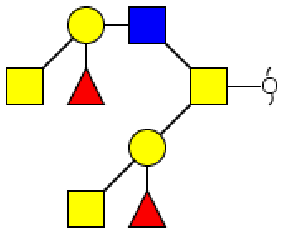

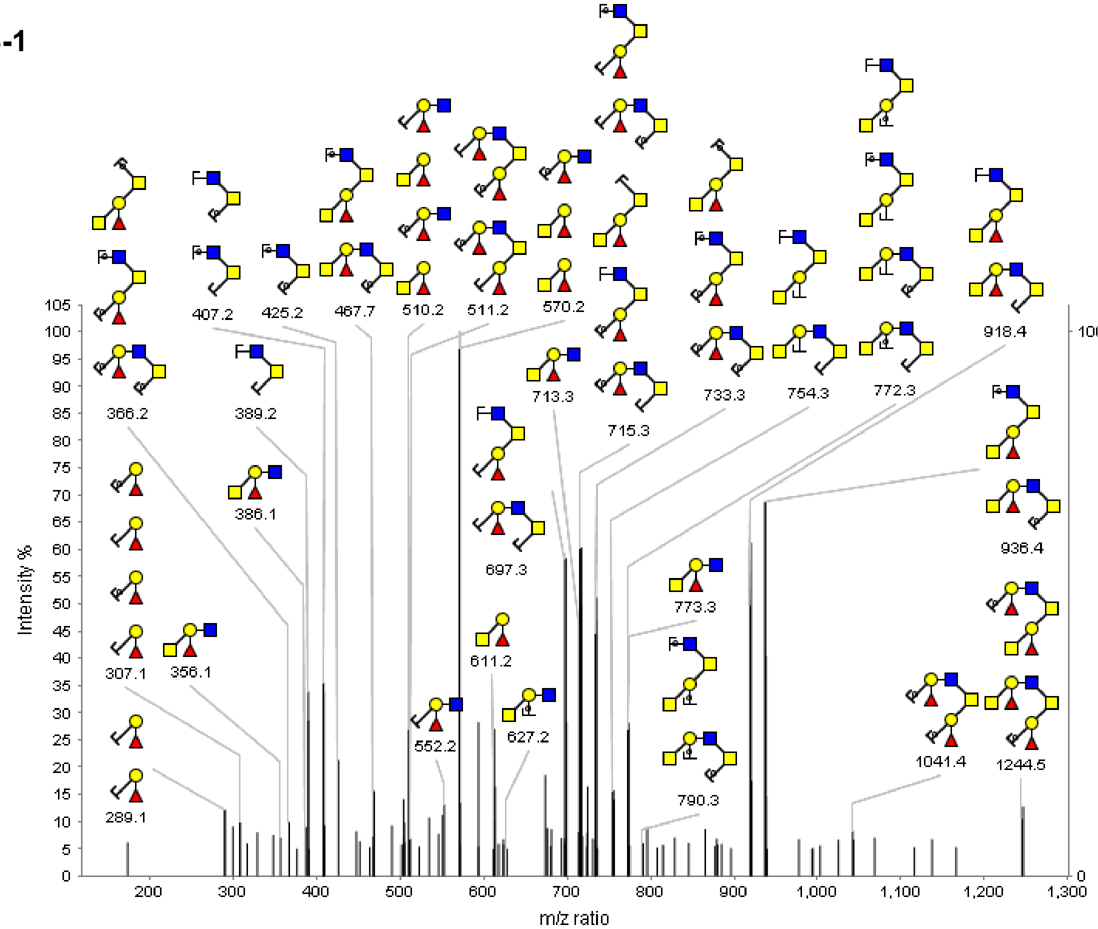

m/z: 723.3
RT: 19.6 min
TWIMSCCS: 372 Å
TIMSCCS: 364 Å

Glycan code: 804-1

m/z range: 250-700

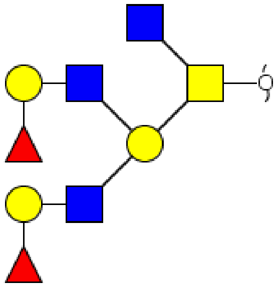

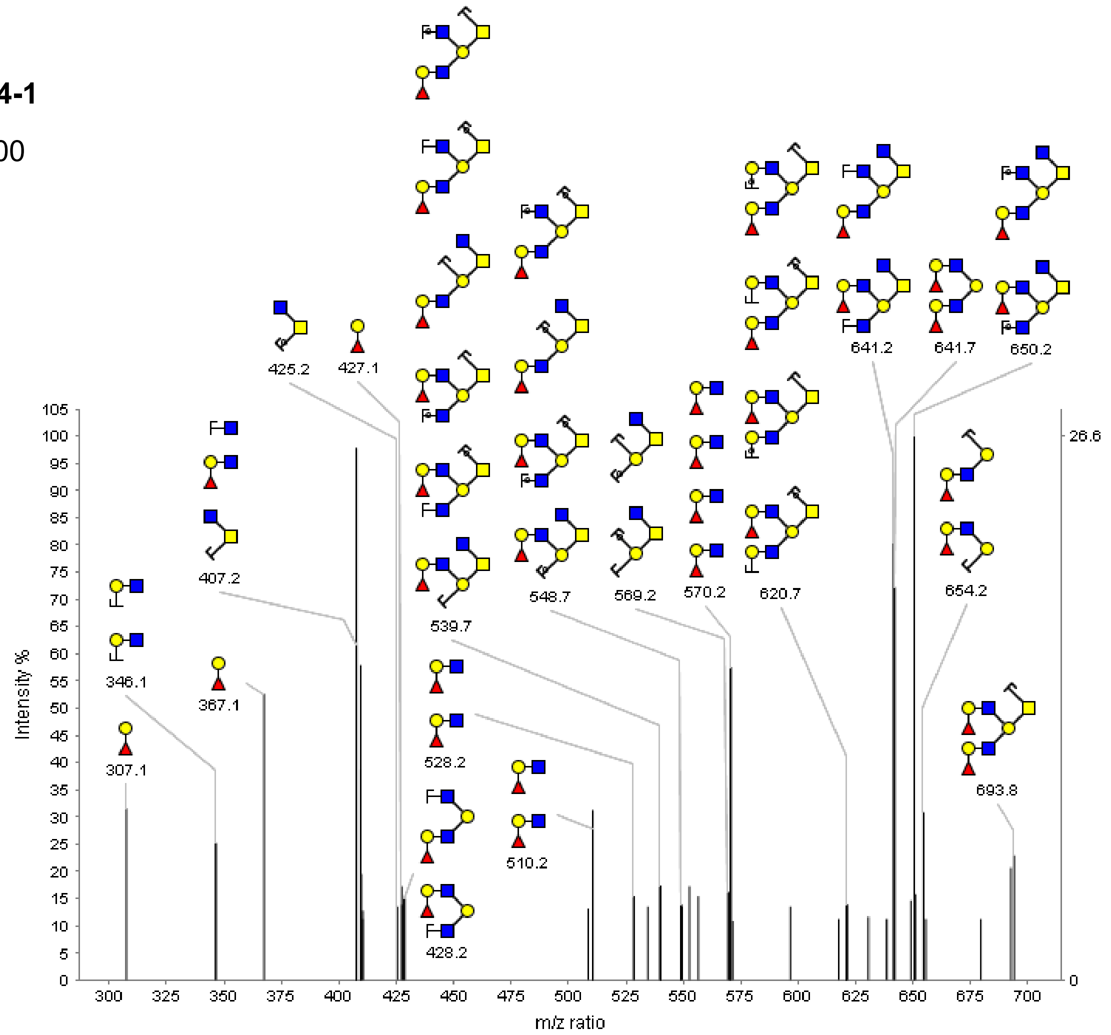

m/z: 804.3
RT: 25.7 min
TWIMSCCS: 406 Å
TIMSCCS: 399 Å

m/z range: 700-1400

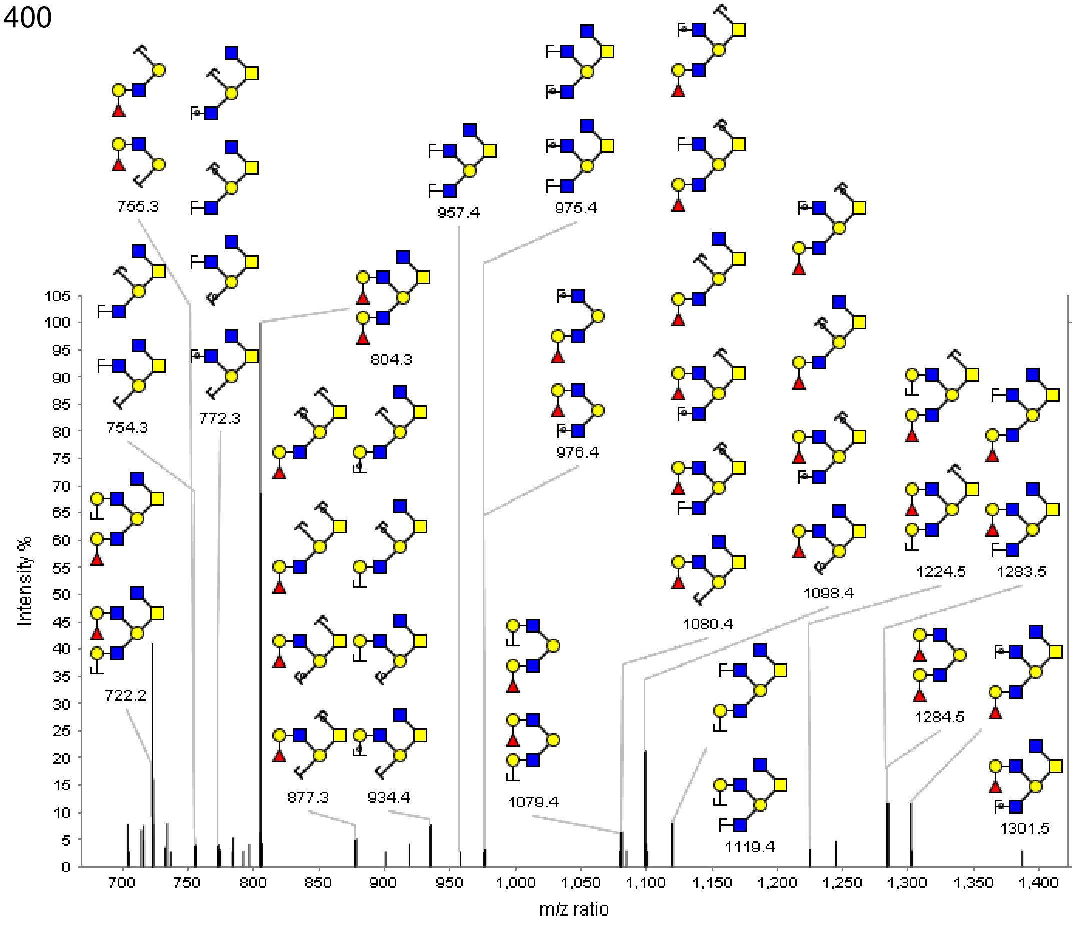

Glycan code: 861-1

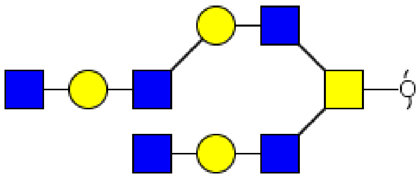

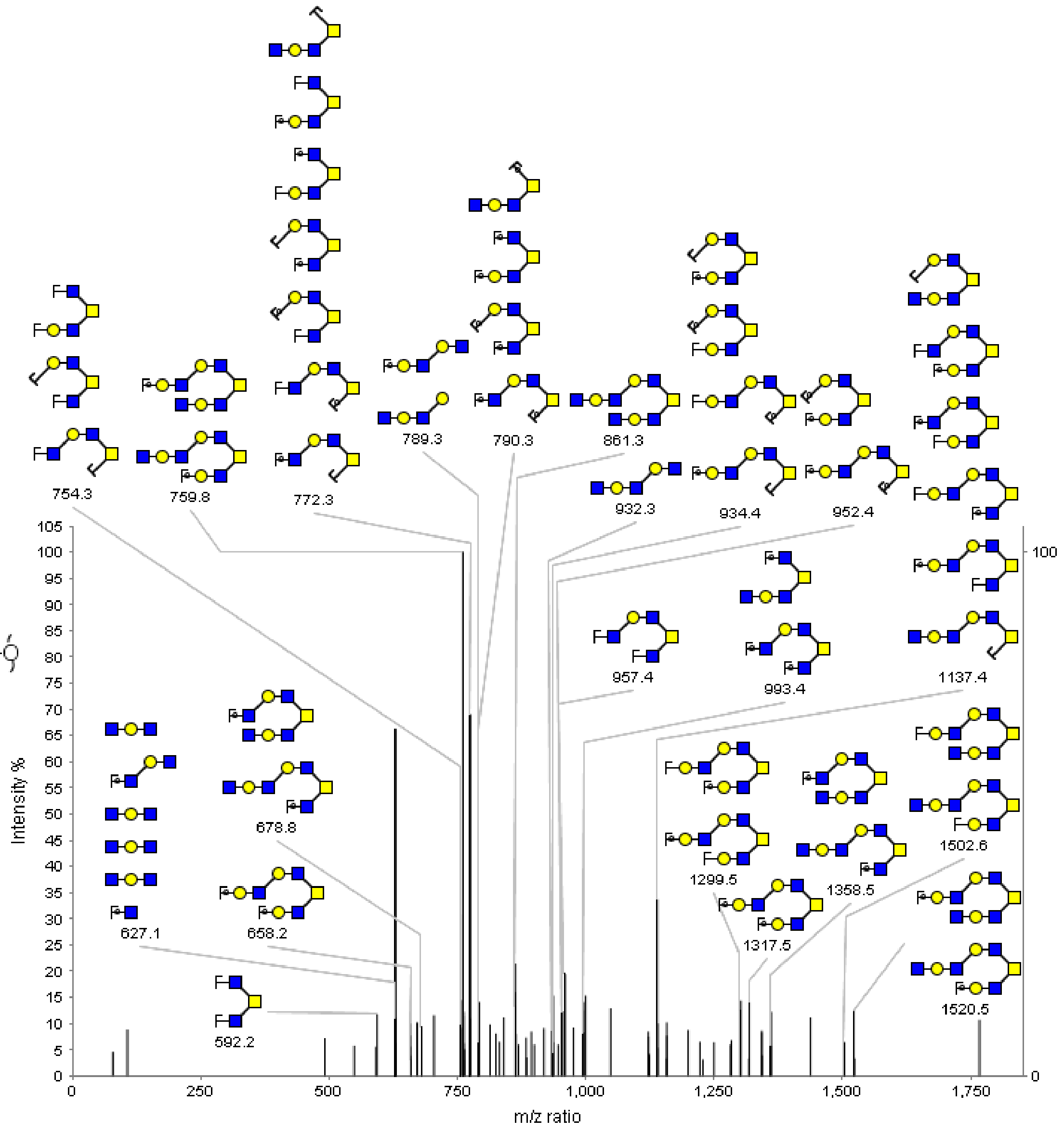

m/z: 861.3
RT: 20.5 min
TWIMSCCS: 411 Å
TIMSCCS: 401 Å

Glycan code: 861-2

m/z range: 200-700

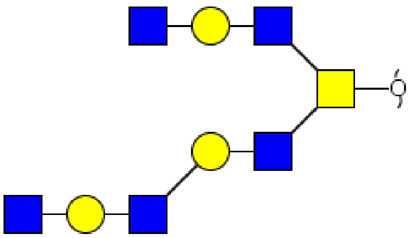

m/z: 861.3
RT: 20.0 min
^{TWIMS}CCS: 421 Å
^{TIMS}CCS: 411 Å

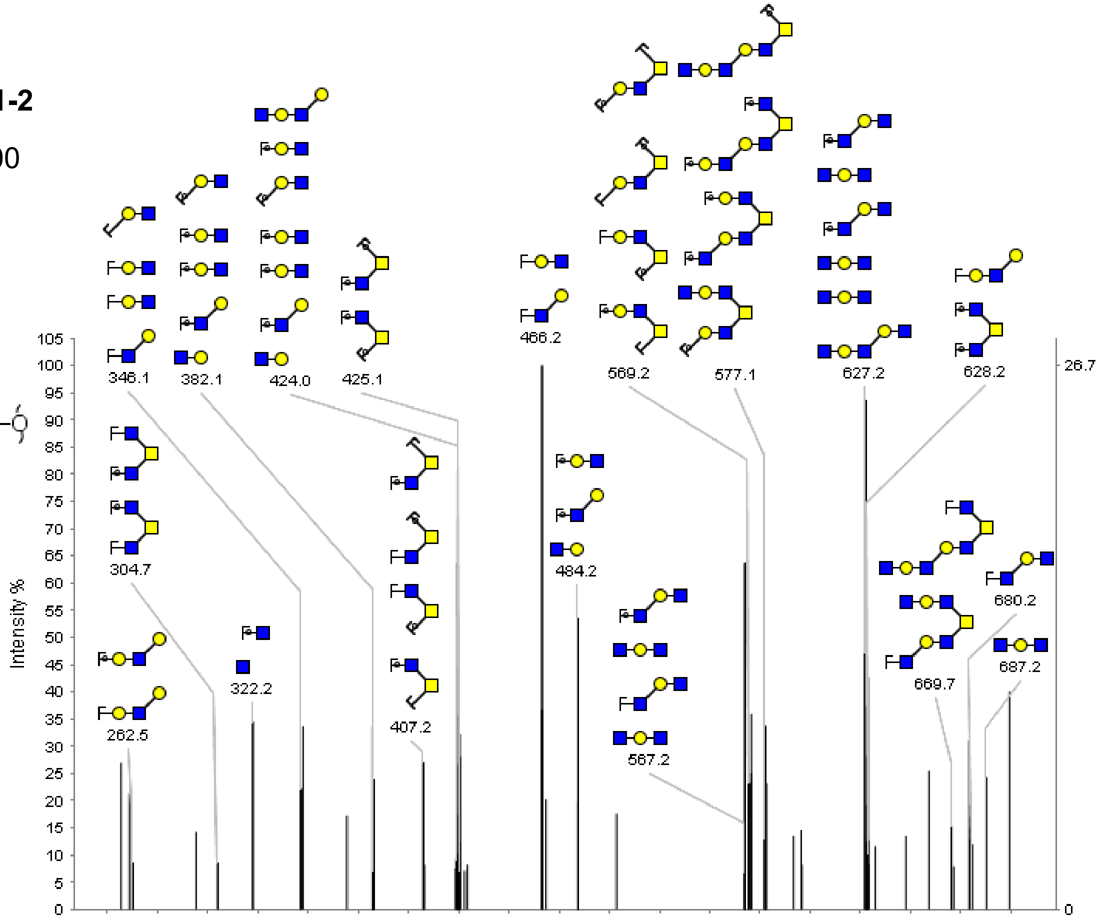

m/z range: 700-1550

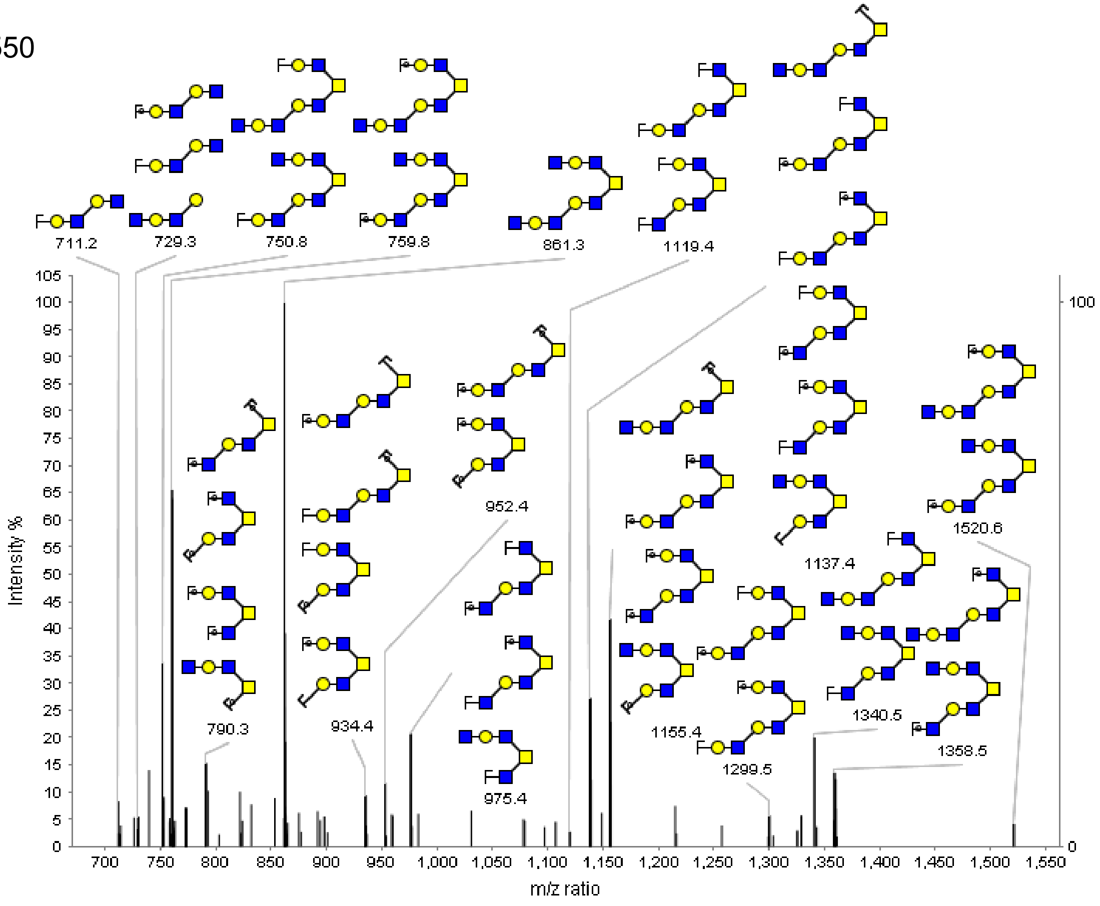

II. Annotated MS/MS spectra of *O*-glycans in healthy sputum

Glycan code: 530-1

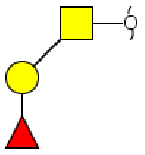

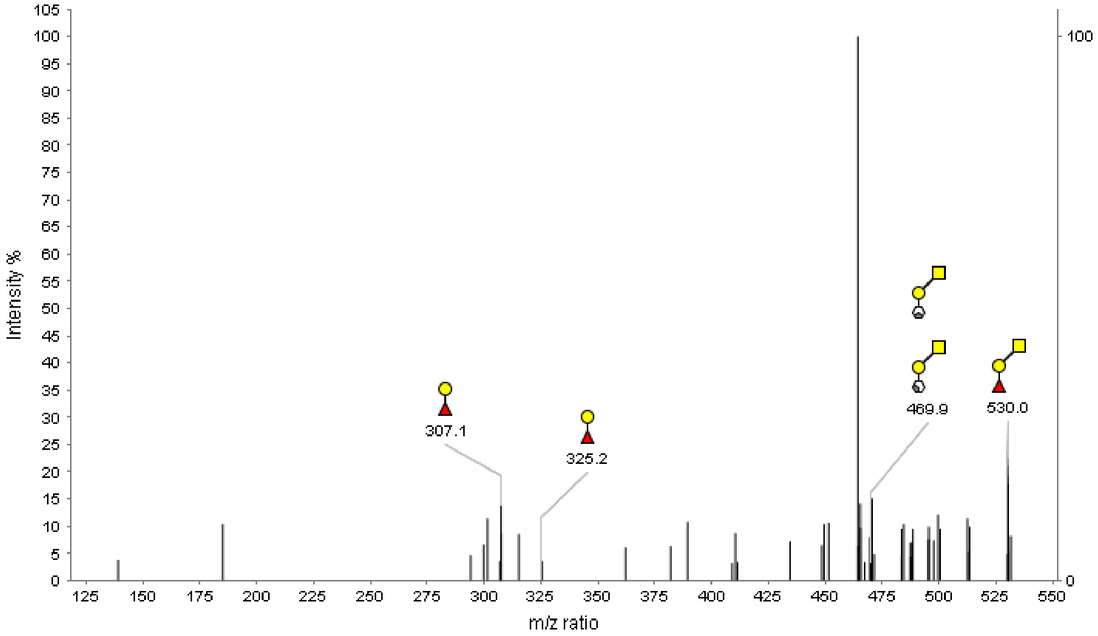

m/z: 530.21
^{TIMS}CCS: 211 Å

Glycan code: 587-1

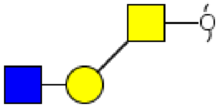

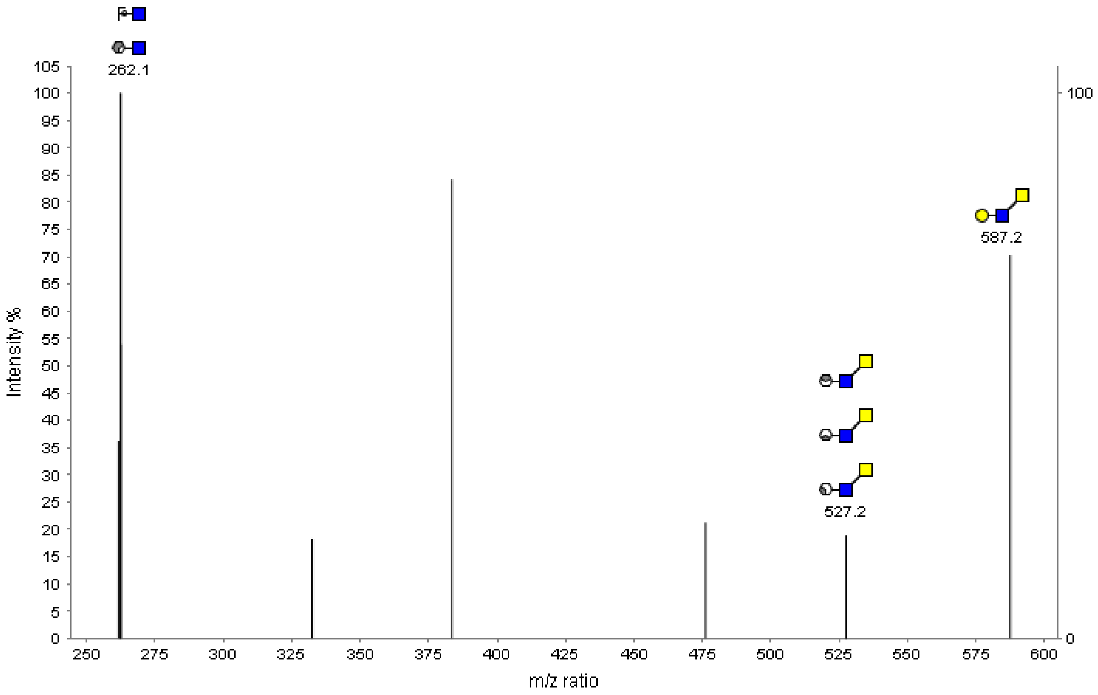

m/z: 587.23
^{TIMS}CCS: 217 Å

Glycan code: 587-2

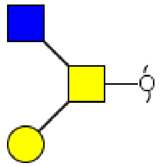

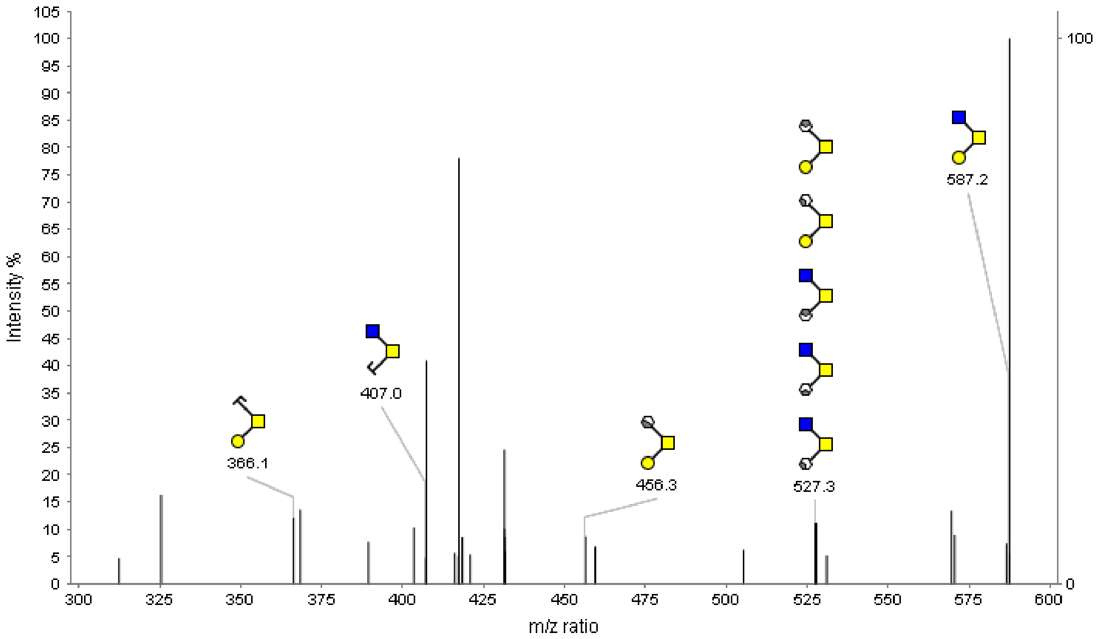

m/z: 587.23
^{TIMS}CCS: 234 Å

Glycan code: 675-1

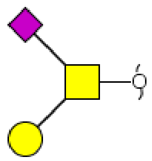

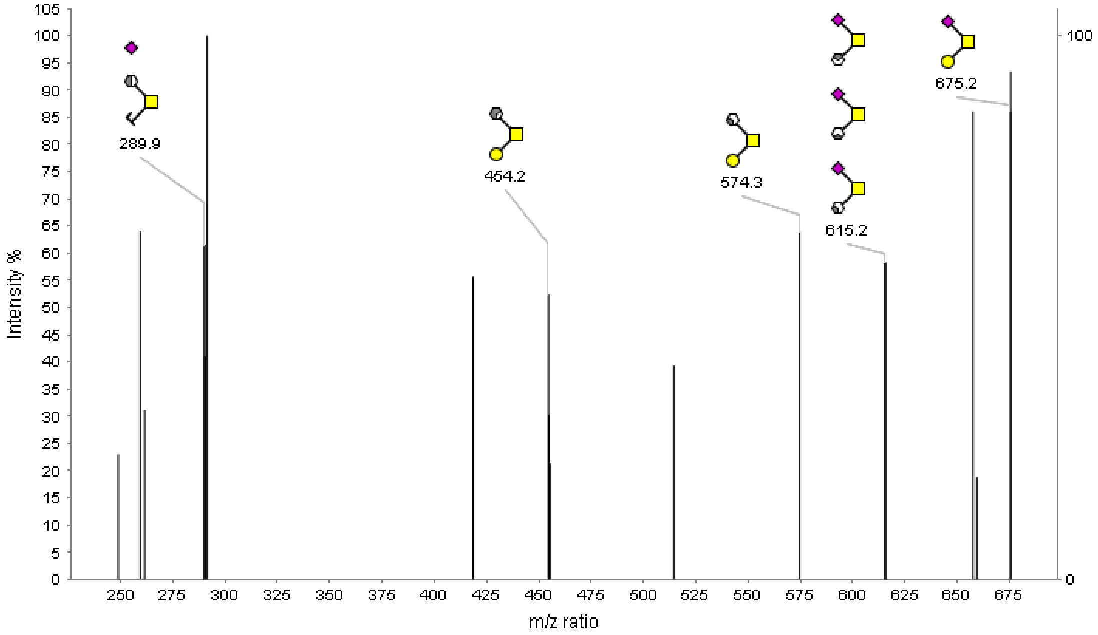

m/z: 675.24
^{TIMS}CCS: 246 Å

Glycan code: 675-2

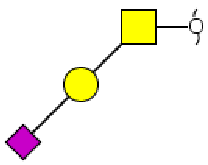

m/z: 675.24
^{TIMS}CCS: 251 Å

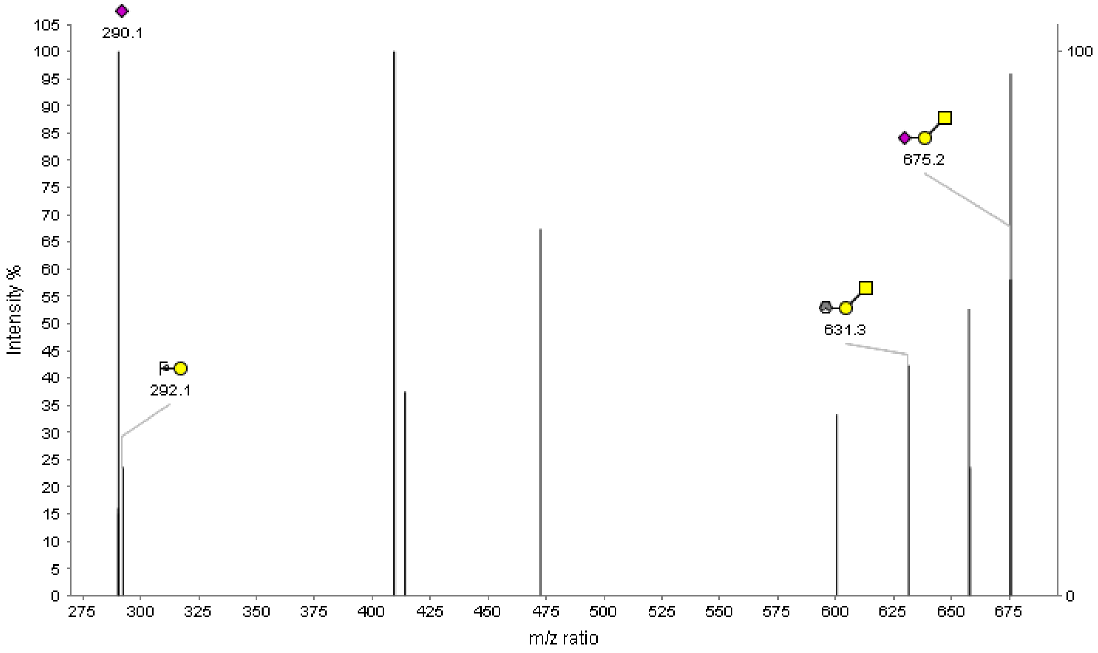

Glycan code: 716-1

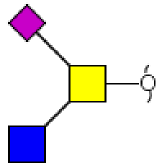

m/z: 716.27
^{TIMS}CCS: 262 Å

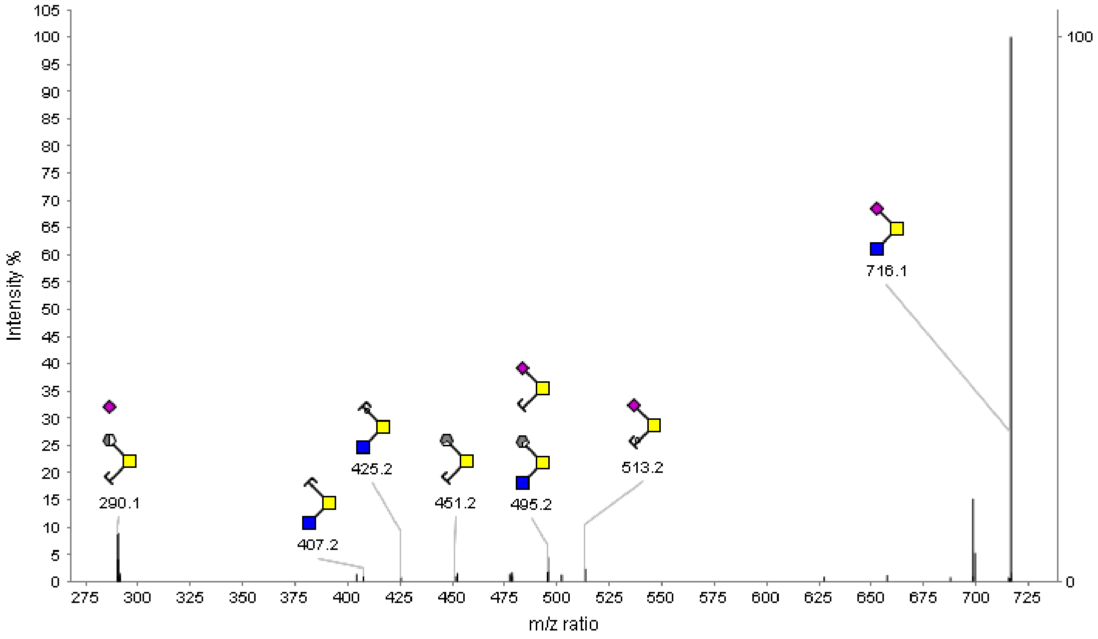

Glycan code: 733-1

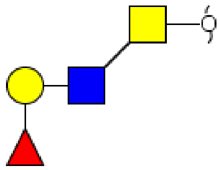

m/z: 733.29
^{TIMS}CCS: 253 Å

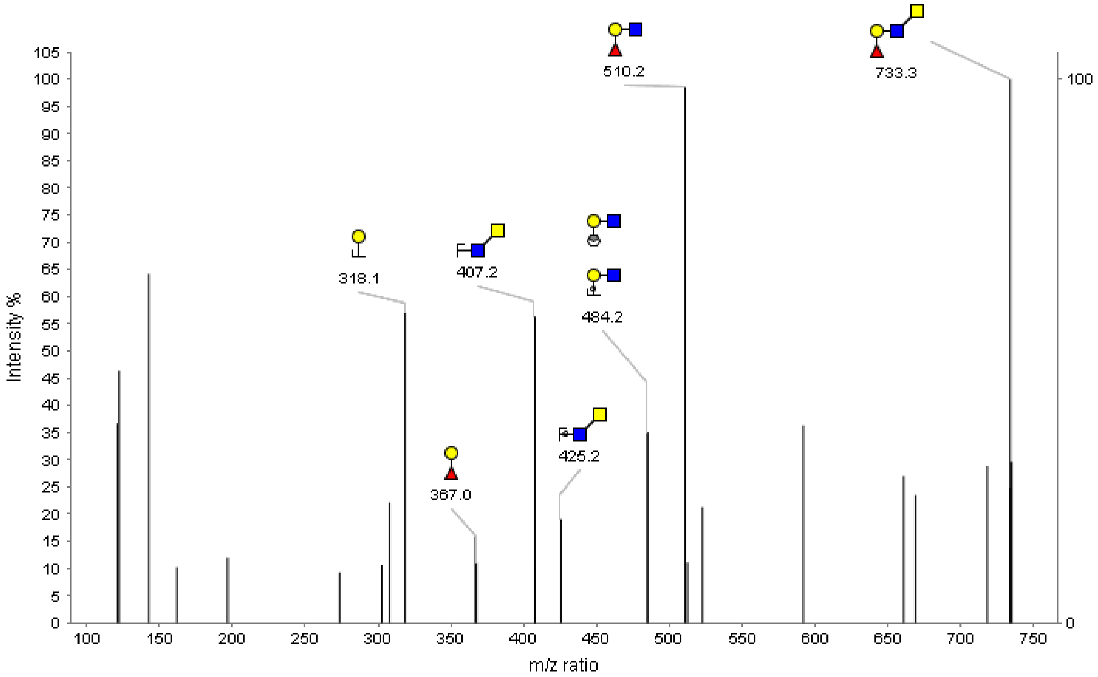

Glycan code: 733-2

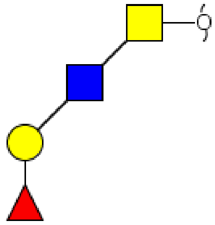

m/z: 733.29
^{TIMS}CCS: 257 Å

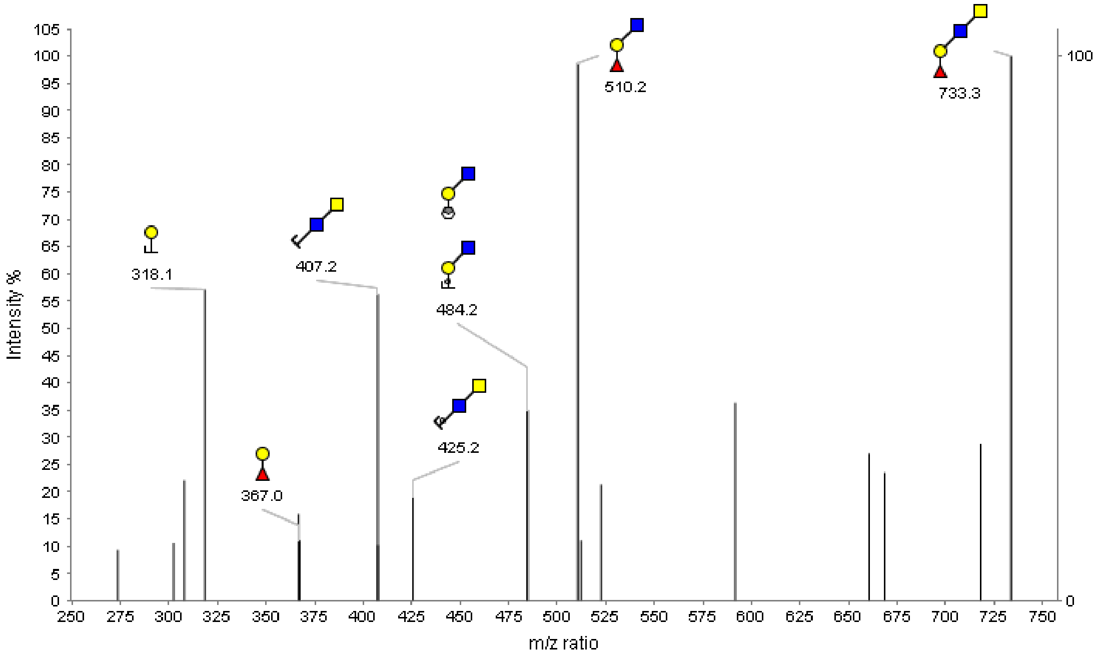

Glycan code: 749-1

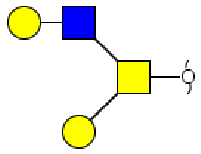

m/z: 749.28
TIMSCCS: 245 Å

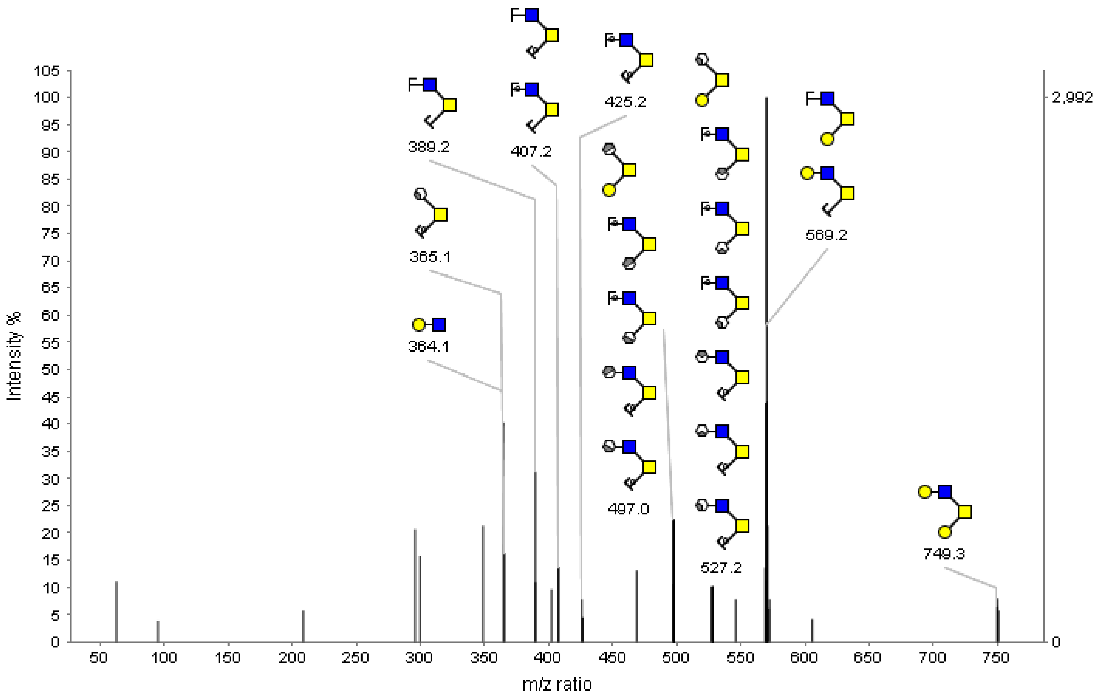

Glycan code: 749-2

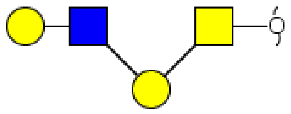

m/z: 749.28
TIMSCCS: 281 Å

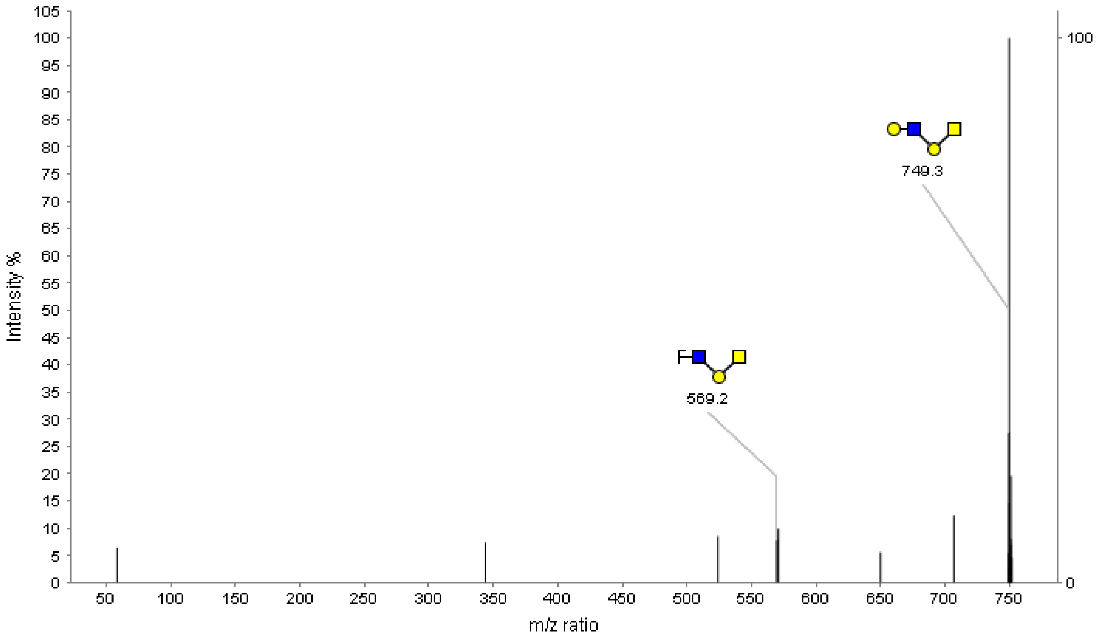

Glycan code: 790-1

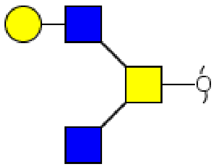

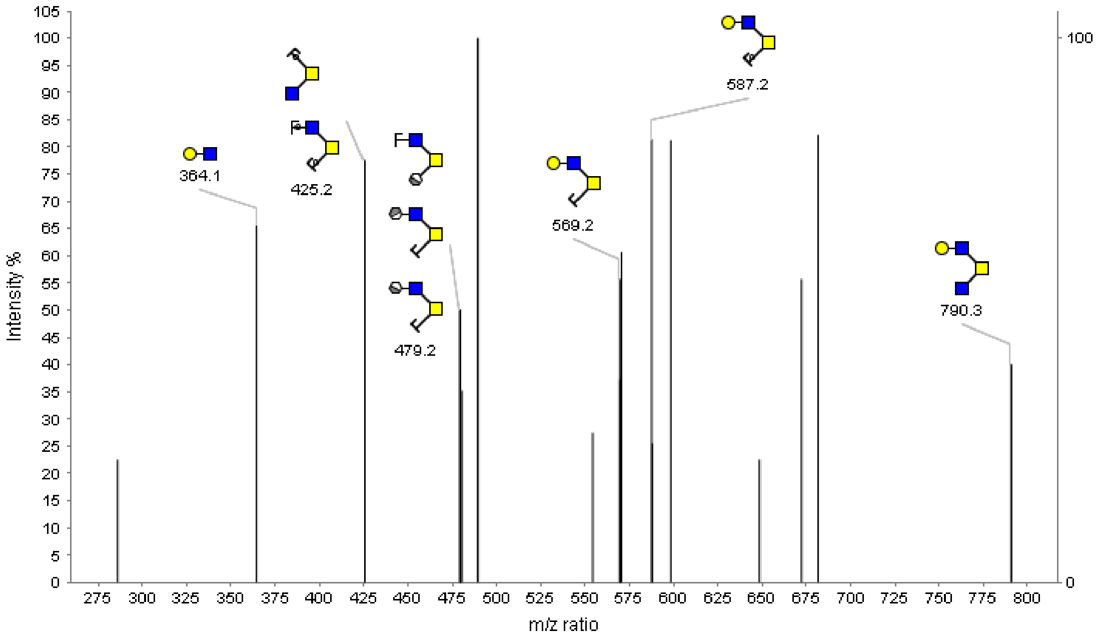

m/z: 790.31
^{TIMS}CCS: 254 Å

Glycan code: 790-2

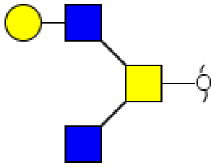

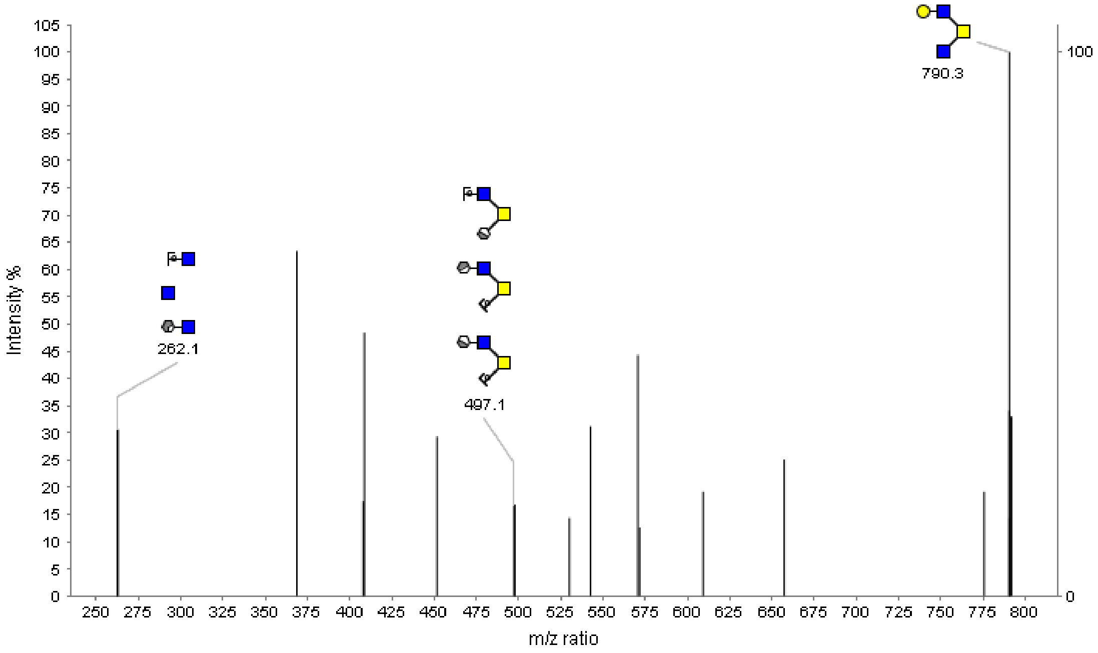

m/z: 790.31
^{TIMS}CCS: 266 Å

Glycan code: 813-1

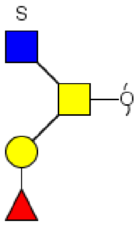

m/z: 813.24
TIMSCCS: 256 Å

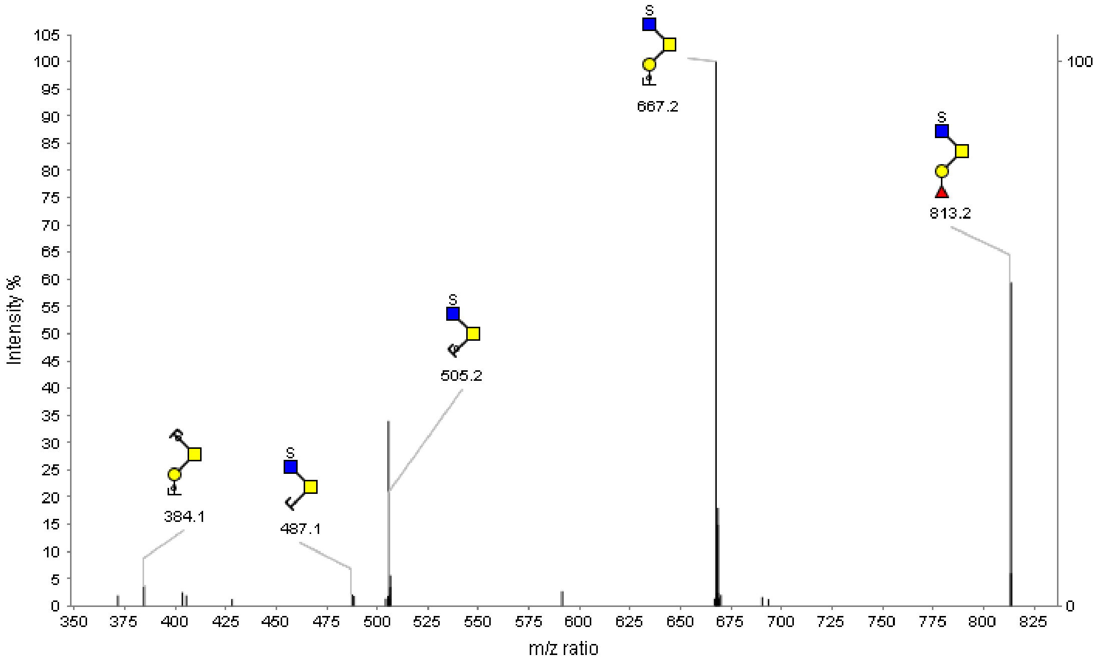

Glycan code: 878-1

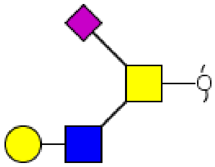

m/z: 878.32
TIMSCCS: 275 Å

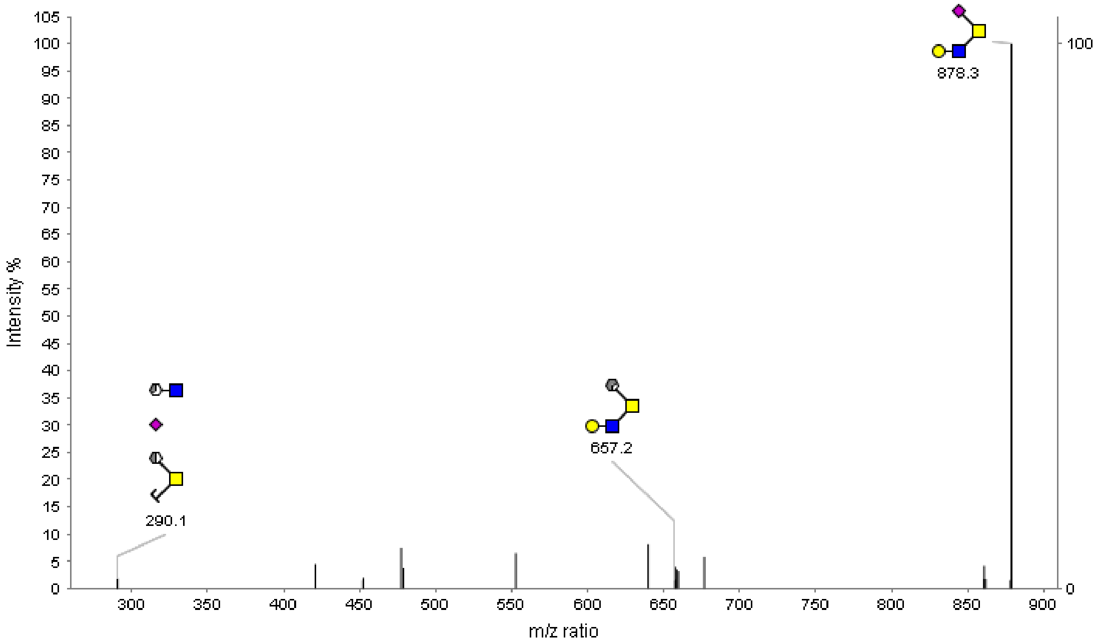

Glycan code: 895-1

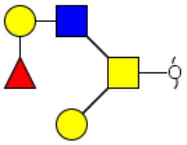

m/z: 895.34
^{TIMS}CCS: 274 Å

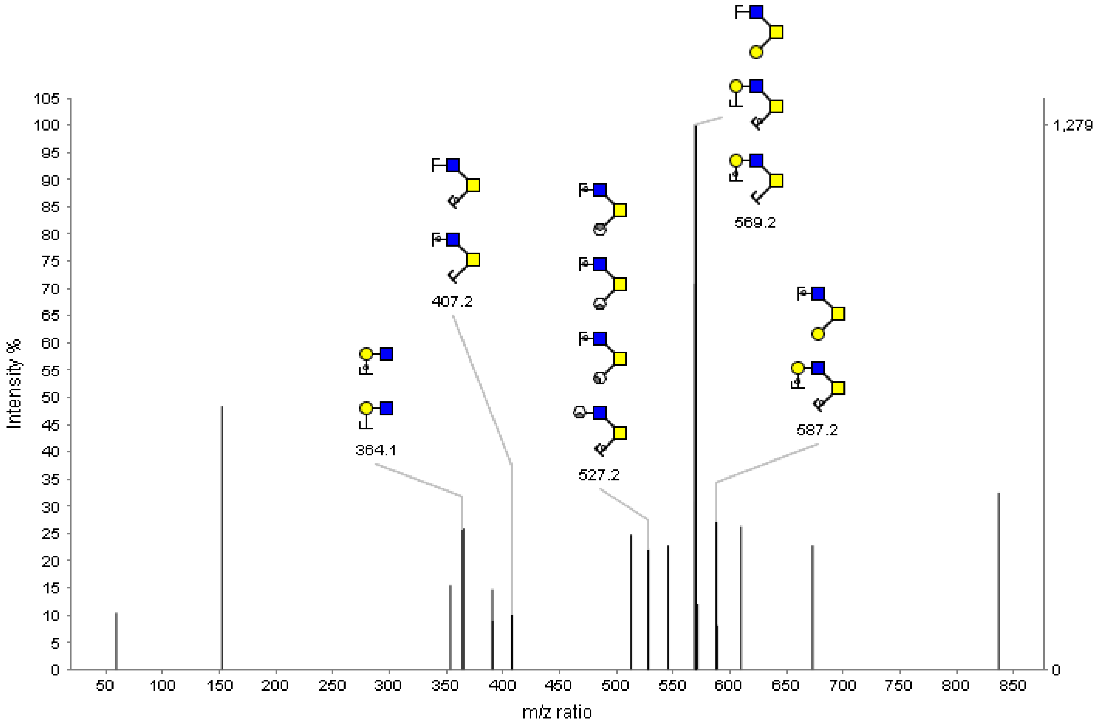

Glycan code: 895-2

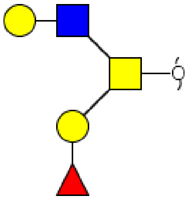

m/z: 895.34
^{TIMS}CCS: 275 Å

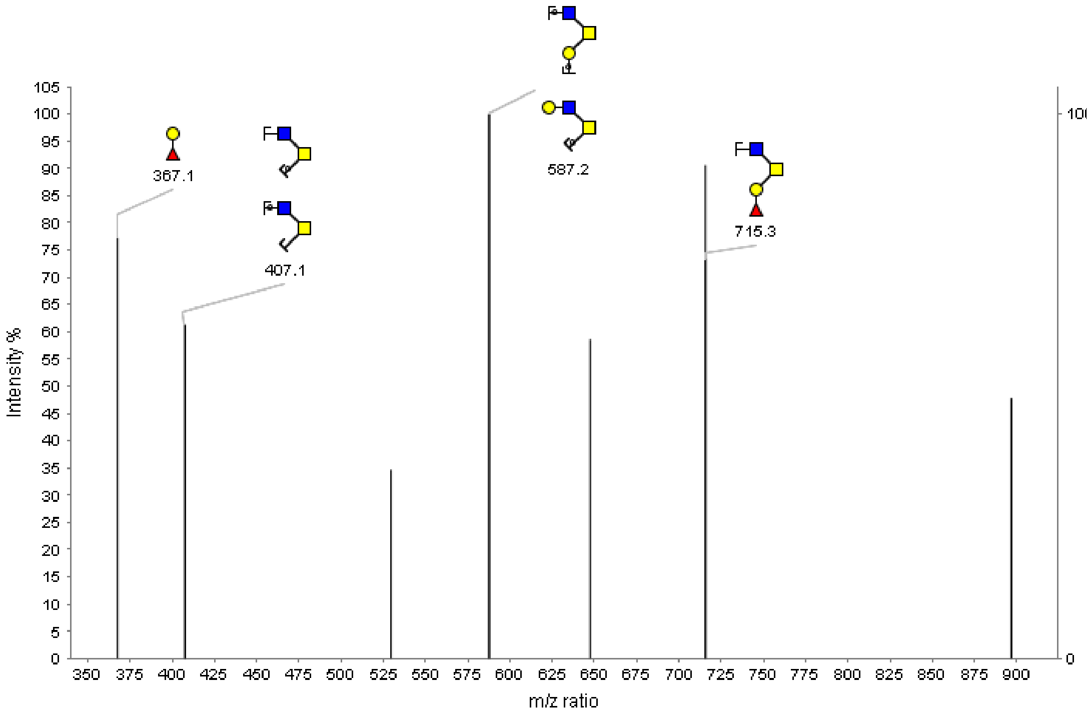

Glycan code: 895-3

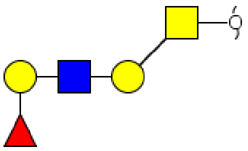

m/z: 895.34
TIMSCCS: 291 Å

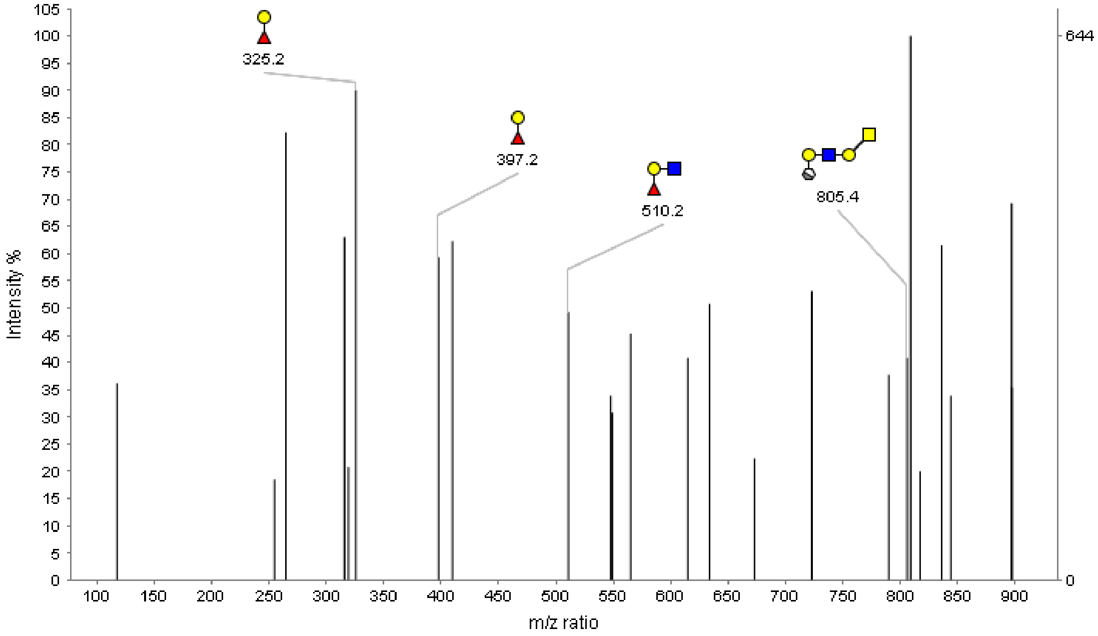

Glycan code: 895-4

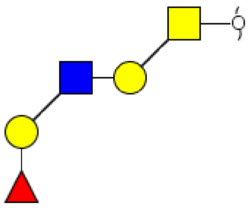

m/z: 895.3
RT: 18.0 min
TWIMSCCS: 298 Å
TIMSCCS: 297 Å

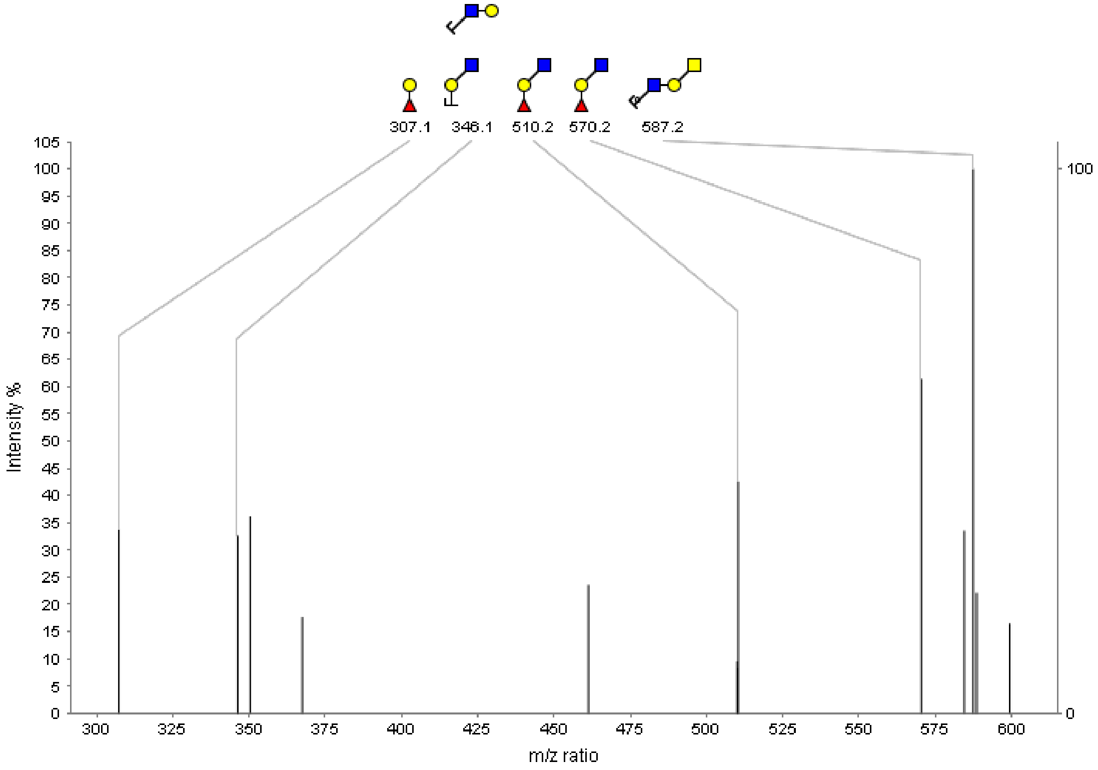

Glycan code: 975-1

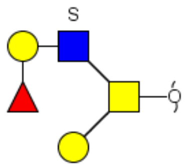

m/z: 975.30
TIMSCCS: 278 Å

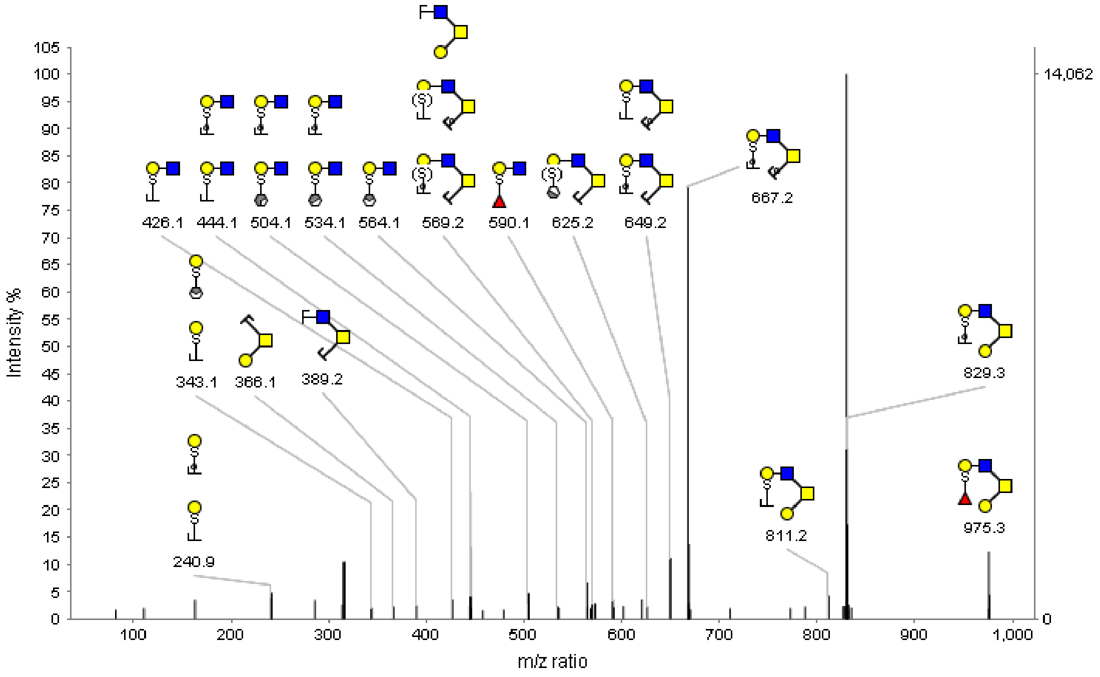

Glycan code: 551-1

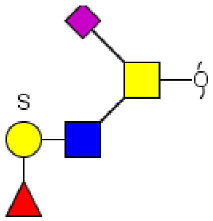

m/z: 551.57
TIMSCCS: 312 Å

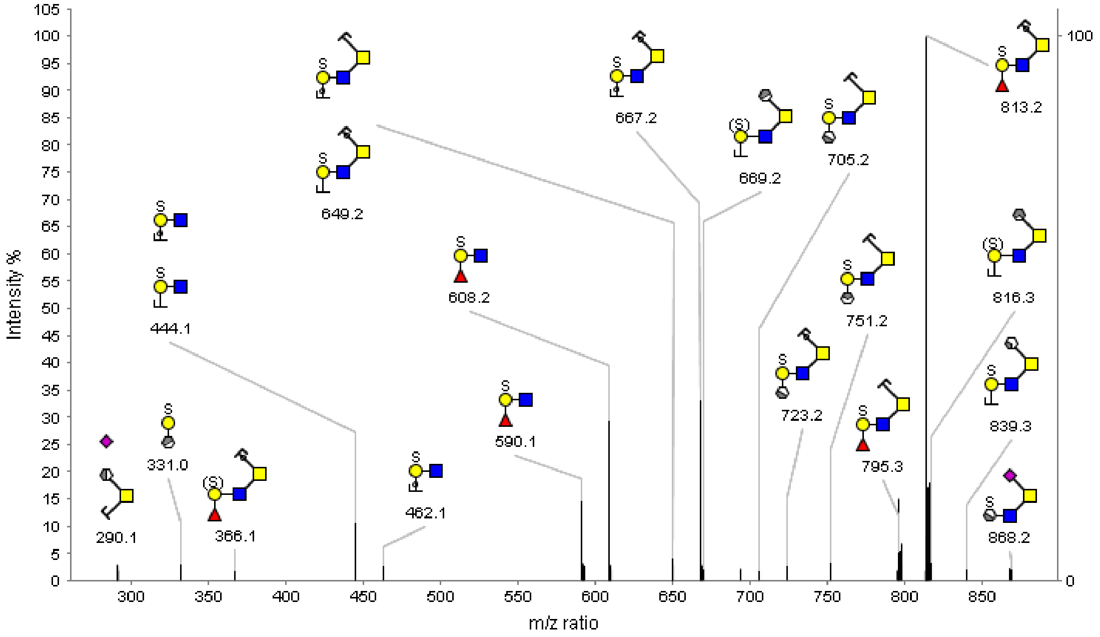

Glycan code: 560-1

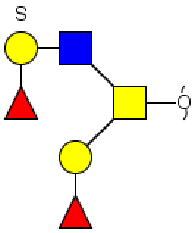

m/z: 560.17

TIMSCCS: 334 Å

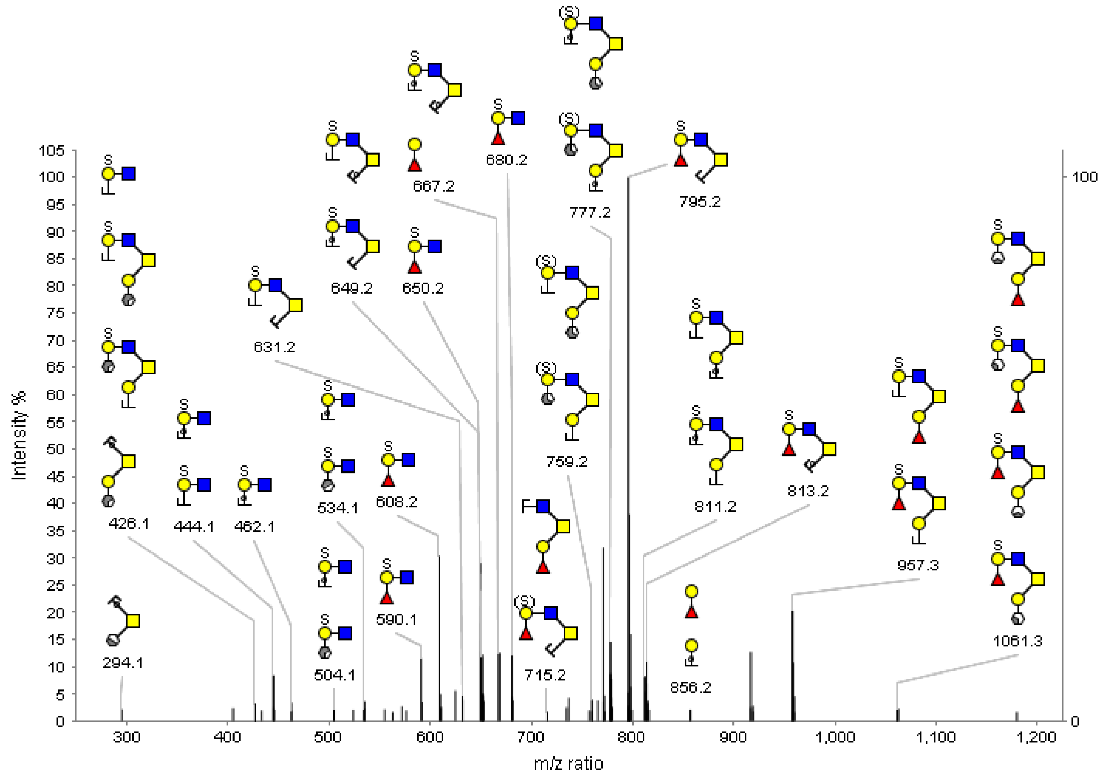

Glycan code: 588-1

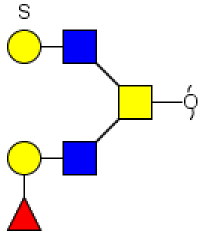

m/z: 588.68

TIMSCCS: 321 Å

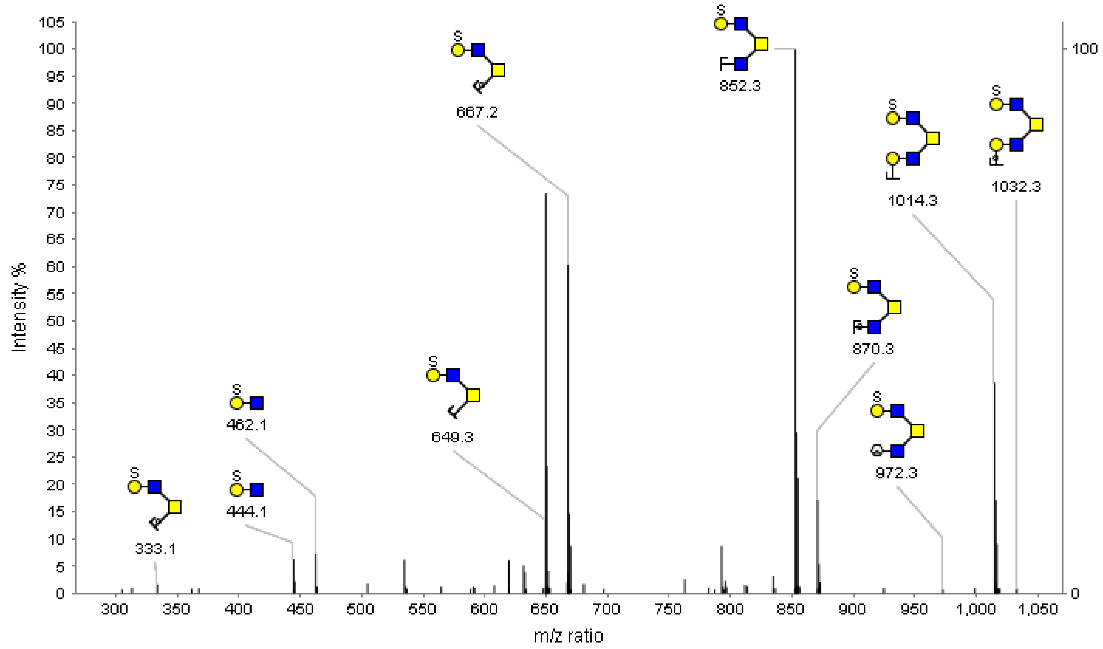

Glycan code: 588-2

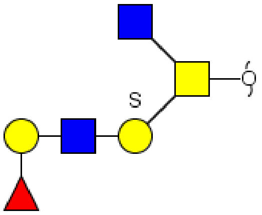

m/z: 588.68
^{TIMS}CCS: 326 Å

m/z range: 375-700

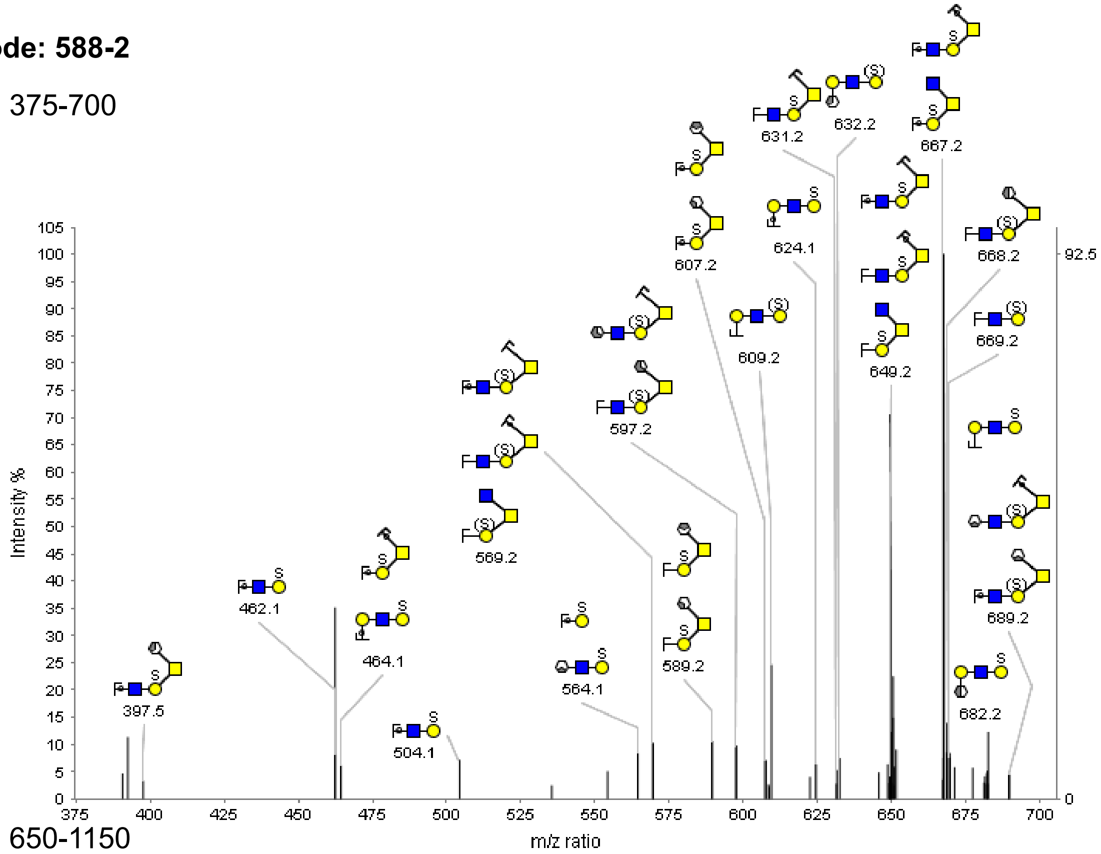

m/z range: 650-1150

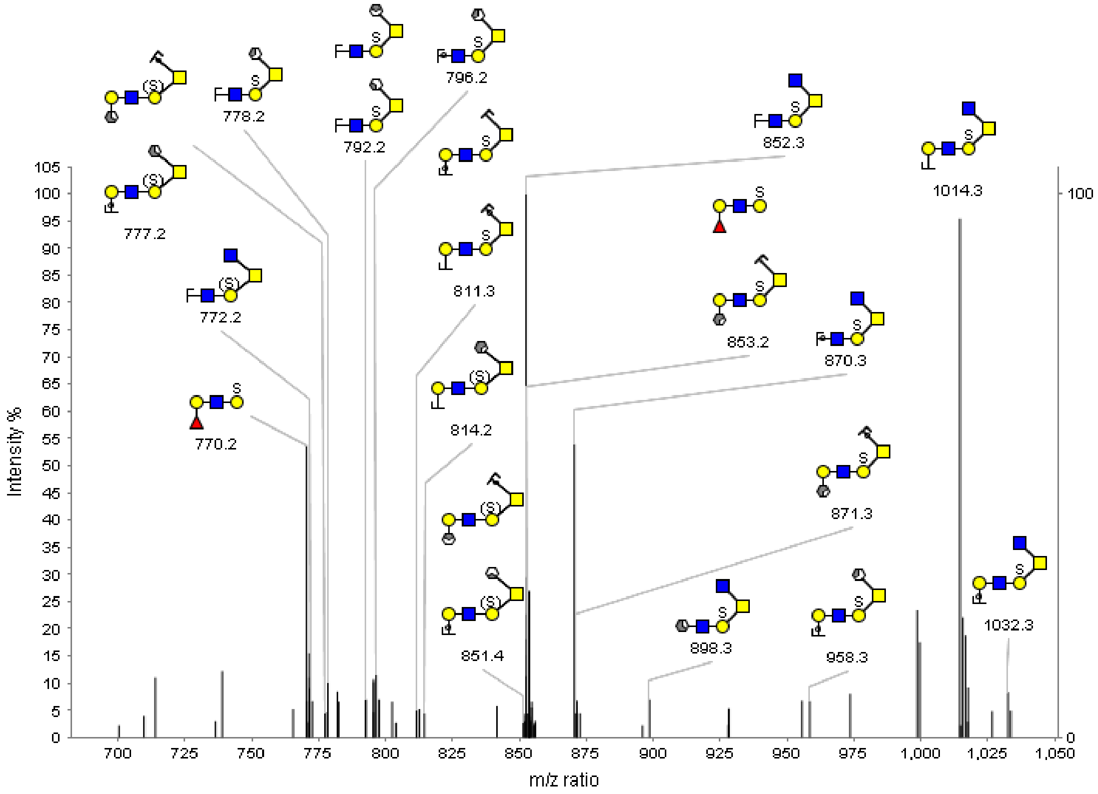

Glycan code: 632-1

m/z range: 250-700

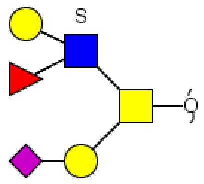

m/z: 632.69
^{TIMS}CCS: 338 Å

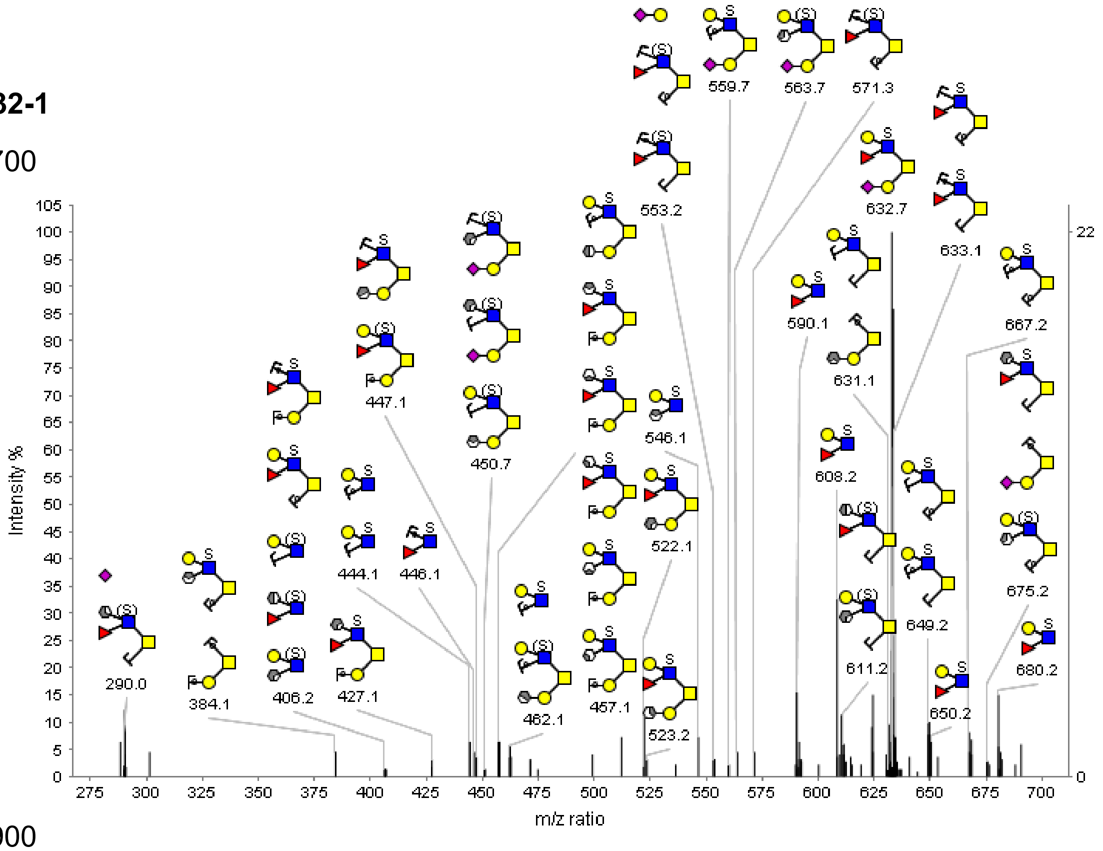

m/z range: 700-900

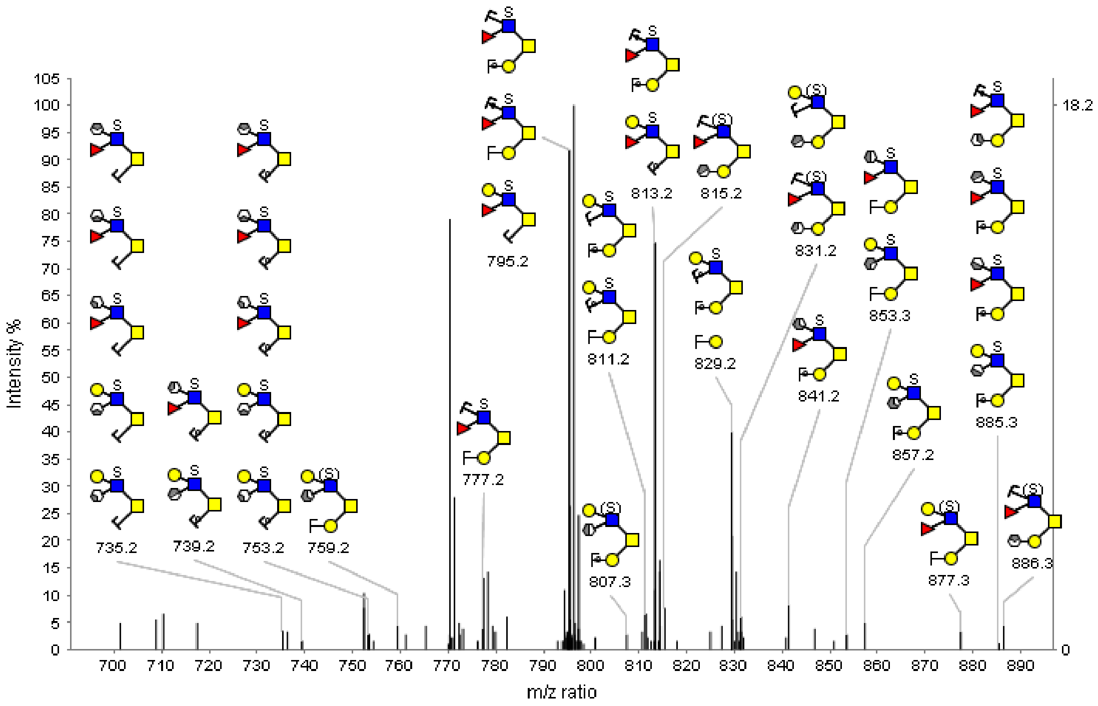

m/z range: 900-1175

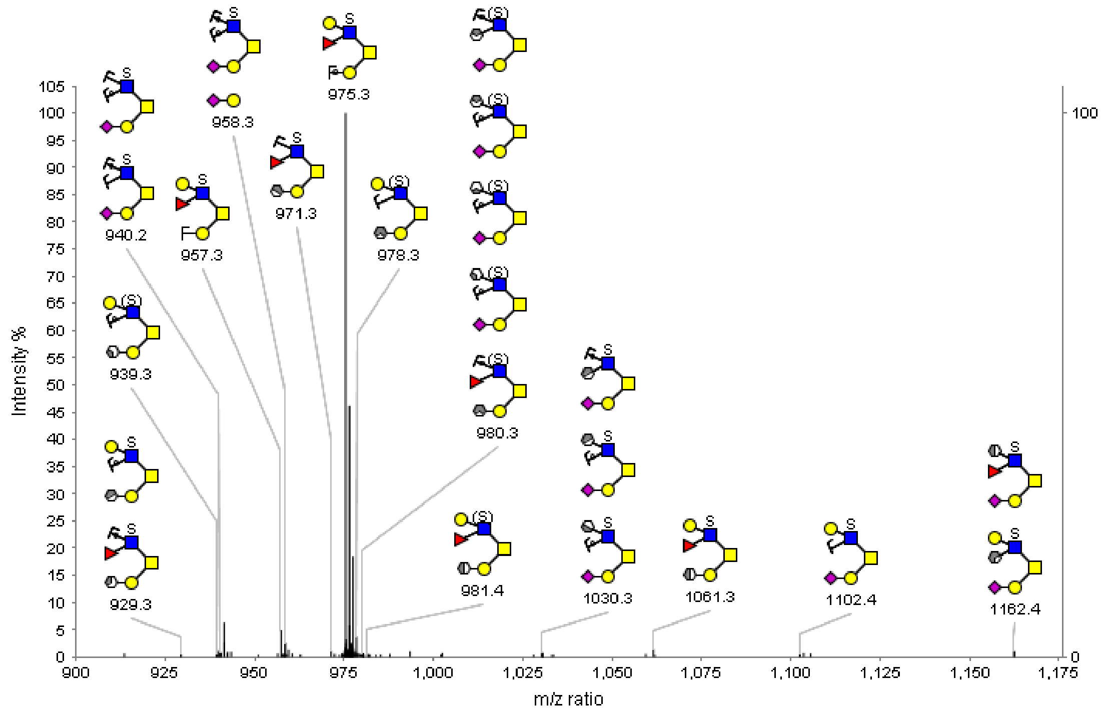

Glycan code: 738-1

m/z range: 100-750

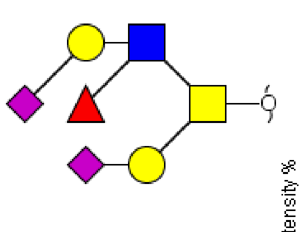

m/z: 738.26

^{TIMS}CCS: 374 Å

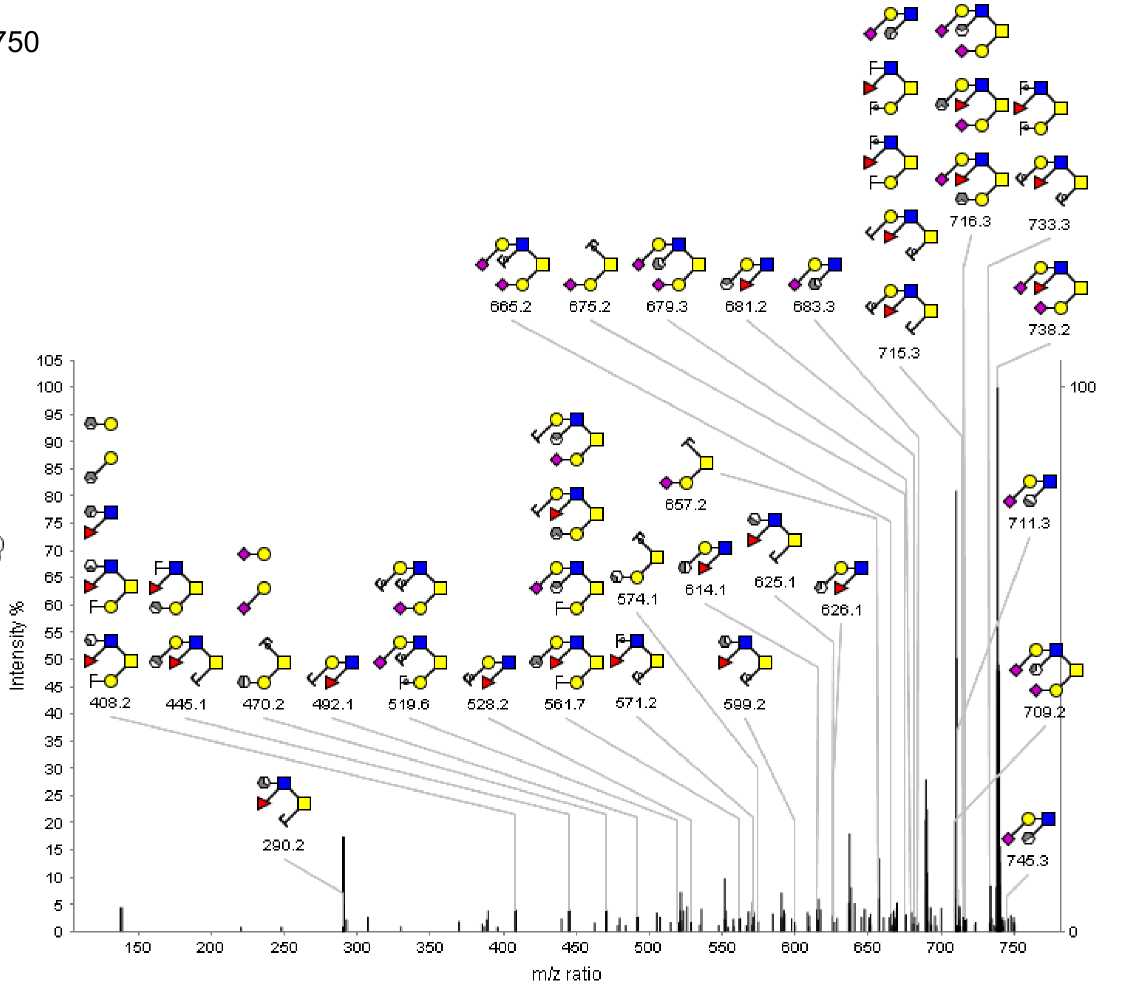

m/z range: 750-1400

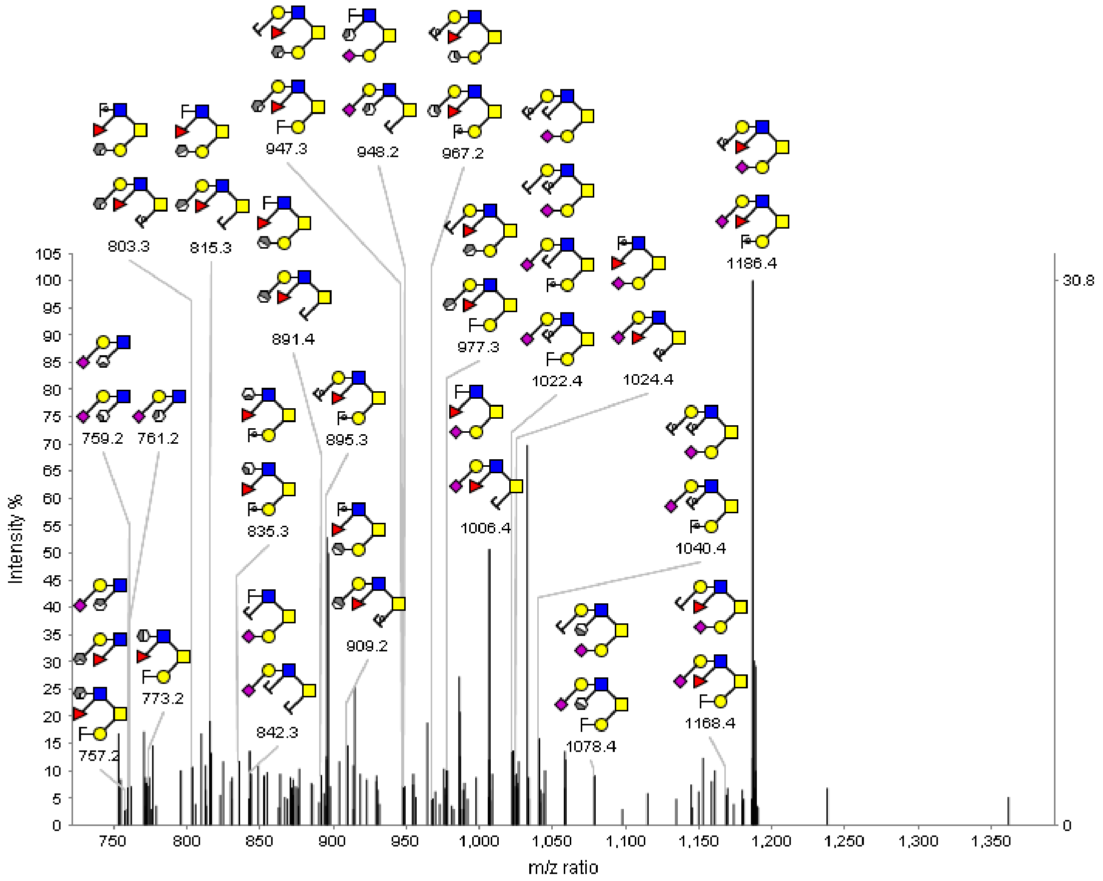

III. Annotated MS/MS spectra of *O*-glycans in cystic fibrosis sputum

Glycan code: 530-1

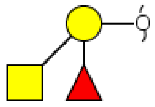

m/z: 530.21
^{TIMS}CCS: 208 Å

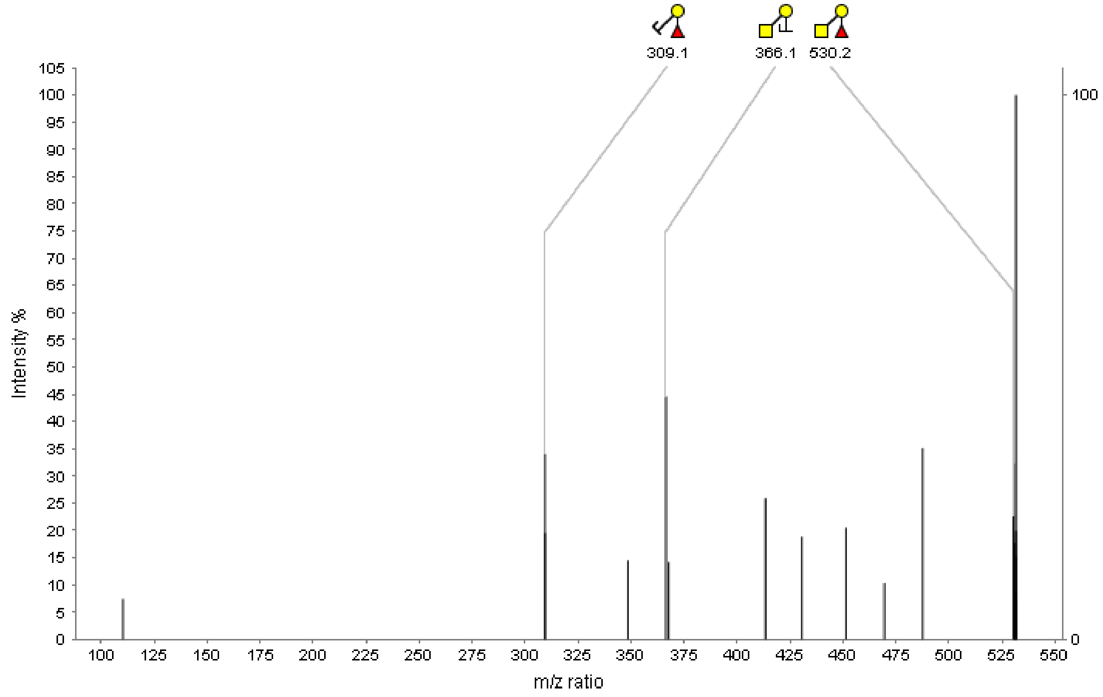

Glycan code: 530-2

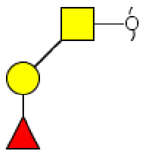

m/z: 530.21
^{TIMS}CCS: 211 Å

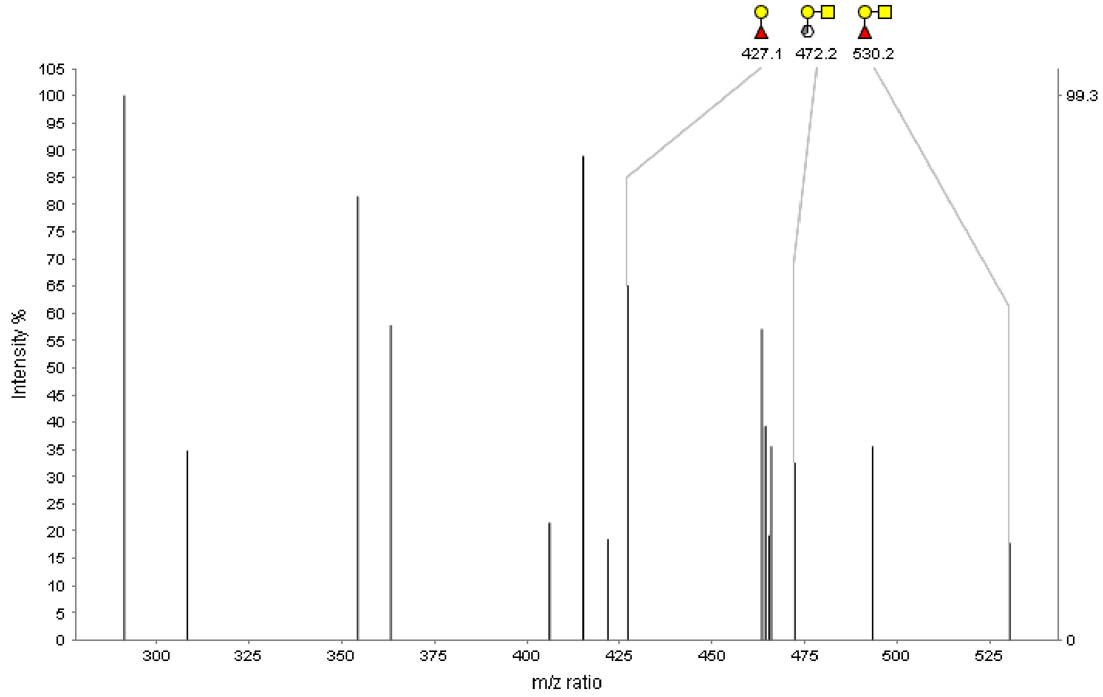

Glycan code: 587-1

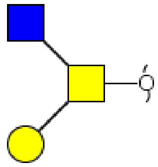

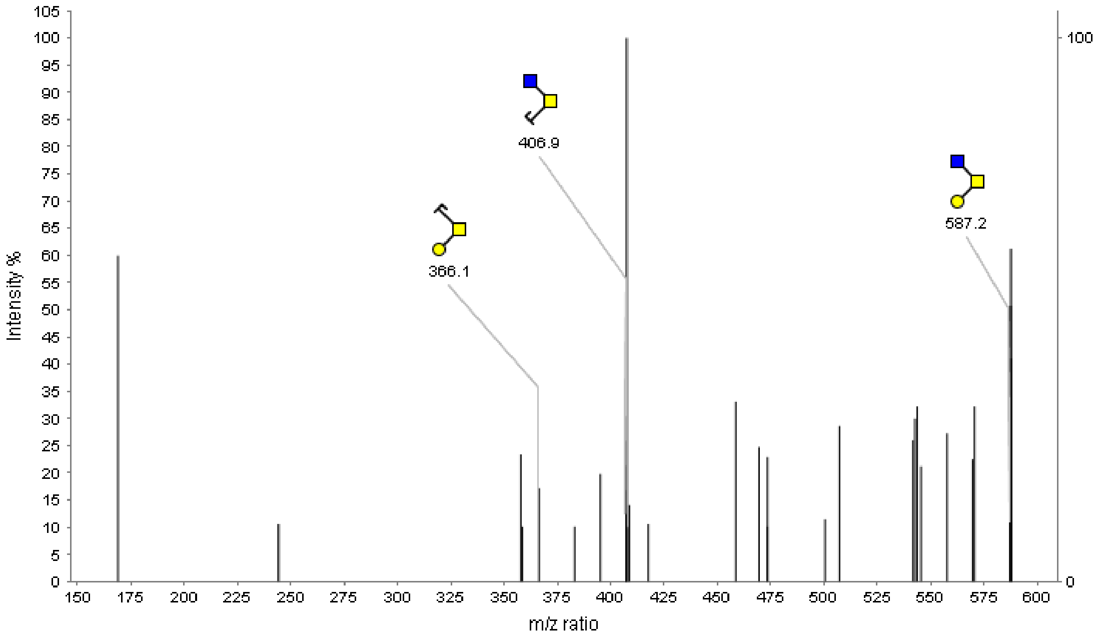

m/z: 587.23
^{TIMS}CCS: 234 Å

Glycan code: 675-1

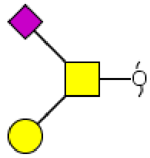

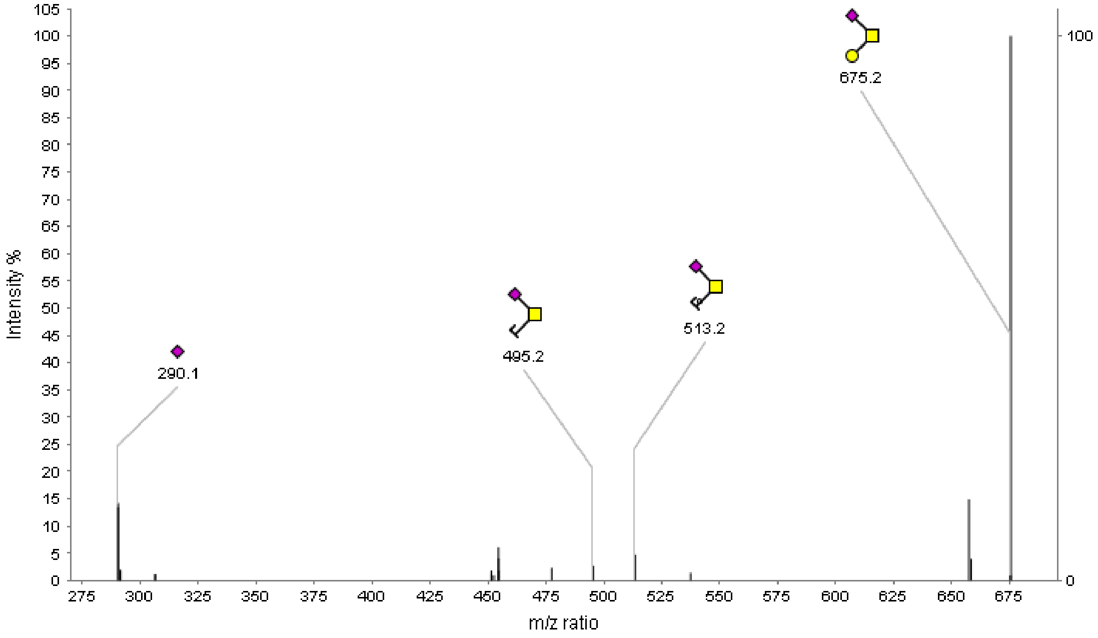

m/z: 675.25
^{TIMS}CCS: 247 Å

Glycan code: 675-2

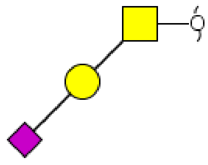

m/z: 675.25
^{TIMS}CCS: 251 Å

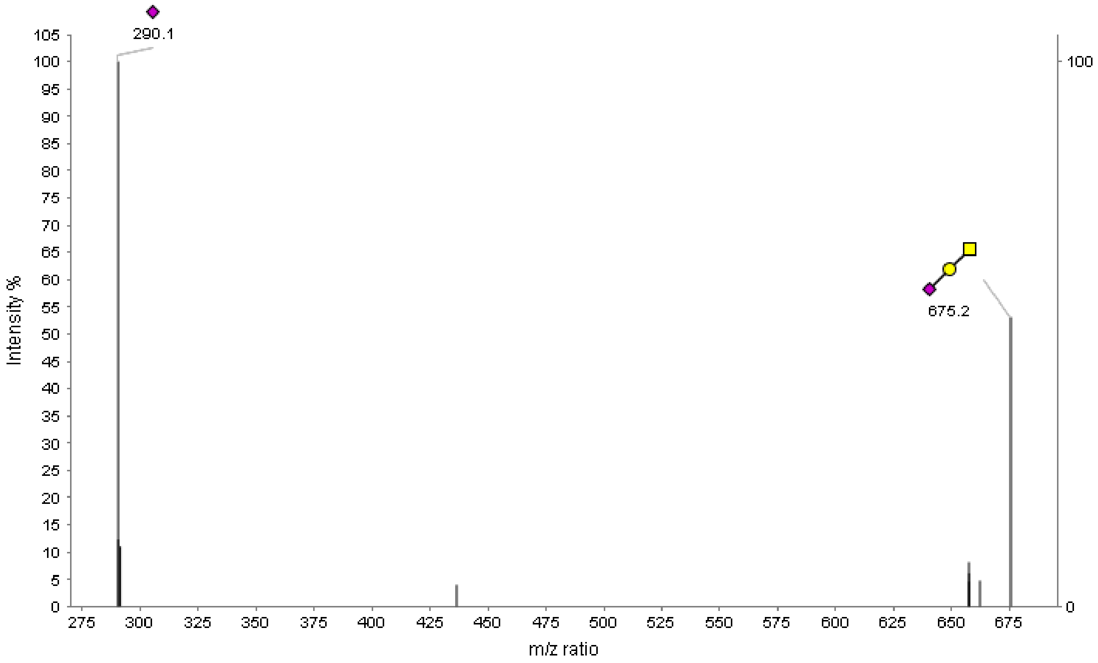

Glycan code: 716-1

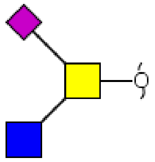

m/z: 716.27
^{TIMS}CCS: 262 Å

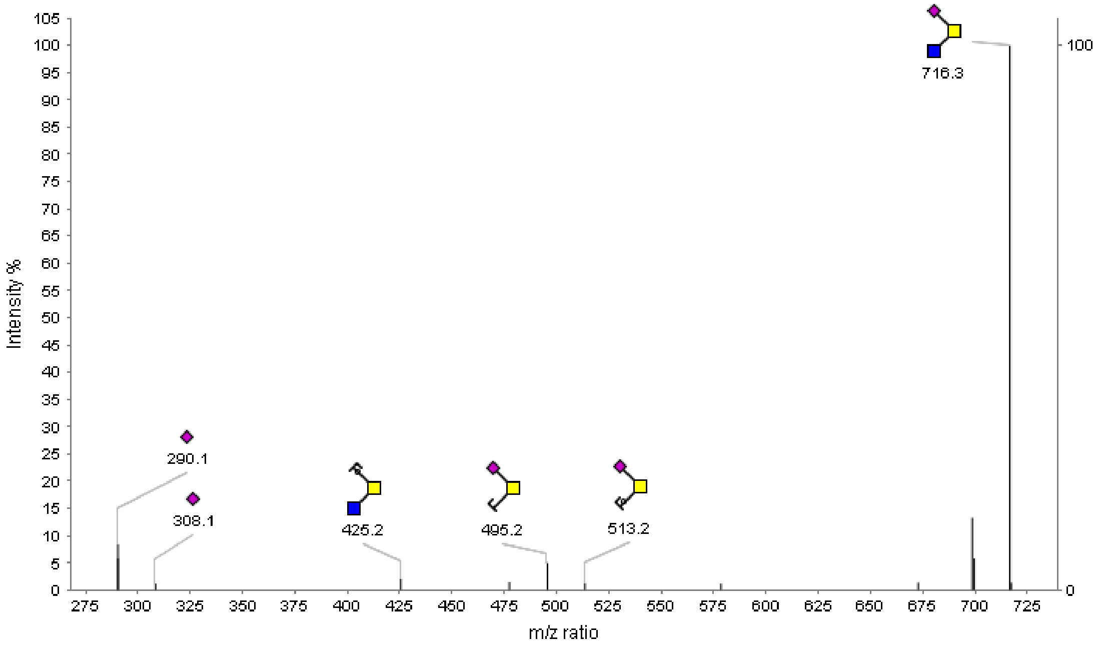

Glycan code: 733-1

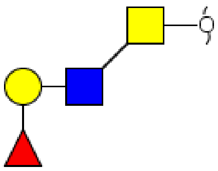

m/z: 733.29
^{TIMS}CCS: 257 Å

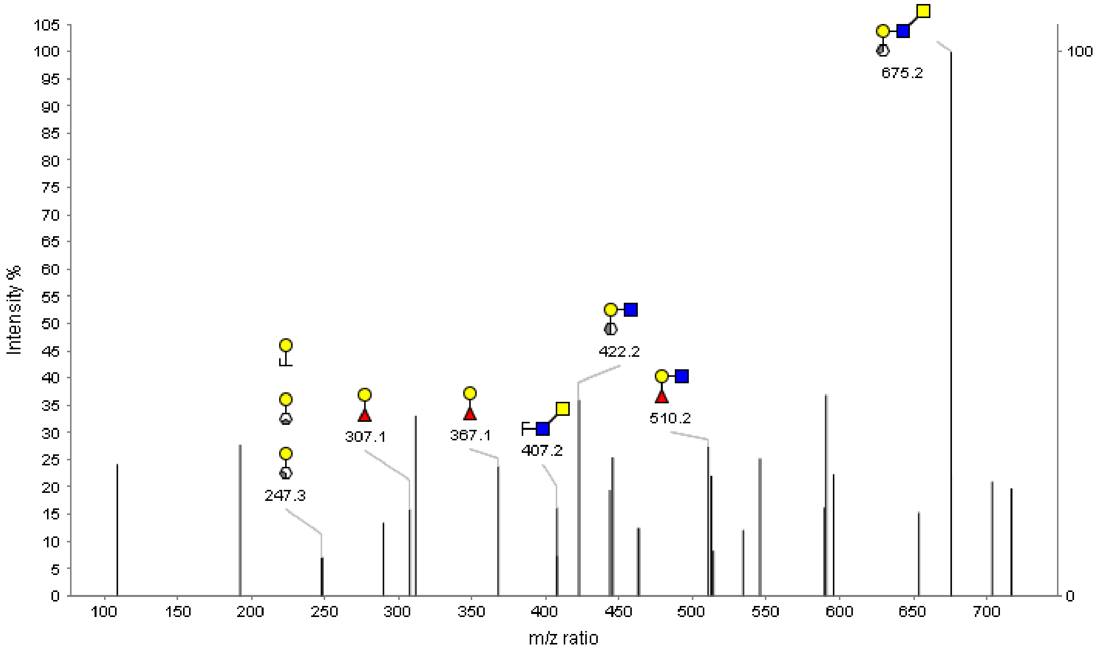

Glycan code: 749-1

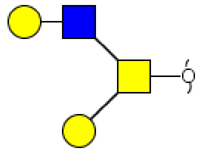

m/z: 749.28
^{TIMS}CCS: 248 Å

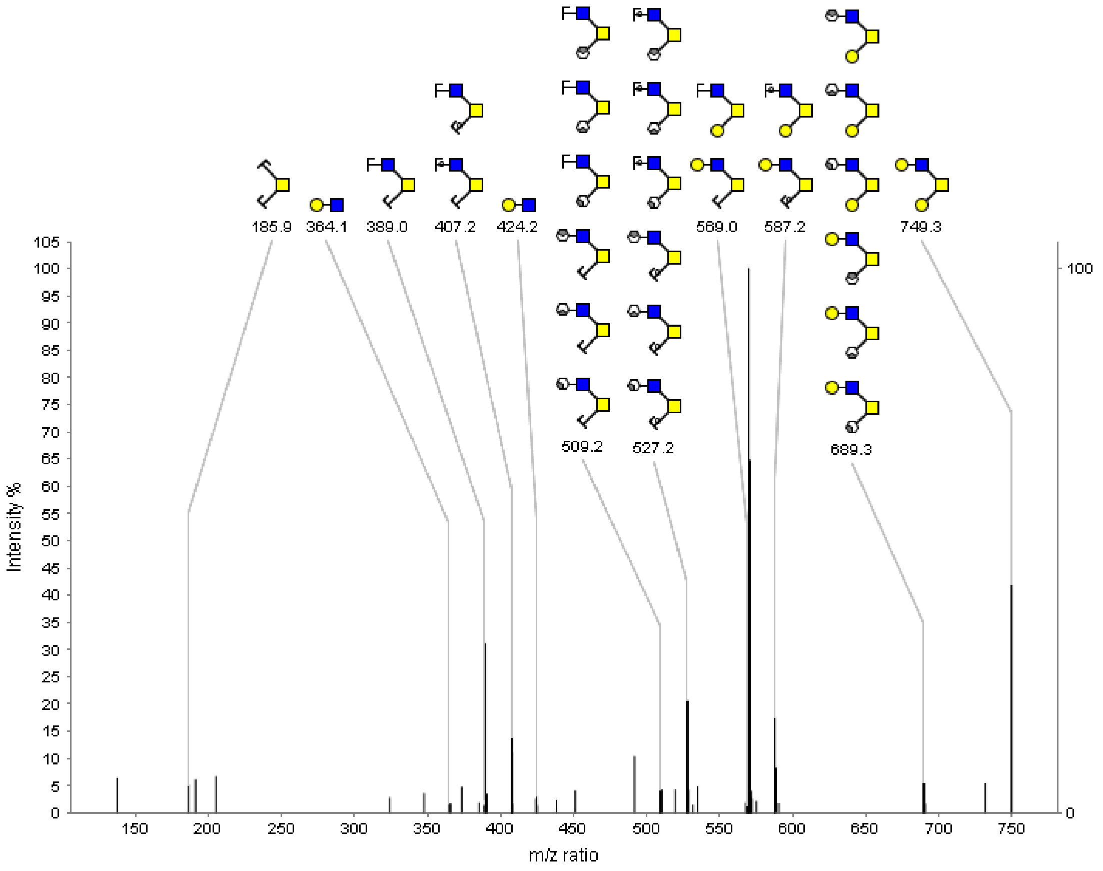

Glycan code: 821-2

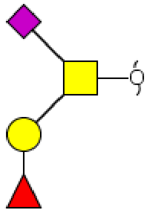

m/z: 821.30
^{TIMS}CCS: 272 Å

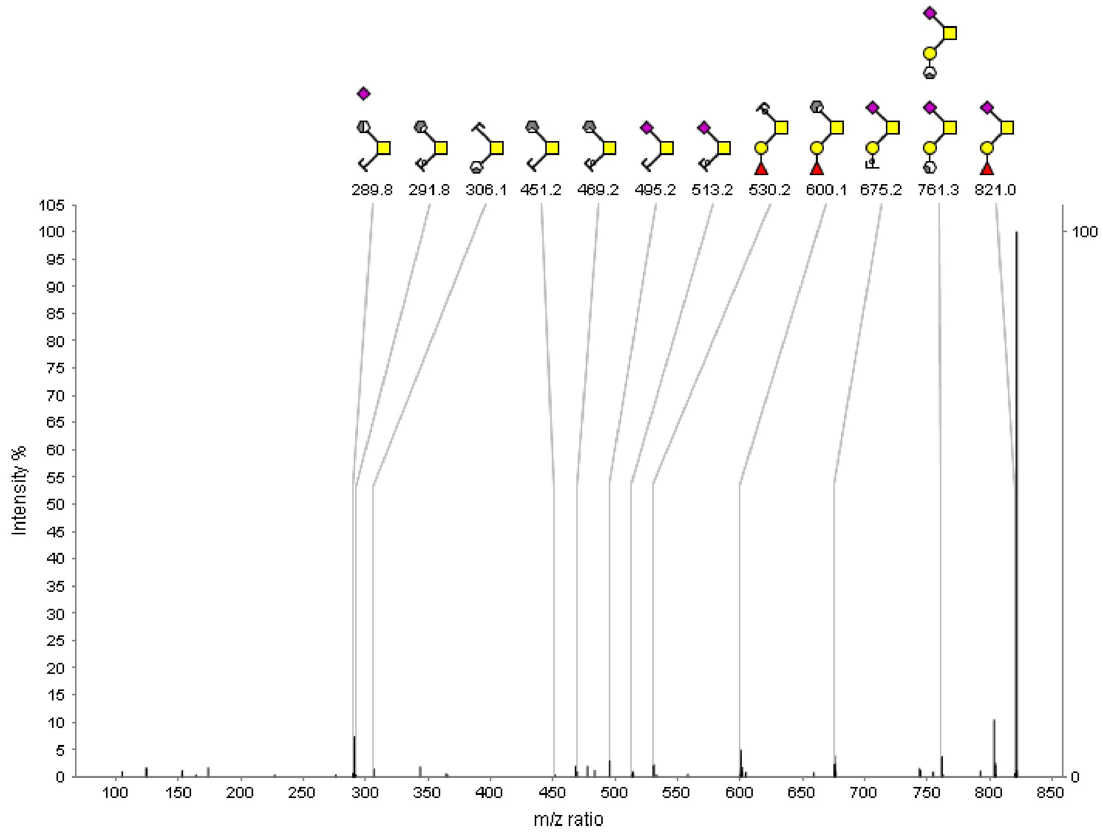

Glycan code: 966-1

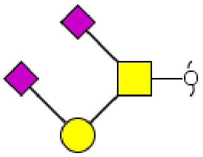

m/z: 966.34
^{TIMS}CCS: 288 Å

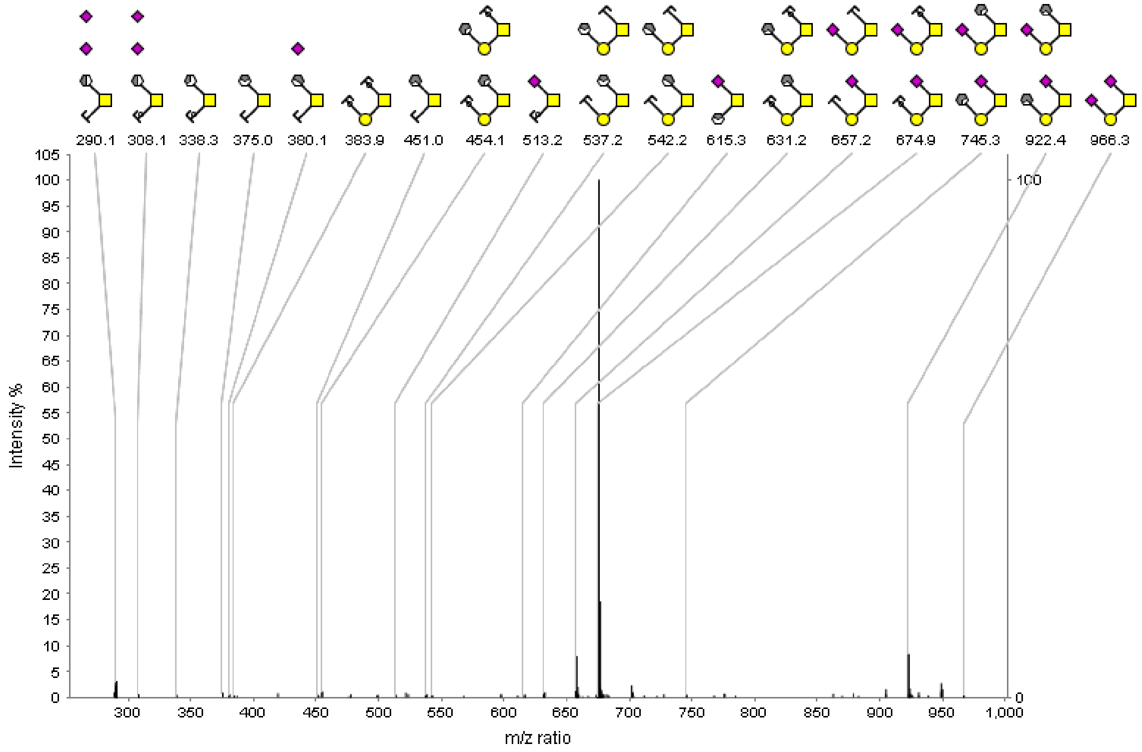

Glycan code: 966-2

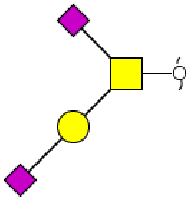

m/z: 966.34
^{TIMS}CCS: 292 Å

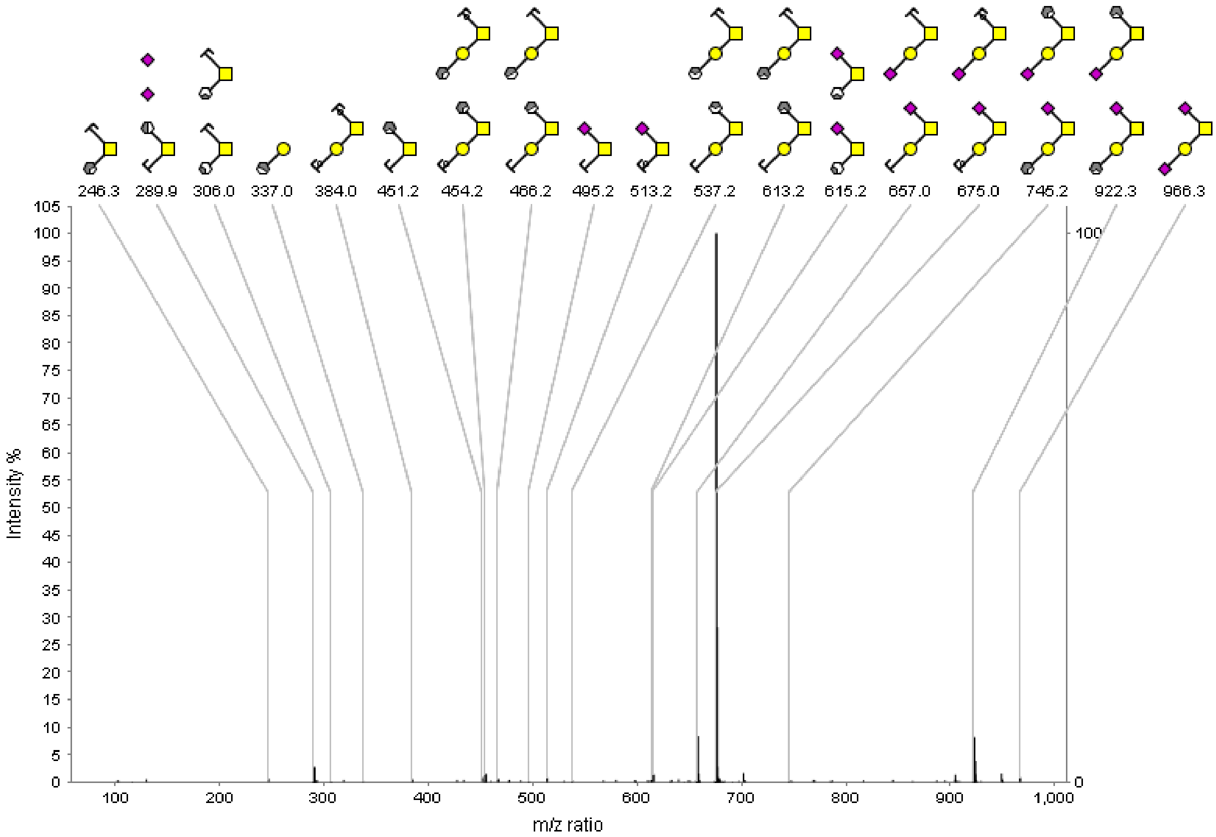

Glycan code: 1040-1

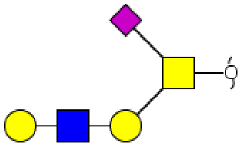

m/z: 1040.38
^{TIMS}CCS: 291 Å

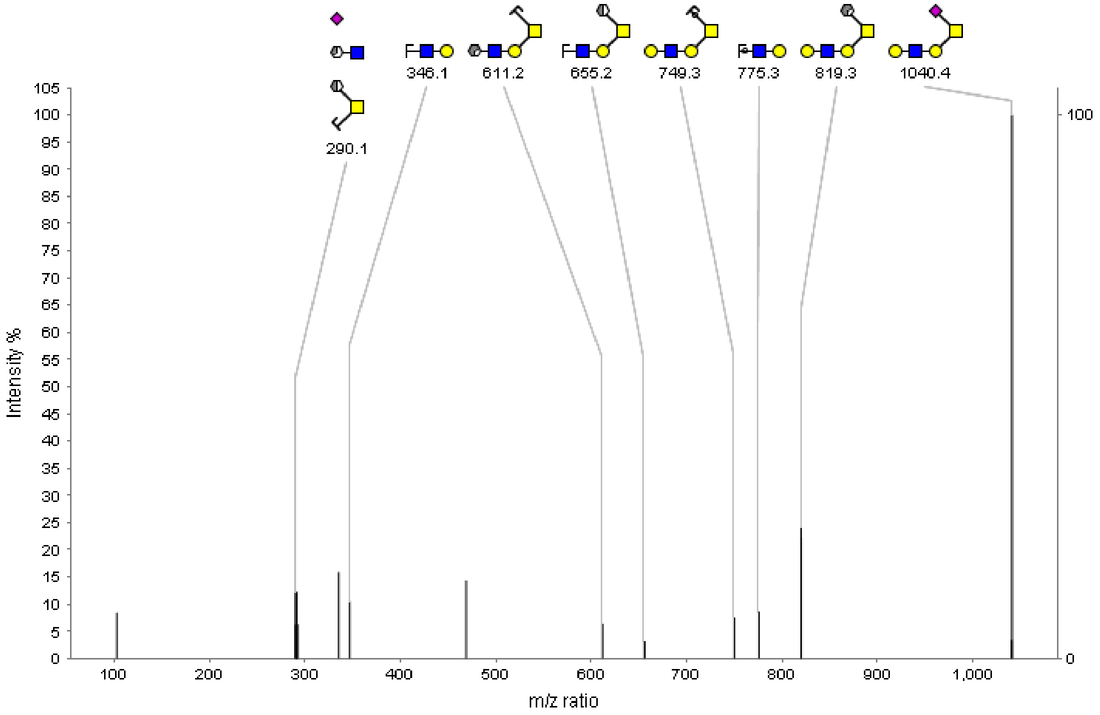

Glycan code: 1040-2

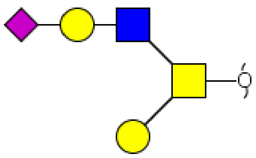

m/z: 966.34
TIMSCCS: 288

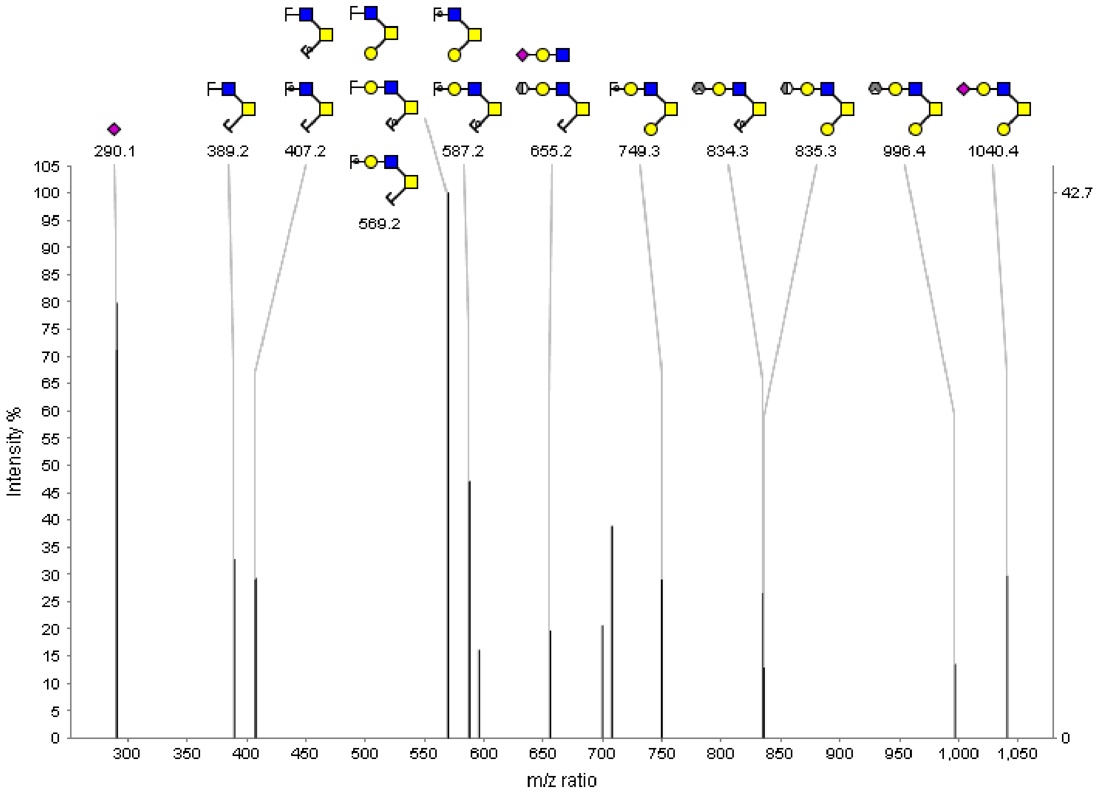

Glycan code: 1040-3

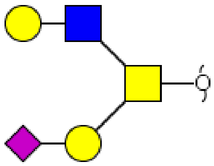

m/z: 1040.38
TIMSCCS: 306 Å

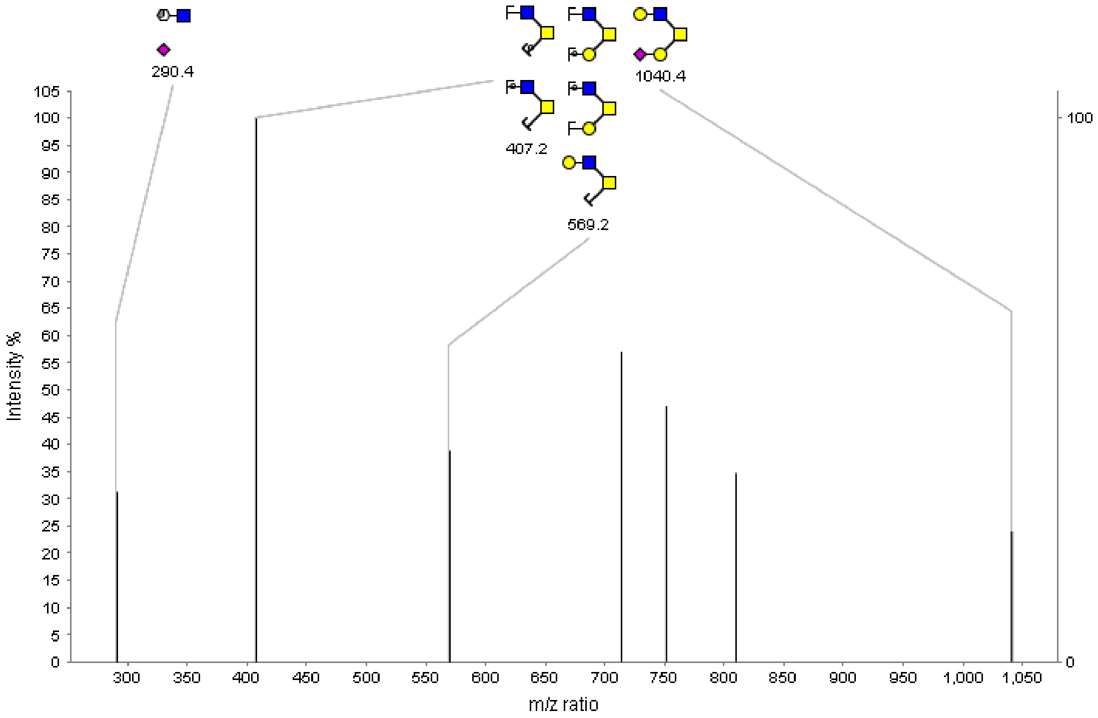

Glycan code: 1098-1

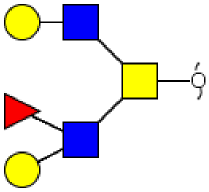

m/z: 1098.42
^{TIMS}CCS: 305 Å

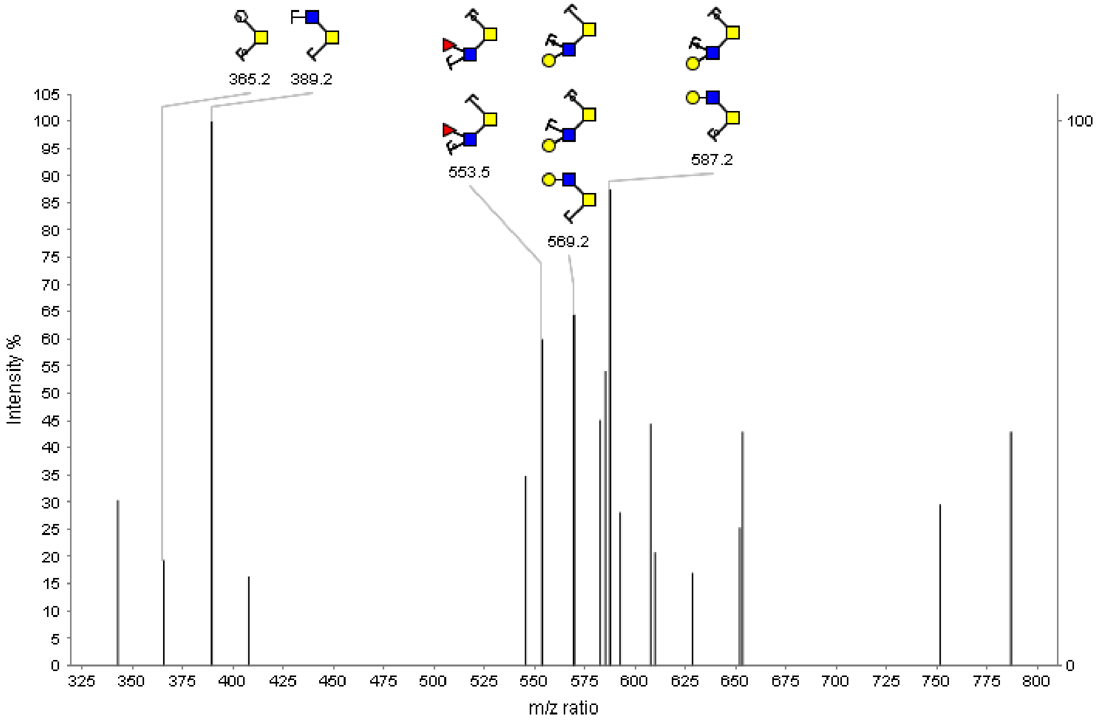

Glycan code: 1098-2

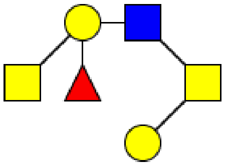

m/z: 1098.42
^{TIMS}CCS: 315 Å

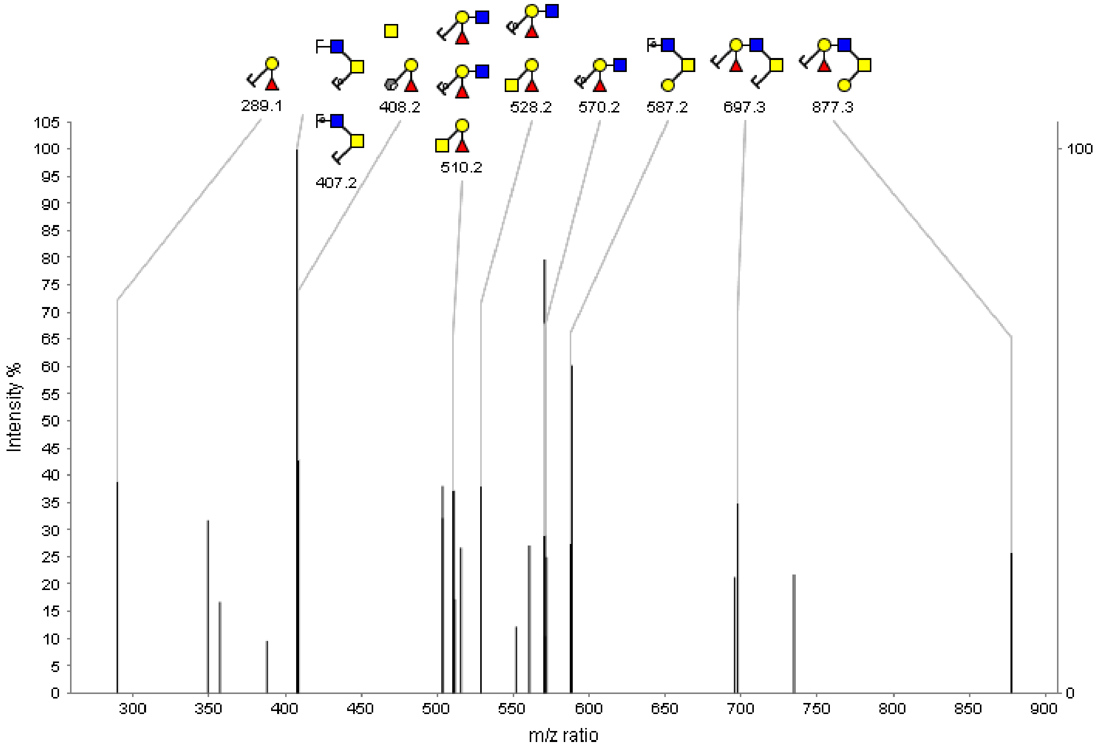

Glycan code: 592-1

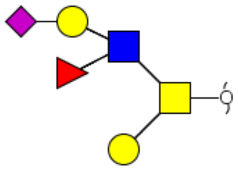

m/z: 592.71
TIMSCCS: 330 Å

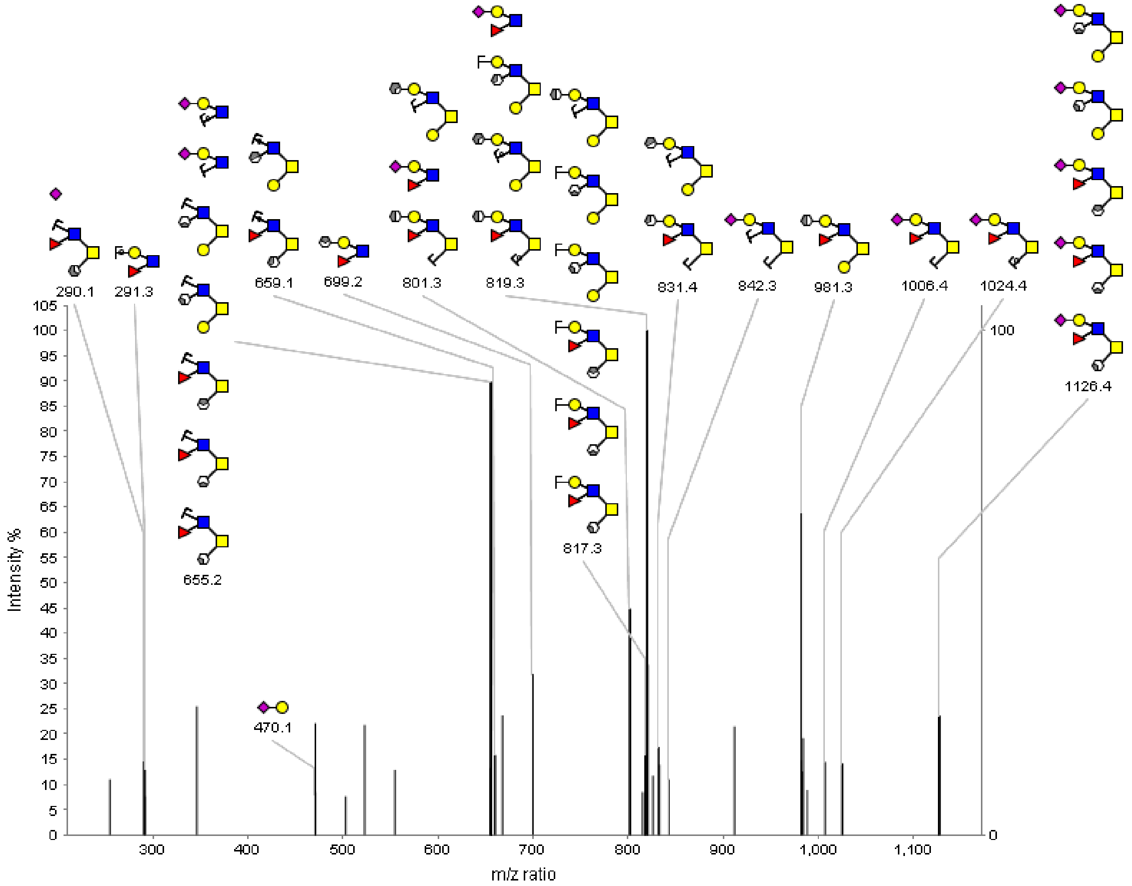

Glycan code: 592-3

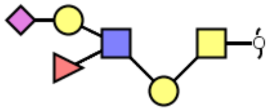

m/z: 592.71
TIMSCCS: 358 Å

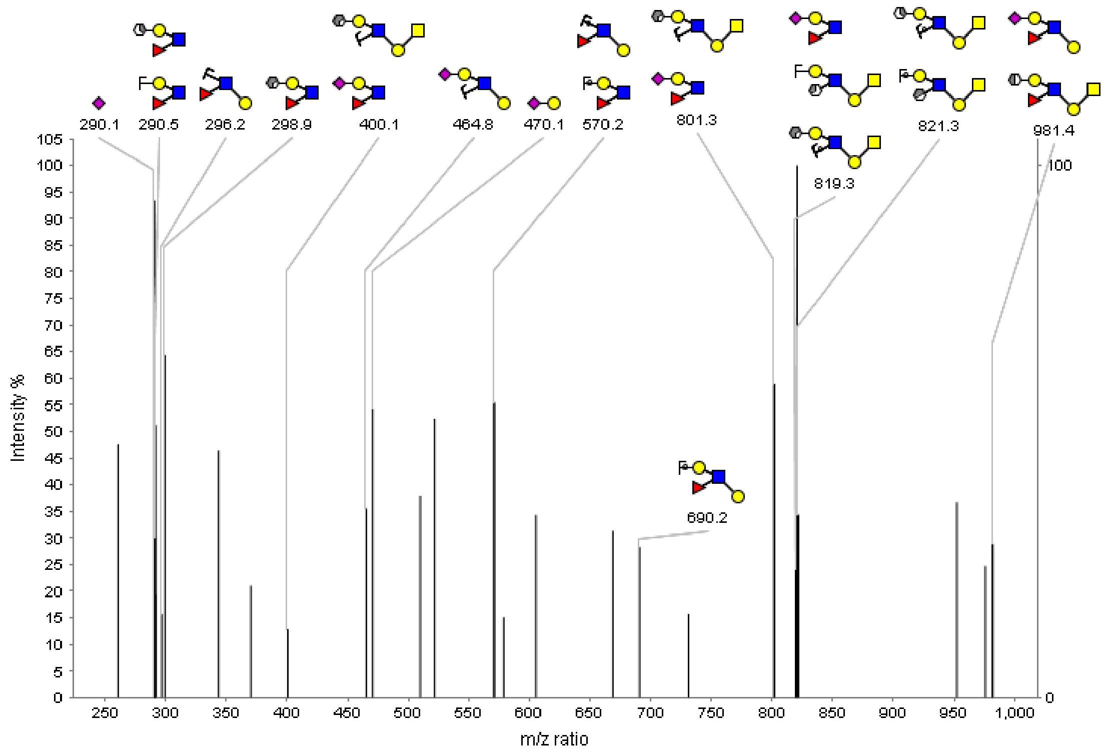

Glycan code: 592-2

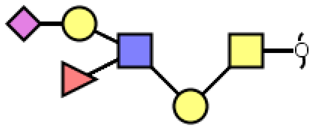

m/z: 592.71
^{TIMS}CCS: 345 Å

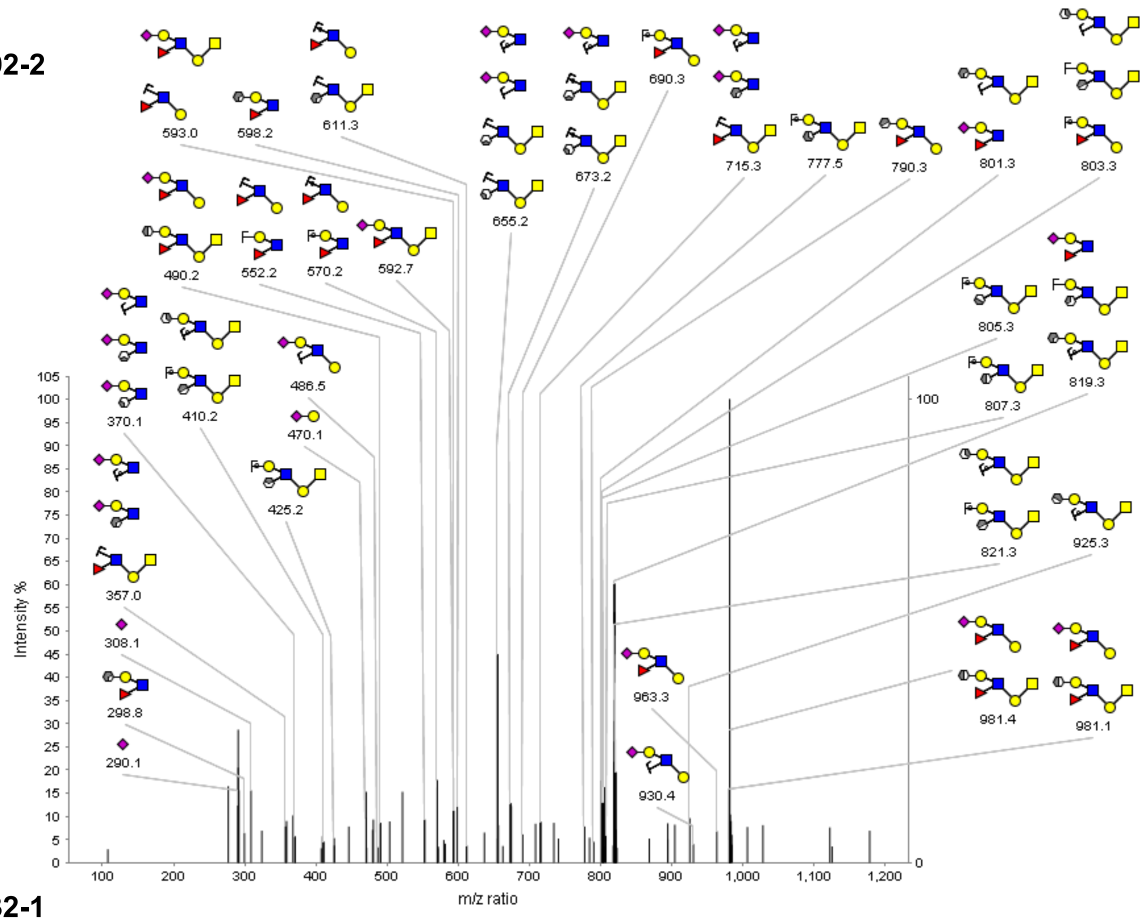

Glycan code: 632-1

m/z range: 150-750

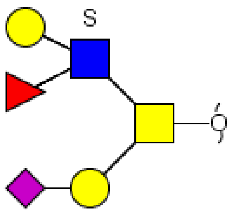

m/z: 632.69
^{TIMS}CCS: 338 Å

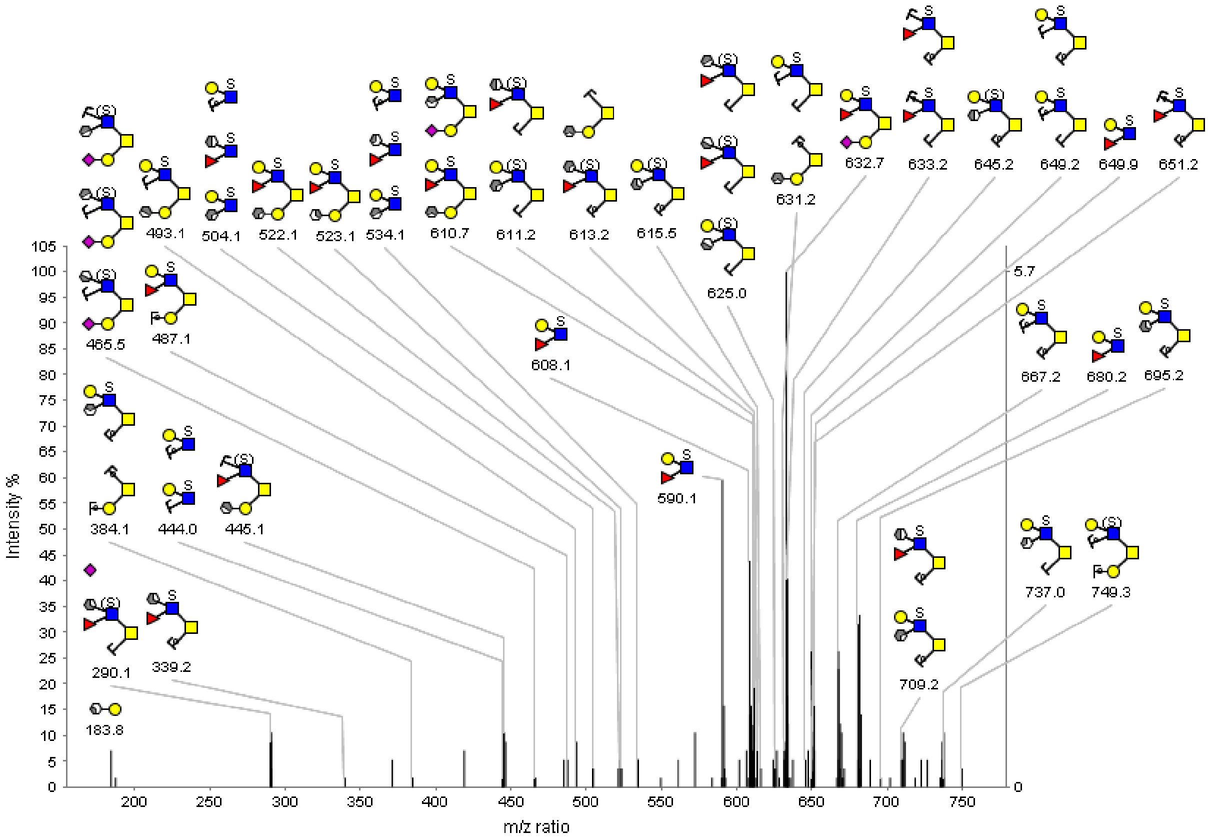

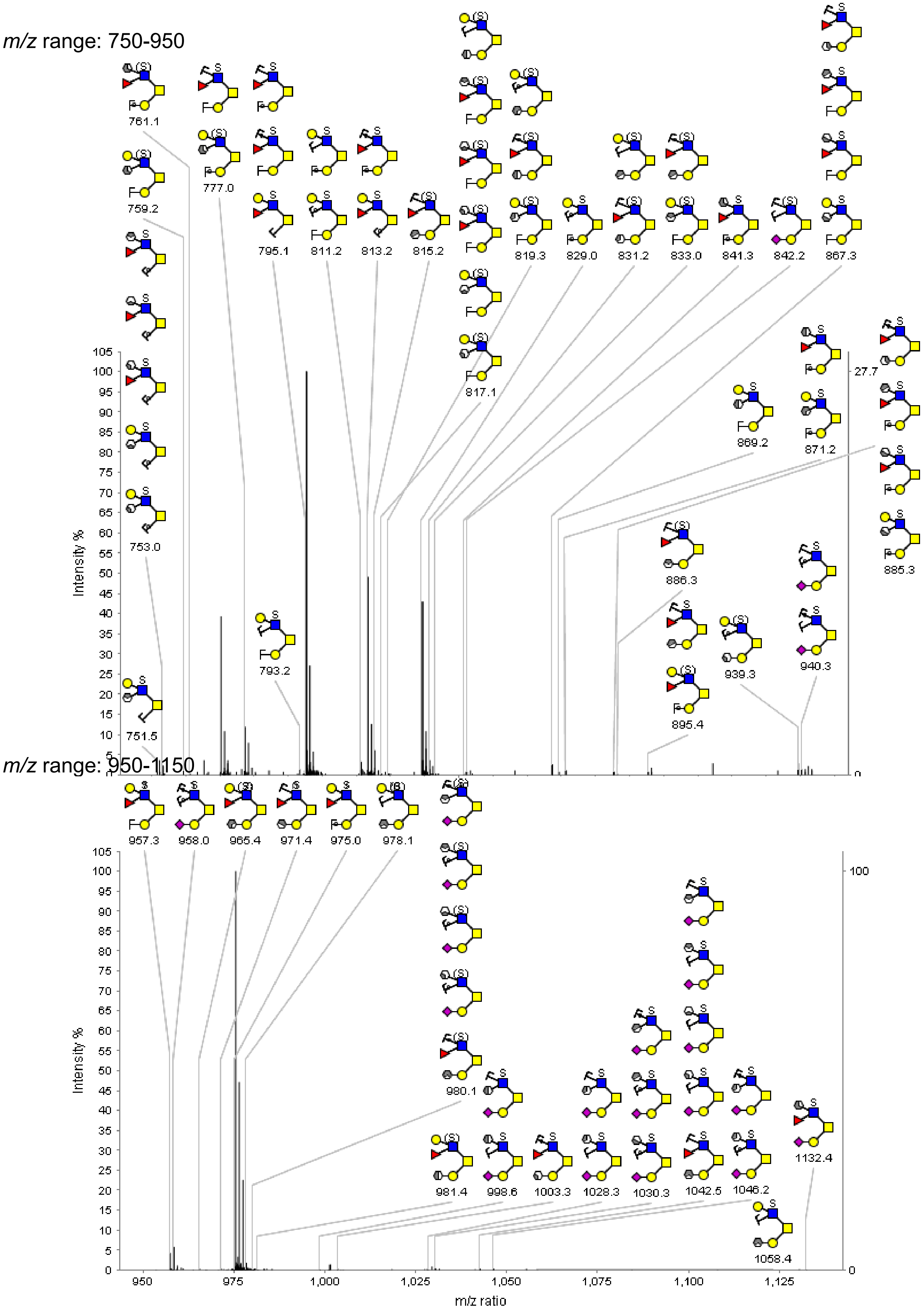

m/z range: 750-950
761.1
759.2
777.0
795.1
811.2
813.2
815.2
817.1
819.3
829.0
831.2
833.0
841.3
842.2
867.3
753.0
751.5
793.2
869.2
871.2
885.3
886.3
895.4
939.3
940.3
Intensity %
27.7
m/z range: 950-1150
957.3
958.0
965.4
971.4
975.0
978.1
980.1
981.4
998.6
1003.3
1028.3
1030.3
1042.5
1046.2
1058.4
1132.4
Intensity %
m/z ratio

Glycan code: 665-1

m/z range: 150-750

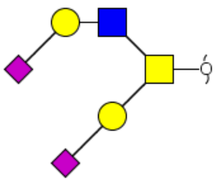

m/z: 665.23
^{TIMS}CCS: 351 Å

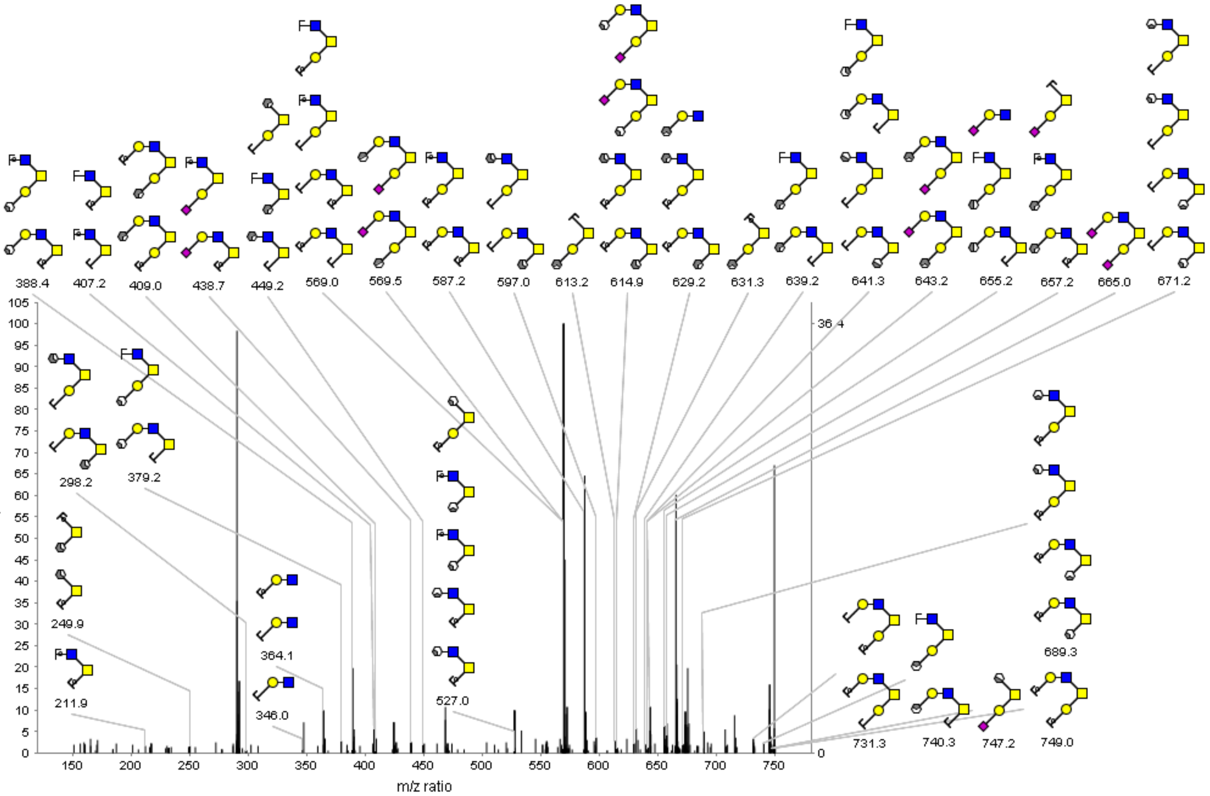

m/z range: 750-1050

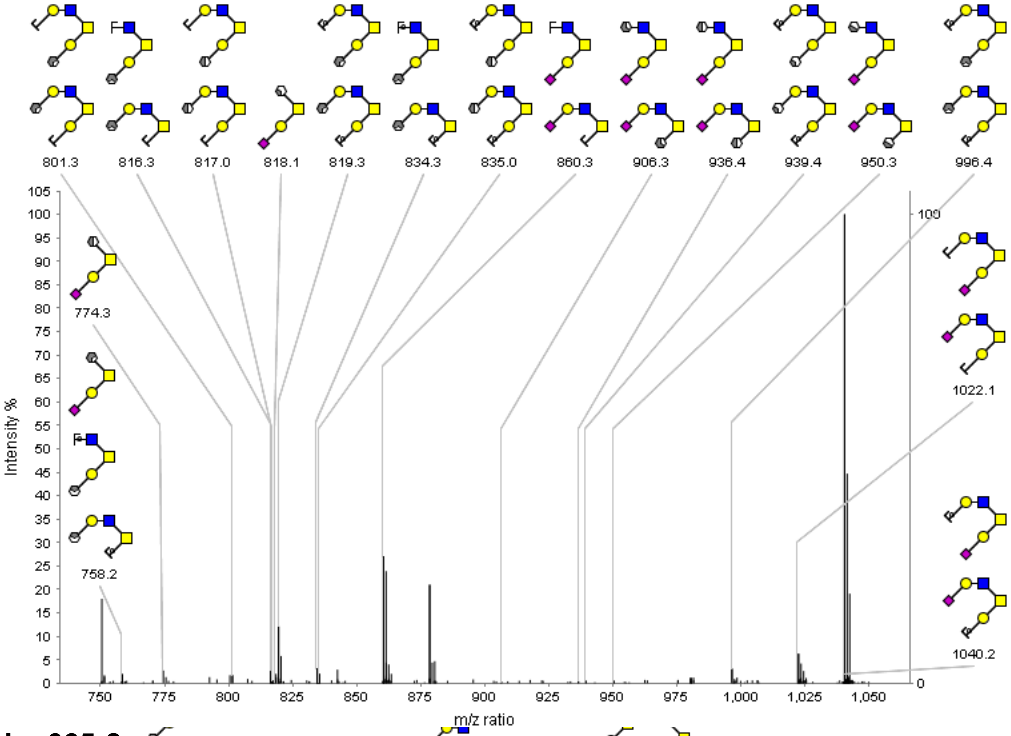

Glycan code: 665-2

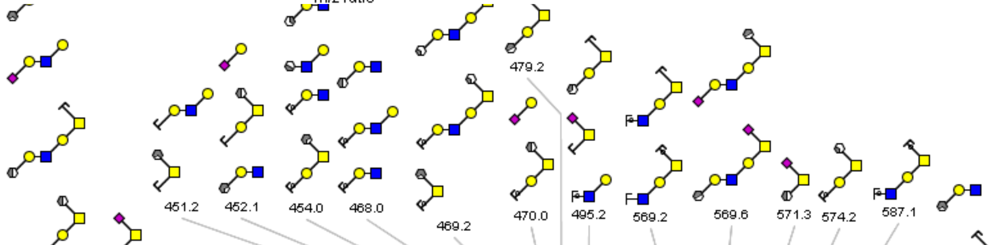

m/z range: 150-650

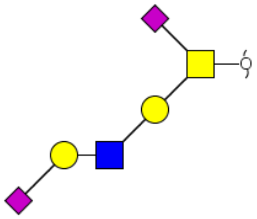

m/z: 665.23
^{TIMS}CCS: 351 Å

m/z range: 650-1150

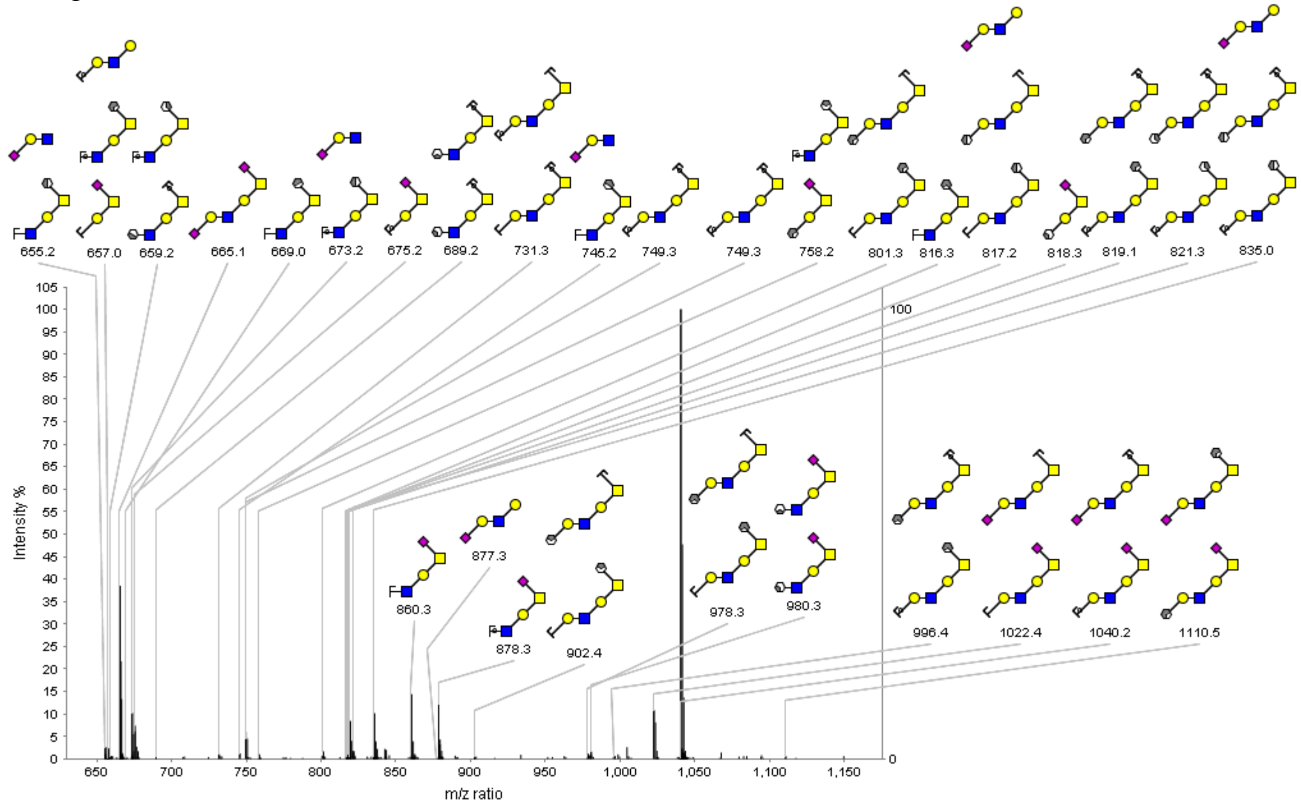

Glycan code: 738-1

m/z range: 150-750

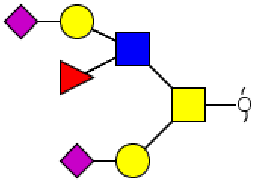

m/z: 738.26
^{TIMS}CCS: 375 Å

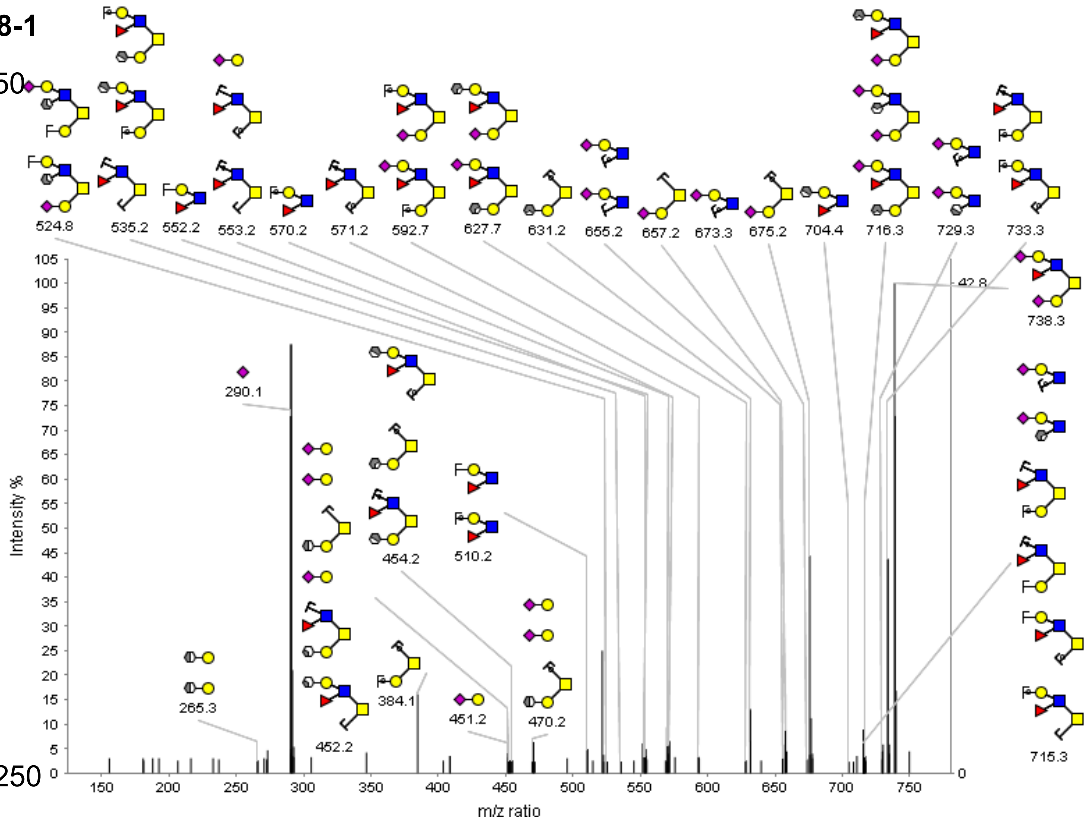

m/z range: 750-1250

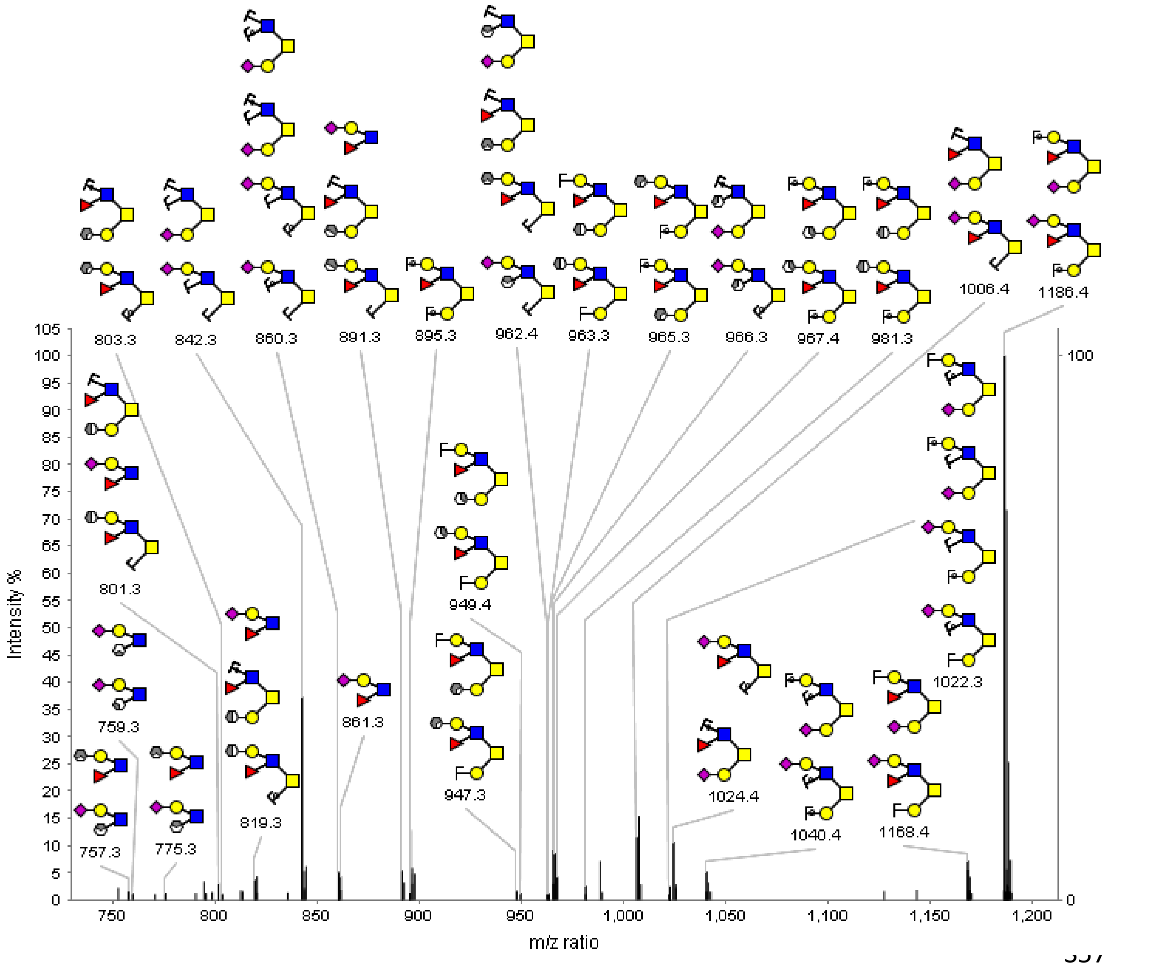

Supplement: Supplementary file 4 — Supplementary Data 1 [file 41467_2024_46825_MOESM4_ESM.pdf]
